# Position of the advisory and executive board of the German Association for Medical Education (GMA) regarding the “masterplan for medical studies 2020”

**DOI:** 10.3205/zma001254

**Published:** 2019-08-15

**Authors:** German Association for Medical Education GMA, Advisory Board

**Affiliations:** 1 Erlangen, Germany

## Background to this commentary

The “Masterplan for Medical Studies 2020” published in March 2017 came unexpected for many members of the Society for Medical Education (GMA). The document, which in part promises profound changes to admission to medical studies, medical programs’ structure and content, as well as the federal licensing exam, raised questions that were discussed intensively in the committees of the GMA. The basic thrust of the indicated readjustment of medical studies seemed to point in the right direction and some of the innovations which had been hard-won by lecturers and faculty seemed to be confirmed by the announced measures. But the GMA committees also made criticisms; that the Masterplan for Medical Studies 2020 left central terms and concepts undefined, did not fully take into account the relevant evidence for the measures and failed to address some relevant topics. 

## Process

Against this background, the Advisory Board of the GMA decided in September 2017 to write a joint commentary on the Masterplan for Medical Studies 2020 in order to stimulate internal debate, to contribute to the overall discussion through the perspective of its committees and to draw attention to possible ambiguities. The individual committees were free to decide whether and how they wanted to contribute to this project. In that sense, this commentary is not a political statement on the content of the Masterplan for Medical Studies 2020 but rather a contribution to the scientific discourse about its contents^1^. 14 committees submitted their comments by spring 2019. These, as well as the present summary, have been approved by the Advisory Board and Executive Board of the GMA. 

## An attempt at a summary

Only by severely condensing the content was it possible to summarize the commentaries of the 14 contributing committees. We invite you to read the explanations of the individual committees in their entirety, they are included as attachment 1 (in German) to this summary. The nature of the commentaries also differs significantly. A review of the evidence available for the proposed measures of the Masterplan for Medical Studies 2020 was, of course, only possible where measures have been specifically designated and desired goal and the path to it are described in equal measure. Some committees discuss the more general ideas of the Masterplan for Medical Studies 2020, while others identify gaps in the argumentation or evidence and suggest how to address them.

## Admission to studies

Regarding new rules for admission to medical studies which have been called for, the Masterplan for Medical Studies 2020 states concrete, measurable goals. The** Student Selection Committee** examines the evidence on the effectiveness of the measures mentioned. Consequently, the prognostic potential of some of the proposed selection criteria for successful completion of studies beyond the school-leaving grade is limited and the alternative measurement methods beyond knowledge tests have not been conclusively validated. The proposed additional criteria may already be taken into account in the university selection process under current legislation. The existence of suitable selection criteria for the identification of future rural doctors is questioned critically and possible ways of designing the curriculum in a way that arouses interest in rural practice among students are referred to (see attachment 2 – in German). 

## Strengthening General Practice

The **Primary Care Committee **examines the evidence on the effectiveness of the measures to strengthen General Practice formulated in the Masterplan for Medical Studies 2020. International literature shows that the effectiveness of curricular interventions to increase the attractiveness of rural areas improves if they are introduced early and in a longitudinal fashion. The Committee’s research on the effectiveness of a quota of rural doctors and student selection, as well as that of the Student Selection Committee, shows that only the rural origins of applicants may be predictive of future practice in rural areas. The Committee further lists – based on the international literature – how curricular programs should be designed to encourage career choices in General or rural practice. These program elements range from positive role models to the active involvement of students in patient care, flanked by infrastructural and didactic aspects (see attachment 3 – in German). 

## Restructuring of medical studies

Central to the planned restructuring are the competence orientation of medical studies, its practical orientation, the strengthening of General Practice and practical exams. 

The **Practical Skills Committee** welcomes the emphasis on practical skills including communication and the associated expansion of simulation-based teaching, which will not only increase the practical relevance but also contribute to greater patient safety. The Committee would like to remind that the necessary resources for the development of the skills labs will also have to be ensured for the planned clinical practical exams. The potential of interprofessional practical training formats, which has been left out of the Masterplan, should be given more consideration (see attachment 4 – in German). 

The **Communicative and Social Competences Committee** welcomes the proposed longitudinal integration of communicative and social learning outcomes in medical education and refers to content not mentioned in the Masterplan for Medical Studies 2020, such as communication in intra and multidisciplinary or intra and multiprofessional teams. It also calls for the design of this content be left to the universities themselves; especially as corresponding or similar curricula have already been successfully implemented at many teaching locations. Several arguments are made to support the position that the priority given to the “National Model Curriculum Communication” referenced in the Masterplan for Medical Studies 2020 is neither necessary nor desirable. According to the Committee, there is no evidence to justify the implementation of a uniform national curriculum for all faculties and thereby to override their respective curricular content, design or priorities (siehe attachment 5 – in German).

Particularly in the context of clinical practical skills and competences, including communicative and social learning outcomes, the use of standardized patients (SP) has proven itself in teaching and examinations and an intensification of the efforts in this area would be desirable. However, the **Standardized Patients Committee** also explains how, in the event of a sudden and intensive expansion of SP programs in teaching and assessment, special attention must be paid to compliance with the highest quality standards. Interpretation sovereignty over the curricula e.g. communicative and social competences must remain in the hands of faculties, just as they must be in defining quality standards for SP-based teaching and assessment at universities (see attachment 6 – in German). 

The **Interprofessional Education Committee** explicitly welcomes the fact that “multi-professional working in teams” is specifically mentioned in the Masterplan for Medical Studies 2020 as desirable training content. However, it also mentions that the failure to mention inter-professionalism thus ignores the need to learn and work together with people from other health professions. The Committee also explains that inter-professional examinations must become routine for a congruence of teaching, learning and assessment. The demand for national curricula is viewed critically; it would be important to integrate the programs already developed at the faculties. Moreover, the involvement of non-medical teachers ought not to be sanctioned through capacity legislation (see attachment 7 – in German).

The **Integrative Medicine and Perspective Pluralism Committee** proposes concrete ideas and didactic concepts for the design of the Masterplan for Medical Studies 2020 in order to ensure that the individuality of patients in their health and illness needs and experiences can better be taken into account in routine medical work. It also advocates the teaching of patient-centered relationship design and communication as well as the integration of practitioners’ self-care into medical studies (see attachment 8 – in German). 

The **Committee on Gender, Diversity and Career Development in Medical Education** advocates that diversity and gender competencies should be considered more extensively than is the case to date not only as regards curricular redesign but also in university didactics, assessment and student selection. With the emphasis on scientific competences in medical studies, there would now be an opportunity to integrate diversity and gender aspects here as well, so that graduates are trained regarding the need for gender-balanced study cohorts and gender-specific and diversity-sensitive data analysis in their own scientific activity. The opportunity to revise curricula as part of the Masterplan for Medical Studies 2020 should also be used to design them so that study and family become as compatible as possible, right through to part-time study models (see attachment 9 – in German). 

The **Committee for Cultural Competence and Global Health** proposes to systematically consider socio-cultural and international aspects of health, medicine and medical practice in order to systematically implement the “consistent orientation towards patients and their needs” as outlined in the Masterplan for Medical Studies 2020. In order to do justice to the socio-cultural diversity of the population, the promotion of cultural competences should be integrated into regular medical studies. There is a need for courses which have not been discussed in the Masterplan so far but which promote the ability to reflect and systematically consider social, ethical and moral issues (see attachment 10 – in German).

The **Faculty and Organizational Development Committee in Education** also welcomes many measures in the Masterplan for Medical Studies 2020 and notes that there is a need for training and development for the meaningful implementation of many of the measures. This need arises, as it were, through the planned closer integration of groups of people from the area of general practice, non-academic hospitals or non-academic health professions who to date have not been closely associated with universities. Also through the consistent implementation of longitudinal curricula and competence-oriented teaching and assessment formats. For this reason, established standards must be adhered to and local circumstances taken into account. Due to their experience and strong networks, the faculties would be capable of developing and implementing their own concepts for fleshing out the measures envisaged in the Masterplan for Medical Studies 2020, provided that the necessary funds were available for them (see attachment 11 – in German). 

The **Digitization – Technology-Assisted Learning and Teaching Committee** notes that the digitization of medicine and the entailed necessary measures for medical studies in Germany are not mentioned in the Masterplan for Medical Studies 2020. It is stated that the meaningful and comprehensive use of digital teaching and learning technologies is needed in education. In addition to concrete references to learning and teaching methods supported by technology, information technology infrastructure should also be available. Teaching staff should be better trained in media pedagogy and didactics. The Committee also urges the consistent dissemination of digital competences in health and patient care and proposes a national digitization strategy for medical studies (see attachment 12 – in German).

The **Assessment Committee** welcomes the Masterplan for Medical Studies 2020 in the hope that this will result in meaningful corrections to the examination culture in medical studies. Consistent competence orientation would first and foremost mean the integration of formative assessments into learning routines, assisted by mentoring, feedback and reflection. The Masterplan for Medical Studies 2020 may result in a curriculum watershed which will allow faculties to re-orient the way teaching, learning and examinations are synchronized. In this context, it is noticeable that the Masterplan for Medical Studies 2020, demands the teaching of e.g. communicative and scientific competencies but that these remain unmentioned as regards assessment. This omission must not lead to such content being ignored in the formative as well as summative examination canon. The promised reduction in graded course certificates is welcomed, as too many sanctioning exams preclude a learning-friendly exam culture. However, reduction in the number of course certificates must also be reflected in an actual reduction of examinations. Overall, the question is raised as to whether grading examinations is necessary at all. For example, the question of whether a student has attained the educational goal outlined in §1 Section 1 Sentence 1 of the German Medical Licensure Act is dichotomous in nature and does not require grading. Likewise, there is a call for consistent criterion-referenced definition of performance standards in assessment, this too an imperative of competence orientation. It is also stated that consistent competence orientation of medical studies questions the rationale of having a medical program’s subjects defined by traditional disciplines (see attachment 13 – in German).

To assess the success of the measures formulated in the Masterplan for Medical Studies 2020, the **Teaching Evaluation Committee** notes that common evaluation goals and methods should be agreed at an early stage. Only in this way will it be possible to reach meaningful conclusions on the effectiveness of the measures or the achievement of goals. Even before the measures of the Masterplan for Medical Studies 2020 result in any adjustments to the licensing regulations or other regulations/laws, they should be reviewed for their applicability and effectiveness, including their cost-benefit ratios. In addition, the Committee proposes approaches to evaluation for higher-level measures (competence orientation, constructive alignment) but also refers to areas where methods still need to be established for meaningful evaluation (scientific competencies). Finally, areas are identified which must be carefully evaluated under any circumstances, e.g. the quality of teaching by staff which hitherto had only been somewhat or not at all integrated into university teaching or examiner qualifications, e.g. for the federal licensing exam. The means that sufficient means to carry out these evaluations must be made available (see attachment 14 – in German).

Lastly, the **Educational Research Methodology Committee** urges a careful analysis of change processes. Since curricular interventions which are effective in one setting might not be generally effective in other settings, transfer processes should be evaluated and scientifically monitored. Since it cannot be assumed that the expertise or the means for this are available at all teaching locations, suitable additional resources for institutionalized scientific monitoring should be made available for this purpose (see attachment 15 – in German).

## Discussion

The commentaries of the committees organized by the scientific Advisory Board of the GMA show there is confidence and hope that the implementation of the measures outlined in the Masterplan for Medical Studies 2020 may not only lead to an improvement of medical studies but also health care in Germany. Setting the course towards more competence orientation, strengthening General Practice, scientific competences, communication and team work and reforming assessment, etc. allow for some optimism. Nevertheless, the Masterplan for Medical Studies 2020 leaves a lot open that in the view of the committees requires further clarification. 

It is noticeable that the central concept of competence remains undefined and thus also that of competence orientation. As guiding concepts of the restructuring of medical studies, competence-oriented training, practical training and examinations with practical relevance are mentioned but are almost tautologically determined. Working scientifically is described but not in the form of a definition, so that here too it remains open whether, for example, unnamed aspects of the role of the “scholar” in the National Competence-Based Catalogue of Learning Objectives (NKLM) are excluded or included. The ability to work in a multi-professional team should be acquired but whether interprofessional aspects are implied is not clear. Doctor-patient communication should be promoted but communication in everyday clinical life is more than mere communication between patients and medical staff. Such vagueness must be removed before implementing the measures. Furthermore, other aspects are still not mentioned. This concerns the current charged topic of the digitization of teaching and learning as well as gender, diversity and socio-cultural aspects of medical practice. A systematic examination of gaps in the Masterplan for Medical Studies 2020 could remedy this situation. 

The concrete implementation of the measures may be met with a certain confidence, as these have often already been anticipated in their intentions by faculties. Aligning medical studies with medical competences is already codified in the current version of the German Medical Licensure Act (§1 Section 1 Sentence 1) and ensuring practical relevance in teaching and examination is already a reality at many faculties with clinical examination courses in the skills labs and OSCEs. Thus, the Masterplan for Medical Studies 2020 in many places confirms what faculties have already piloted in recent years and makes this the new norm. However, interpretation sovereignty of university programs is a constitutional task of the faculties and has been implemented successfully by strong autonomous higher education institutions within the framework of the statutory regulations in curriculum design as well as in faculty examinations. However, transfer across the board will probably be a challenge for all faculties. Whether current capacities will be sufficient everywhere for an implementation of the measures must be questioned constructively. Here suitable means, for example for the initial overhead of curriculum planning, didactic training, project management, the maintenance of curricular innovations and their scientific monitoring/evaluation to successfully implement the Masterplan for Medical Education 2020 must be made available.

To make these successes visible, several committees call for a definition of indicators and targets. Before achievement of a target can be examined, establishing a baseline would be sensible, which is why work on the Masterplan for Medical Studies 2020 begins even before its implementation. In this context, it would be useful to obtain the reference values referred to by the creators of the Masterplan for Medical Studies 2020, when they point out that more patient contact, earlier practical experience and more effective handling of research results were necessary. It would be equally interesting to know how they would describe the desired target states.

The use of the term masterplan implies a work of superordinate nature, following an organizing principle, looking towards the future; a work that formulates goals and describes actions or rules on how to achieve them. In some parts, this also applies to the Masterplan for Medical Studies 2020. However, from the point of view of the committees of the GMA, relevant measures in the Masterplan for Medical Studies 2020 must be set out in much greater detail before a promising reform of medical studies in Germany can begin. 

Contributed by (in alphabetical order): Daniel Bauer, Kai Schnabel

## Note

^1^ The boundary between scientific and political perspectives was not always clear. Analyzes of the possible implications of the measures and goals at university and socio-political levels outlined in the Masterplan for Medical Studies 2020 could supplement the present work.

## Competing interests

The authors declare that they have no competing interests. 

## Supplementary Material

Explanations of the individual committees in their entirety [german]

Student Selection Committee [german]

Primary Care Committee [german]

Practical Skills Committee [german]

Communicative and Social Competences Committee [german]

Standardized Patients Committee [german]

Interprofessional Education Committee [german]

Integrative Medicine and Perspective Pluralism Committee [german]

Committee on Gender, Diversity and Career Development in Medical Education [german]

Committee for Cultural Competence and Global Health [german]

Faculty and Organizational Development Committee in Education [german]

Digitization – Technology-Assisted Learning and Teaching Committee [german]

Assessment Committee [german]

Teaching Evaluation Committee [german]

Educational Research Methodology Committee [german]

