# Supplementary material for: Position of the advisory and executive board of the German Association for Medical Education (GMA) regarding the “masterplan for medical studies 2020”
Source: GMS J Med Educ. 2019 Aug 15;36(4):Doc46. doi: 10.3205/zma001254 (PMC6737258; doi:10.3205/zma001254)
Supplement: Explanations of the individual committees in their entirety [german] [file JME-36-4-46-s-001.pdf]

Stellungnahme des Ausschusses Studierendenauswahl

Zum Thema Studierendenauswahl adressiert der Masterplan unterschiedliche Ziele, nämlich die Auswahl von Studierenden, die

i) mit hoher Wahrscheinlichkeit ihr Studium erfolgreich absolvieren.
ii) die beste Aussicht dafür bieten, gute Ärztinnen und Ärzte insbesondere in der Versorgung der Patientinnen und Patienten zu werden oder in der Wissenschaft und Forschung erfolgreich tätig zu sein.
iii) sich später als Ärzte in unterversorgten Regionen niederlassen.

Der Masterplan benennt mehrere Maßnahmen zum Erreichen dieser Ziele, u.a.:

a) Die Hochschulen sollen neben der Abiturnote mindestens zwei weitere Auswahlkriterien anwenden, die insbesondere die sozialen und kommunikativen Fähigkeiten sowie die Leistungsbereitschaft einbeziehen.
b) Unterstützung der Hochschulen bei der Anpassung ihrer Auswahlverfahren.
c) Weiterentwicklung von Zulassungsverfahren in der Weise, dass die ärztliche Versorgung in unterversorgten Regionen verbessert wird. Hierzu sollen bis zu 10% der Medizinstudienplätze an Bewerberinnen und Bewerber vergeben werden, die sich verpflichten, nach Abschluss des Studiums und der fachärztlichen Weiterbildung für bis zu zehn Jahre in der hausärztlichen Versorgung tätig zu sein.

Mit unserem Beitrag möchten wir aufzeigen, für welche Auswahlverfahren bereits Evidenzen vorliegen, dass sie zum angestrebten Erfolg führen.

Ad i) Studienerfolg

Da sich Studienerfolg relativ gut messen lässt, ist hier die Datenlage auch am übersichtlichsten: So haben sich weltweit für die Vergabe von Studienplätzen in der Humanmedizin kognitive Verfahren durchgesetzt, deren Ergebnisse mit hoher Wahrscheinlichkeit den erfolgreichen Abschluss des Studiums vorhersagen. Hier belegen nationale und internationale Studien, dass die Note der Hochschulzugangsberechtigung die größte Vorhersagekraft besitzt [1], [2]. Allerdings gibt es in zunehmendem Umfang Probleme durch eine stark steigende Zahl von Abiturienten mit Bestnoten und durch eine mangelnde Vergleichbarkeit der Abiturnoten z.B. in unterschiedlichen Bundesländern, weshalb das Bundesverfassungsgericht einen Ausgleichsmechanismus fordert [3]. Inkrementellen Erkenntnisgewinn bieten kognitive Tests, die auf einem Kontinuum eher wie der HAM-Nat Kenntnisse oder wie der TMS eine fachspezifische Studierfähigkeit prüfen [4], [5]. Nationale Studien zeigen eine prädiktive Validität von TMS und HAM-Nat für den kognitiven Studienerfolg in den ersten Semestern [6]. Die internationale Studienlage wertet Kenntnistests dabei höher als Fähigkeitstests. Prädiktive Validität und Fairness müssten jedoch für jeden einzelnen Test erneut nachgewiesen werden [1], [7]. Es gibt keine Studien, die eine Korrelation von vorheriger medizinnaher Berufstätigkeit, sozialem Engagement, Preisen im Bildungsbereich oder sportlichen Leistungen mit dem Studienerfolg zeigen.

Ad ii) Kompetenzen für die Patientenversorgung

Kognitive Fähigkeiten, die für die Patientenversorgung oder wissenschaftliches Arbeiten benötigt werden, werden auch in den unter i) genannten Verfahren adressiert. Bei den für den Arztberuf nötigen sozialen und kommunikativen Kompetenzen stehen vor allem die Kommunikations- und Teamfähigkeit im Fokus, die derzeit mittels klassischer Interviews,

multipler Mini-Interviews (MMI) im Sinne eines Interview-Parcours oder Situational Judgement Tests (SJT) erfasst werden sollen. Alle Messverfahren bergen die Gefahr, dass durch sozial erwünschte Antworten die Ergebnisse verfälscht werden. Zudem sind Aussagen zur prädiktiven Validität schwierig, da in Studium und Beruf kaum quantitative Daten zur psychosozialen Kompetenz erhoben werden. Weitere Probleme sind mangelnde Reliabilität klassischer Interviews und unklare Konstruktvalidität von Multiplen Mini-Interviews (MMI) und SJT [6]. Dennoch befinden sich SJT im Bereich der Studierendenauswahl bereits in Großbritannien, Belgien und Kanada im Einsatz und lassen wie die MMI moderate Zusammenhänge zwischen der jeweils adressierten Kompetenz und verwandten Studieninhalten z.B. in OSCE-Prüfungen erkennen [8-10]. Diese Ergebnisse ermutigen, weitere Anstrengungen in die Entwicklung theoretischer Erklärungsansätze zu investieren. Tatsächlich sind die Entwicklung von MMIs und SJTs Schwerpunkte in einem Forschungsprojekt, mit dem das BMBF die Fakultäten bei der Erforschung und Anpassung ihrer Auswahlverfahren im Rahmen des Masterplan Medizinstudium 2020 unterstützt. Auch hier gibt es keine Studien, die eine Korrelation von vorheriger medizinnaher Berufstätigkeit, sozialem Engagement, Preisen im Bildungsbereich oder sportlichen Leistungen mit sozialen oder kommunikativen Kompetenzen in Studium oder Arztberuf zeigen.

Zur Evaluation der verschiedenen Auswahlverfahren für Studienbewerber in der Humanmedizin sei hier auf einen kürzlich erschienenen Übersichtsartikel verwiesen [6].

Ad iii) Flächendeckende hausärztliche Versorgung

Medizinisch unterversorgte Regionen stellen ein weltweites Problem dar [11], auf das Japan bereits in den 1960er und 70er Jahren mit dem flächendeckenden Ausbau medizinischer Fakultäten – eine für jede der 47 Präfekturen – reagiert hat. Weitere Maßnahmen waren 1972 die Gründung einer Fakultät, in der ausschließlich „Landärzte“ ausgebildet wurden, sowie eine Quotenregelung, die aktuell 17% aller Studienplätze für angehende Landärzte bereitstellt [12]. Die Literatur belegt, dass die Abschlussrate in der Landarztquote mit 96,3% in den Jahren 2014 und 2015 über der Abschlussrate der übrigen Absolventen von 94,2% im gleichen Zeitraum lag und damit keinen Grund für die Annahme liefert, dass durch die Quotenregelung weniger erfolgreiche Studierende zugelassen werden [13]. In Australien wurde seit 2004 ein Viertel der Medizinstudienplätze unter der Auflage, bis zu 22 Jahre in ländlichen Regionen zu arbeiten, vergeben. Umfragen zeigen, dass etwa 25% der Studierenden ein „Herauskaufen“ aus dem Programm nach Studienabschluss erwägen. Weitere Probleme gibt es bei der Definition der ländlichen Regionen, der Facharztwahl der angehenden Ärzte, der Überwachung der Verpflichtung, der Komplexität der Maßnahme und mit einer Stigmatisierung der „Landarztstudierenden“ gegenüber den ungebundenen. Dennoch empfahl ein Regierungsbericht eine Weiterführung des (reformierten) Programms [14]. Über die spätere Verteilung fertiger Ärzte in unterversorgte Regionen liegen jedoch weder in Australien noch in Japan Daten vor. Eine systematische Übersicht der World Health Organization zeigt, dass in über 70 Staaten Programme mit einer Verpflichtung zur Tätigkeit in ländlichen Regionen existieren [15]. Auch wenn systematische Vergleiche zwischen Staaten mit und ohne Verpflichtungsprogrammen noch fehlten, könnten Anreizprogramme dennoch zur Versorgung unterversorgter Gebiete beitragen.

Neben der Einführung einer Quotenregelung gibt der Masterplan das Ziel vor, Studierende auszuwählen, die sich später als Ärzte in unterversorgten Regionen niederlassen. Eine internationale Metaanalyse zu gezielten Maßnahmen, Ärzte in ländlichen und unterversorgten Regionen anzusiedeln, zeigt als einziges Erfolg vorhersagendes Auswahlkriterium für angehende „Landärzte“ die eigene Herkunft vom Land auf [16]. Andere

zielführende Maßnahmen – wie z.B. längere Ausbildungsphasen in unterversorgten Regionen – lagen nicht in der Studierendenauswahl, sondern waren in der Struktur des jeweiligen Curriculums verankert [17].

Zusammenfassend weist der aktuelle Forschungsstand Abiturnote und Eignungstests eine gute prädiktive Validität in Bezug auf den kognitiven Studienerfolg zu. Die Auswahl mittels dieser Kriterien ermöglicht die Umsetzung des 1. Masterplanziels zum hohen Studienerfolg. Die wissenschaftliche Evidenz für den Einsatz von Auswahlverfahren zur sozialen und kommunikativen Kompetenz ist schwächer: Während klassische, eher unstrukturierte Interviews ungeeignet erscheinen, müssen Hinweise auf die Validität von MMI und SJT durch weitere Studien konsolidiert werden. Für die Auswahl angehender Landärzte gibt es jenseits der eigenen ländlichen Herkunft keine Evidenzen dafür, dass irgendein Auswahlkriterium die nachhaltige Niederlassung in unterversorgten Regionen vorhersagt [18]. Eine Auswahl nach „Herkunft vom Land" verbietet jedoch das Grundgesetz. Für die Effizienz einer Landarztquote gibt es keine gesicherte Evidenz.

Die Etablierung, Durchführung und Validierung der im Masterplan geforderten Auswahlverfahren wie z.B. deutschlandweit zentral durchgeführter kognitiver Tests und SJTs oder fakultäre Interviews sind kostenintensiv. Wie im Masterplan vorgesehen, werden die Fakultäten sie nur mit zusätzlicher Unterstützung einsetzen können.

Beigetragen von (alphab.): Wolfgang Hampe, Brigitte Müller-Hilke

Literaturverzeichnis

1. Patterson F, Knight A, Dowell J, Nicholson S, Cousans F, Cleland J. How effective are selection methods in medical education? A systematic review. Med Educ. 2016;50(1):36-60. doi: 10.1111/medu.12817
2. Trapmann S, Hell B, Weigand S, Schuler H. Die Validität von Schulnoten zur Vorhersage des Studienerfolgs-eine Metaanalyse. Z Päd Psychol. 2007;21(1):11-27.
3. Bundesverfassungsgericht. Urteil des Ersten Senats am Bundesverfassungsgericht zum Numerus clausus im Medizinstudium vom 19.12.2017. Karlsruhe: Bundesverfassungsgericht; 2017. Zugänglich unter/available from: https://www.bundesverfassungsgericht.de/SharedDocs/Entscheidungen/DE/2017/12/ls20171219_1bvl000314.html
4. Hell B, Trapmann S, Schuler H. Eine Metaanalyse der Validität von fachspezifischen Studierfähigkeitstests im deutschsprachigen Raum. Emp Pädagogik. 2007;21(3):251-270.
5. Hissbach JC, Klusmann D, Hampe W. Dimensionality and predictive validity of the HAM-Nat, a test of natural sciences for medical school admission. BMC Med Educ. 2011;11(1):83. doi: 10.1186/1472-6920-11-83
6. Schwibbe A, Lackamp J, Knorr M, Hissbach J, Kadmon M, Hampe W. Selection of medical students: Measurement of cognitive abilities and psychosocial competencies. Bundesgesundheitsblatt Gesundheitsforschung Gesundheitsschutz. 2018;61(2):178-186. doi: 10.1007/s00103-017-2670-2
7. Harris BH, Walsh JL, Lammy S. UK medical selection: lottery or meritocracy? Clin Med. 2015;15(1):40-46. doi: 10.7861/clinmedicine.15-1-40
8. Dore KL, Reiter HI, Kreuger S, Norman GR. CASPer, an online pre-interview screen for personal/professional characteristics: prediction of national licensure scores. Adv Health Sci Educ Theory Pract. 2017;22(2):327-336. doi: 10.1007/s10459-016-9739-9

9. Lievens F. Adjusting medical school admission: assessing interpersonal skills using situational judgement tests. Med Educ. 2013;47(2):182-189. doi: 10.1111/medu.12089
10. Patterson F, Cousans F, Edwards H, Rosselli A, Nicholson S, Wright B. The predictive validity of a text-based situational judgment test in undergraduate medical and dental school admissions. Acad Med. 2017;92(9):1250-1253. doi: 10.1097/ACM.0000000000001630
11. WHO. Increasing access to health workers in remote and rural areas through improved retention: Global policy recommendations. Geneva: World Health Organization; 2010. Zugänglich unter/available from: https://www.who.int/hrh/retention/guidelines/en/
12. Matsumoto M, Inoue K, Kajii E, Takeuchi K, Inoue M. Retention of physicians in rural Japan: concerted efforts of the government, prefectures, municipalities and medical schools. Rural Remote Health. 2010;10(2):1432.
13. Matsumoto M, Takeuchi K, Tanaka J, Tazuma S, Inoue K, Owaki T, Iquchi S, Maeda T. Follow-up study of the regional quota system of Japanese medical schools and prefecture scholarship programmes: a study protocol. BMJ Open. 2016;6(4):e011165. doi: 10.1136/bmjopen-2016-011165
14. Mason J. Review of Australian Government Health Workforce Programs. Canberra, Australia: Australian Government, Department of Health; 2013. Zugänglich unter/available from: https://www.health.gov.au/internet/publications/publishing.nsf/Content/work-review-australian-government-health-workforce-programs-toc
15. Frehywot S, Mullan F, Payne PW, Ross H. Compulsory service programmes for recruiting health workers in remote and rural areas: do they work? Bull World Health Organ. 2010;88(5):364-370. doi: 10.2471/BLT.09.071605
16. Reeve C, Woolley T, Ross SJ, Mohammadi L, Halili Jr SB, Cristobal F, Siega-Sur JL, Neusy AJ. The impact of socially-accountable health professional education: a systematic review of the literature. Med Teach. 2017;39(1):67-73. doi: 10.1080/0142159X.2016.1231914
17. Walker JH, Dewitt D, Pallant J, Cunningham C. Rural origin plus a rural clinical school placement is a significant predictor of medical students' intentions to practice rurally: a multi-university study. Rural Remote Health. 2012;12:1908.
18. Grobler L, Marais BJ, Mabunda S. Interventions for increasing the proportion of health professionals practising in rural and other underserved areas. Cochrane database of systematic reviews. 2015;(6):CD005314. doi: 10.1002/14651858.CD005314.pub3

Stellungnahme des Ausschusses Primärversorgung

Alle Punkte des Masterplans wurden von den Mitgliedern der Arbeitsgruppe in einem zweistufigen Delphi-Verfahren daraufhin beurteilt, ob sich der Ausschuss Primärversorgung in erster Linie für die jeweiligen Inhalte zuständig fühlt. Identifiziert wurden die Punkte 6, 12, 13, 15, 16, 17.1, 18-23, 34.1, 34.2, 35, 36.1 und 37. Zu diesen Punkten wurde eine Recherche nationaler und internationaler Literatur durchgeführt mit dem Ziel, die geplanten Maßnahmen auf wissenschaftlich belegbare Evidenz hin zu überprüfen.

Die Punkte 6, 16, 17.1 und 23 wurden unter der Fragestellung „Tragen unterschiedliche Formate mit allgemeinmedizinischen Inhalten sowie allgemeinmedizinischer Beteiligung zu einer besseren Sichtbarkeit des Faches bei?“ zusammengefasst. Die Evidenzlage zu dieser Frage ist dürftig und widersprüchlich. Das ist auch die Quintessenz eines internationalen Reviews zu dieser Fragestellung [1]. Wie widersprüchlich die Diskussionslage in Deutschland ist, zeigt sich besonders gut an einer noch aktuellen Diskussion in der Zeitschrift für Allgemeinmedizin [2], [3]. Die Frage, ob unterschiedliche Formate mit allgemeinmedizinischen Inhalten zur besseren Sichtbarkeit des Faches beitragen, wurde direkt/explizit bisher nicht untersucht. Es liegt wenig Evidenz mittelmäßiger bis niedriger Qualität vor zur Institutionalisierung der Allgemeinmedizin und ihrer Sichtbarkeit im Studium.

Es scheint aber offensichtlich - und die vorhandene Literatur stützt diese These, wenn auch auf niedrigem Niveau - dass mehr Beteiligung der Allgemeinmedizin eine bessere Sichtbarkeit mit sich bringt. Ob das zu einer besseren Einstellung gegenüber der hausärztlichen Medizin führt, ist plausibel und wahrscheinlich, aber nicht gesichert [4-8].

Der Punkt 13 des Masterplans stellt eine politische Absichtserklärung dar, aus der sich u.E. keine wissenschaftliche Fragestellung ableiten lässt. Die Punkte 15, 18 und 34.1 wurden unter der Fragestellung „Sind Maßnahmen in der Ausbildung identifizierbar, die zu einer Attraktivitätssteigerung des ländlichen Raumes führen?“ zusammengefasst. Hier finden sich zahlreiche Reviews, vornehmlich aus dem anglo-amerikanischen Raum, die, sehr unterschiedliche und inhomogene Interventionen während der studentischen Ausbildung zusammenfassend, von eher moderaten Effekten im Hinblick auf eine spätere berufliche Tätigkeit im ländlichen Raum berichten [9-11]. Je komplexer und longitudinaler solche Interventionen werden, umso höher ihr Wirkungsgrad [12]. Sehr intensive Programme (z.B. Ausbildungsstätte im ländlichen Raum, Paten-Gemeinde und strikte Ausrichtung des Curriculums auf „Community Medicine“) zeigen Wirkungsgrade von bis zu 64%. Dabei ist allerdings der Faktor ländliche Herkunft nicht herausgerechnet [13]. Für Deutschland existieren einige Einzelstudien, die letztlich eine Motivationssteigerung der Studierenden für die Allgemeinmedizin belegen können, ohne die Karrierewege nach dem Studium weiter verfolgt zu haben [4], [14], [15]. Eine weitere Studie hat zeigen können, dass Ärzte in Weiterbildung, die ein PJ-Tertial Allgemeinmedizin absolviert haben, zu 60% den Facharzt für Allgemeinmedizin anstreben. Hier ist der Faktor ländliche Herkunft allerdings von entscheidender Bedeutung [16].

Die Wirksamkeit einer „Landarztquote“ wurde bislang nicht hinreichend untersucht. Man kann allerdings an dieser Stelle die Frage stellen, ob es eine Evidenz für geeignete Auswahlkriterien für Studierende gibt, die über die Landarztquote zugelassen werden sollen. Ein Cochrane Review belegt, dass ländliche Herkunft der einzig konsistente Prädiktor für eine

spätere Tätigkeit im ländlichen Raum ist, der sich durch nahezu alle Studien hindurchzieht [17]. Zu einer ähnlichen Einschätzung kommt ein WHO-Report [18].

Die Punkte 21 und 22 des Masterplans wurden unter der Frage „Tragen die Institutionalisierung des Faches Allgemeinmedizin und der Ausbau der allgemeinmedizinischen Forschung zu einer Attraktivitätssteigerung des Faches bei?“ zusammengefasst. Es gibt Hinweise, dass eine Institutionalisierung der Allgemeinmedizin (und damit indirekt eine Stärkung der Forschungstätigkeit) für eine positive Einstellung zum Fach und zur Motivation für eine spätere hausärztliche Tätigkeit beiträgt [7].

Die Punkte 12, 19, 20, 24.2, 35 und 36.1 wurden unter der Fragestellung: „Gibt es Hinweise auf sinnvolle flankierende Maßnahmen, die die Allgemeinmedizin bzw. den ländlichen Raum im Studium attraktiver gestalten?“ Die Allgemeinmedizin wird an den Medizinischen Fakultäten in Europa in sehr unterschiedlichem Ausmaß gelehrt [19]. Die Ansätze fokussieren im Wesentlichen auf entweder eine longitudinale Verankerung im Pflichtcurriculum, eine quantitativ und qualitativ hochwertige Lehre und/oder besondere Wahlpflichtangebote [20]. Es gibt Hinweise darauf, dass die frühe und longitudinale Integration der Allgemeinmedizin die Quote allgemeinärztlich tätiger Absolventen erhöht [21], [22].

Eine entscheidende Rolle für die Prägung von Berufswünschen – im ländlichen Bereich und/oder in der Allgemeinmedizin tätig zu werden – spielen positive Rollenmodelle. Eine der Grundvorrausetzungen, um diese zu erleben, sind zeitlich passende, relevante und gut ins Curriculum integriert Praktika [9]. Die Auslagerung von Curriculumsanteilen im Sinne eines dezentralen Trainings hat einen moderaten Effekt auf die landärztliche Tätigkeit von Absolventen in Australien [11] und anderen Ländern [23]. Zu berücksichtigen ist hierbei, dass diese dezentralen Anteile sehr heterogen aufgebaut sind. Gemeinsam ist den Programmen, dass die Studierenden aktiver in die Versorgung eingebunden sind und bessere Lerneffekte bezüglich versorgungsrelevanter Skills erwerben [21]. Entscheidend sind auch hier [9] ein passender curricularer Kontext, eine gute Infrastruktur (z.B. Internetanschlüsse) und eine gute didaktische Betreuung. Die örtlichen Supervisoren müssen adäquat auf die Tätigkeit vorbereitet werden [24]. Studierende sind mit Inhalten und Lernprozessessen in allgemeinmedizinischen Praxisrotationen zufrieden. Diese ergänzen zuvor Gelerntes und ermöglichen eine besondere Lernerfahrung im Umgang mit akuten und chronischen Zuständen, Gesundheitsförderung, Prävention, Kommunikation und spezifischer Problemlösung. Die Einstellungen von Studierenden bezüglich der Allgemeinmedizin verbessert sich, aber dies bleibt nicht zwingend bis zur Berufstätigkeit erhalten [1], [21]. Zur Qualifizierung der vor Ort klinisch tätigen Ausbilder in Hausarztpraxen und Kliniken werden vorbereitende und begleitende Qualifizierungs- Maßnahmen sowohl durch die Fakultäten als auch ggf. durch die Arbeitgeber vor Ort benötigt. Hierzu zählt auch eine finanzielle Unterstützung um genügend Zeit für die Lehre / Ausbildung aufbringen zu können [25].

Beigetragen von (alphab.): Erika Baum, Klaus Böhme, Maren Ehrhardt, Folkert Fehr, Markus Gulich, Bert Huenges, Irmgard Streitlein-Böhme

Literaturverzeichnis

1. Turkeshi E, Michels NR, Hendrickx K, Remmen R. Impact of family medicine clerkships in undergraduate medical education: a systematic review. BMJ Open. 2015;5(8):e008265. doi: 10.1136/bmjopen-2015-008265

2. Rauscher C, Ernst B, Braun M, Schwindl G, Hoffmann H, Kleisch D, Salzberger B. Ausbildung für die regionale Versorgung – das Fach Allgemeinmedizin an der Universität Regensburg. Z Allg Med. 2016;92(9):352-356. doi: 10.3238/zfa.2016.0352–0356
3. Schneider A, Gensichen J, Tauscher M. Zur Notwendigkeit der akademischen Institutionalisierung des Faches Allgemeinmedizin. Z Allg Med. 2017;93(11):456-458. doi: 10.3238/zfa.2017.0456–0458
4. Böhme K, Sachs P, Niebling W, Kotterer A, Maun A. Macht das Blockpraktikum Allgemeinmedizin Lust auf den Hausarztberuf? Eine Analyse studentischer Evaluationen. Z Allg Med. 2016;92(5):220-225. doi: 10.3238/zfa.2016.0220–0225
5. Carney PA, Eliassen MS, Pipas CF, Genereaux SH, Nierenberg DW. Ambulatory care education: how do academic medical centers, affiliated residency teaching sites, and community-based practices compare? Acad Med. 2004;79(1):69-77.
6. O'Sullivan M, Martin J, Murray E. Students' perceptions of the relative advantages and disadvantages of community-based and hospital-based teaching: a qualitative study. Med Educ. 2000;34(8):648-655.
7. Schneider A, Karsch-Völk M, Rupp A, Fischer MR, Drexler H, Schelling J, Berberat P. Determinanten für eine hausärztliche Berufswahl unter Studierenden der Medizin: Eine Umfrage an drei bayerischen Medizinischen Fakultäten. GMS Z Med Ausbild. 2013;30(4):Doc45. doi: 10.3205/zma000888
8. Whitcomb ME. Ambulatory care education: what we know and what we don't. Acad Med. 2002;77(7):591-592.
9. Bunker J, Shadbolt N. Choosing general practice as a career-the influences of education and training. Aust Fam Physician. 2009;38(5):341-344.
10. Curran V, Rourke J. The role of medical education in the recruitment and retention of rural physicians. Med Teach. 2004;26(3):265-272.
11. O'Sullivan BG, McGrail MR, Russell D, Chambers H, Major L. A review of characteristics and outcomes of Australia's undergraduate medical education rural immersion programs. Hum Resour Health. 2018;16(1):8. doi: 10.1186/s12960-018-0271-2
12. Hsueh W, Wilkinson T, Bills J. What evidence-based undergraduate interventions promote rural health? N Z Med J. 2004;117(1204):U1117.
13. Rabinowitz HK, Diamond JJ, Markham FW, Wortman JR. Medical school programs to increase the rural physician supply: a systematic review and projected impact of widespread replication. Acad Med. 2008;83(3):235-243. doi: 10.1097/ACM.0b013e318163789b
14. Holst J, Normann O, Herrmann M. Strengthening training in rural practice in Germany: new approach for undergraduate medical curriculum towards sustaining rural health care. Rural Remote Health. 2015;15(4):3563.
15. Samos FA, Heise M, Fuchs S, Mittmann S, Bauer A, Klement A. Pilot phase evaluation of the elective general practice class: results of student surveys of the first two years. GMS J Med Educ. 2017;34(1)Doc4. doi: 10.3205/zma001081
16. Böhme K, Siegel A, Kotterer A, Streitlein-Böhme I, Maun A. PJ-Wahlfach Allgemeinmedizin - Eine Weichenstellung für die Hausarztkarriere. Z Allg Med. 2018;94(4):179-184.
17. Grobler L, Marais BJ, Mabunda S. Interventions for increasing the proportion of health professionals practising in rural and other underserved areas. Cochrane database of systematic reviews. 2015;(6):CD005314. doi: 10.1002/14651858.CD005314.pub3

18. Dolea C. Increasing access to health workers in remote and rural areas through improved retention: global policy recommendations. Geneva: World Health Organization; 2010.
19. Brekke M, Carelli F, Zarbailov N, Javashvili G, Wilm S, Timonen M, Tandeter H. Undergraduate medical education in general practice/family medicine throughout Europe–a descriptive study. BMC Med Educ. 2013;13(1):157. doi: 10.1186/1472-6920-13-157
20. Blozik E, Ehrhardt M, Scherer M. Förderung des allgemeinmedizinischen Nachwuchses. Bundesgesundheitsblatt Gesundheitsforschung Gesundheitsschutz. 2014;57(7):892-902.
21. Nair M, Fellmeth G. Current efforts in medical education to incorporate national health priorities. Med Educ. 2018;52(1):24-33. doi: 10.1111/medu.13395
22. Tandeter H, Granek-Catarivas M. Choosing primary care? Influences of medical school curricula on career pathways. Isr Med Assoc J. 2001;3(12):969-972.
23. Farmer J, Kenny A, McKinstry C, Huysmans RD. A scoping review of the association between rural medical education and rural practice location. Hum Resour Health. 2015;13(1):27. doi: 10.1186/s12960-015-0017-3
24. De Villiers M, Van Schalkwyk S, Blitz J, Couper I, Moodley K, Talib Z, Young T. Decentralised training for medical students: a scoping review. BMC Med Educ. 2017;17(1):196. doi: 10.1186/s12909-017-1050-9
25. von Below B, Hellquist G, Rödjer S, Gunnarsson R, Björkelund C, Wahlqvist M. Medical students' and facilitators' experiences of an Early Professional Contact course: Active and motivated students, strained facilitators. BMC Med Educ. 2008;8(1):56. doi: 10.1186/1472-6920-8-56

Stellungnahme des Ausschusses Praktische Fertigkeiten

Den Praktischen Fertigkeiten im Medizinstudium kommt im Masterplan Medizinstudium 2020 ein hoher Stellenwert zu. So steht bereits im zweiten Satz der Pressemitteilung des Bundesministeriums für Bildung und Forschung [1] dazu *„Die Lehre wird an der Vermittlung arztbezogener Fähigkeiten ausgerichtet. Dabei gilt das besondere Augenmerk dem Arzt-Patienten-Gespräch, das maßgeblich die Arzt-Patienten-Beziehung, den Behandlungserfolg und das Wohlbefinden der Patientinnen und Patienten beeinflusst.“*

Dies wird vom Ausschuss für Praktische Fertigkeiten als Auftrag gesehen, die primär am Patienten ausgeübten Fertigkeiten möglichst praxisnah zu üben und die erwähnten Fertigkeiten zunächst in simulierten Settings erwerben und dann am Patientenbett zu festigen. Im Vordergrund steht dabei insbesondere die Patientensicherheit.

Auch im Beschlusstext [1] kommt den praktischen Fertigkeiten eine hohe Bedeutung zu. Die explizite Erwähnung von OSCE als nationale Prüfungsform wird vom Ausschuss ausdrücklich begrüßt und geht mit der Konsequenz einher, dass die räumliche, sächliche und personelle Ausstattung der Skillslabs, die mittlerweile flächendeckend in den medizinischen Fakultäten vorhanden sind [2], [3], entsprechend angepasst werden muss. Dies stellt die Fakultäten sicher vor einige Herausforderungen, die nach Einschätzung des Ausschusses aber gemeistert werden können. Auch die Ankündigung, dass der NKLM [4] als verbindlicher Teil der Approbationsordnung angesehen wird, wird ausdrücklich begrüßt, da der NKLM in den Kapiteln 14b (Klinisch-praktische Fertigkeiten), 14c (Ärztliche Gesprächsführung) und 17 (Notfallmedizin) weitgehend dem Positionspapier des Ausschusses für Praktische Fertigkeiten entspricht [5]. Auch in der Zukunft wird der Ausschuss für Praktische Fertigkeiten sich an der Entwicklung des NKLM beteiligen.

Die Stärkung der kommunikativen Fertigkeiten wird vom Ausschuss begrüßt, da er nach Einschätzung des Ausschusses die Patientensicherheit erhöhen wird [6]. Auch wenn der Umfang sicher noch zu diskutieren ist (ein nationales Mustercurriculum ist zu undifferenziert), werden die praktischen Prüfungen erhebliche Anforderungen an die räumliche, sächliche und personell-qualifizierte Ausstattung bzw. Bereitstellung der Fakultäten stellen. Die Verknüpfung klinischer und theoretischer Lerninhalte ab dem ersten Studienjahr bedingt aus Sicht des Ausschusses eine Stärkung des simulationsbasierten Unterrichts in Skills Labs schon in den ersten Jahren, um die Studierenden auf den Patientenkontakt angemessen vorbereiten zu können.

Als Manko sieht es der Ausschuss an, dass die Simulationssettings, die die Patientensicherheit erhöhen, weil sie nachweislich zu besseren Ergebnissen als das konventionelle Bedsideteaching führen [7] nicht expliziter erwähnt sind. Genauer sollten diese als „Unterricht am Krankenbett“ definiert sein, um eine adäquate Dozierenden-Studierenden-Relation zu erreichen. Hier könnte eine Win-Win-Situation durch den Einbezug moderner Ausbildungsstätten mit Skills Labs ressourcensparend für die Medizinischen Fakultäten entstehen, die über die Ausbildung hinaus wirksam ist („deliberate practice“ wirkt auch in der Weiter- und Fortbildung). Zudem weist der Entwurf Masterplan Medizinstudium 2020 nicht einen Hinweis auf interprofessionelle Ausbildung aller Gesundheitsberufe auf und verkennt damit erheblich das Potential, welches insbesondere im gemeinsamen Tun – also der Ausübung patientennaher Fertigkeiten – trainiert und reflektiert werden kann.

Der Ausschuss sieht es auch als kritisch an, dass keine Übergangsfristen avisiert sind, die den Fakultäten die Möglichkeit geben könnten, sich frühzeitig auf die stark gestiegenen Anforderungen an die Skillslabs einzustellen.

Insgesamt weist der Masterplan 2020 in die richtige Richtung und wird vom APF begrüßt.

Beigetragen von (alphab.): Kai Schnabel, Christoph Stosch

Literaturverzeichnis

1. Bundesministerium für Bildung und Forschung. Masterplan Medizinstudium 2020. Berlin: Bundesministerium für Bildung und Forschung; 2017. Zugänglich unter/available from: https://www.bmbf.de/de/masterplan-medizinstudium-2020-4024.html
2. Damanakis A. Statusreport von Skills Labs in der D-A-CH-Region und Aufbau einer Informationsplattform zur Katalogisierung und Bewertung von Simulatoren zur medizinischen Ausbildung. Marburg: Philipps-Universität-Marburg; 2015.
3. Stosch C, Schnabel KP. Didactic, practical, good! 20 years of clinical skills training in the German speaking countries. GMS J Med Educ. 2016;33(4):Doc67. doi: 10.3205/zma001066
4. Hickel R, Fischer M. Stand der nationalen kompetenzbasierten Lernzielkataloge Medizin (NKLM) und Zahnmedizin (NKLZ). Halle/Berlin: Medizinischer Fakultätentag; 2013.
5. Schnabel K, Boldt PD, Breuer G, Fichtner A, Karsten G, Kujumdshiev S, Schmidts M, Stosch C. Konsensusstatement „Praktische Fertigkeiten im Medizinstudium "-ein Positionspapier des GMA-Ausschusses für praktische Fertigkeiten. GMS Z Med Ausbild. 2011;28(4):Doc58. doi: 10.3205/zma000770
6. Tamblyn R, Abrahamowicz M, Dauphinee D, Wenghofer E, Jacques A, Klass D, Smee E, Blackmore D, Winslade N, Girad N, Du Berger R, Bartman I, Buckeridge DL, Hanley JA. Physician scores on a national clinical skills examination as predictors of complaints to medical regulatory authorities. JAMA. 2007;298(9):993-1001.
7. McGaghie WC, Issenberg SB, Cohen MER, Barsuk JH, Wayne DB. Does simulation-based medical education with deliberate practice yield better results than traditional clinical education? A meta-analytic comparative review of the evidence. Acad Med. 2011;86(6):706-711. doi: 10.1097/ACM.0b013e318217e119

Stellungnahme des Ausschusses Kommunikative und soziale Kompetenzen

Der Implementierung sozialer und kommunikativer Kompetenzen in das Medizinstudium kommt im Masterplan Medizinstudium 2020 ein hoher Stellenwert zu. Der Ausschuss Kommunikative und soziale Kompetenzen der GMA begrüßt diese Entwicklung ausdrücklich, insbesondere das Ziel, dass longitudinale Kommunikationscurricula an allen Medizinischen Fakultäten Eingang finden. Neben der Arzt-Patienten-Kommunikation ist die Erweiterung kommunikativer und sozialer Kompetenzen um Aspekte der inter- und intraprofessionellen sowie der multidisziplinären und -professionellen Kommunikation zeitgemäß [1], [2].

Im Masterplan Medizinstudium 2020 wird die Umsetzung der Vorgaben basierend auf dem „Nationalen Mustercurriculum Kommunikation (LongKomm)“ [3] angestrebt. Dieses Mustercurriculum umfasst insgesamt 450 Unterrichtseinheiten (UE) (davon 350 UE als Pflichtveranstaltungen und 100 UE als Wahlpflichtveranstaltungen). Es sieht darüber hinaus vor, kommunikative Lehrinhalte auch im klinischen Unterricht zu integrieren. In der Regel umfassen die medizinischen Curricula an deutschen Fakultäten 5500 Stunden Lehre (vgl. §1(2) ÄAppO [https://www.gesetze-im-internet.de/_appro_2002/BJNR240500002.html], sowie Abschnitt 2 Artikel 24 (2)) RL 2005/36/EC, Abschnitt 2, Artikel 24 (2) [4]]. Eine Erweiterung dieses Lehrumfangs ist im Masterplan Medizinstudium 2020 nicht vorgesehen. Dies bedeutet, dass von 5500 Stunden über 8 Prozent der Pflicht- und Wahlpflichtlehre für die curriculare Vermittlung kommunikativer und sozialer Kompetenzen – ohne integrierte Veranstaltungen – veranschlagt werden. Im Vergleich zu z.B. allen Fächern der Vorklinik, die laut geltender Approbationsordnung mindestens 630 Stunden umfassen sollen, erscheint der Stundenumfang unverhältnismäßig hoch.

Der Nationale Kompetenzbasierte Lernzielkatalog Medizin [http://www.nklm.de] umfasst insgesamt 18 Kompetenz- und Lernzielbereiche und soll als Orientierung für die curriculare Gestaltung an den Fakultäten dienen. Im NKLM findet sich jedoch keine Angabe zu zeitlichen Unterrichtsumfängen der einzelnen Bereiche. Es ist nicht nachvollziehbar, warum dies nun für einen einzelnen Kompetenzbereich vorgenommen und wie dieser hohe Stundenumfang begründet wird. Eine Evidenz für diesen spezifischen Vorschlag im Masterplan Medizinstudium 2020 ist nicht gegeben.

Im Jahr 2015 hatte bereits ein Drittel aller medizinischen Fakultäten im deutschsprachigen Raum (Deutschland, Österreich, Schweiz; sog. DACH-Region) ein eigenes longitudinales Kommunikationscurriculum implementiert [6]. Zwar basieren viele Bestandteile des Mustercurriculums „LongKomm“ auf Erfahrungen und Best-Practice-Beispielen verschiedener Kommunikationscurricula an den Standorten der DACH-Region, jedoch ist eine verbindliche Vorgabe für ein Teilcurriculum für alle Fakultäten aus verschiedenen Gründen nicht sinnvoll. Zunächst finden sich im Masterplan Medizinstudium 2020 selbst Widerspruche, da einerseits ein national einheitliches Kommunikationscurriculum umgesetzt werden soll und andererseits die Fakultäten aufgefordert sind, Innovationen im Lehren und Lernen voran zu treiben. Weiterhin fällt auf, dass für diese angestrebte Implementierung der kommunikativen Lehr- und Lernziele bzw. eines longitudinalen Kommunikationscurriculums das Mustercurriculum „LongKomm“ prioritär vorgeschlagen wird. Diese Betonung wird im Masterplan Medizinstudium 2020 nicht begründet und ist daher nicht nachvollziehbar. Zum einen bestehen bereits Kommunikationscurricula, die beständig evaluiert und weiterentwickelt werden [5-8]. Zum anderen fehlen in den Ausführungen des Masterplan

Medizinstudium 2020 evidenzbasierte Gründe, um die Implementierung eines national einheitlichen Curriculums für alle Fakultäten, unabhängig von den eigenen Curriculumsinhalten und der Curriculumsgestaltung bzw. den -schwerpunkten und der Art des Curriculums (Regel- oder Modellstudiengang), nachvollziehbar zu rechtfertigen.

Darüber hinaus bleibt offen, inwieweit der Konzeptions- bzw. Entstehungsprozess des „LongKomm“ wissenschaftlich fundiert erfolgt ist oder ob es vielmehr ein Meinungsbild einer Personengruppe darstellt. Zudem lässt das „LongKomm“ selbst wissenschaftliche Evidenzen für ein qualitatives Alleinstellungsmerkmal vermissen.

Unabhängig von der Art des Kommunikationscurriculums erfordert die Implementierung bzw. der Ausbau der Lehre in diesem Bereich neben der Umstrukturierung sowohl für die Lehre als auch für die geforderten Prüfungen entsprechend qualifizierte Lehrende. Hierfür werden weder Ressourcen noch Strategien in den entsprechenden Maßnahmen thematisiert. Zudem umfasst der Masterplan Medizinstudium 2020 viele Maßnahmen, die scheinbar verschiedene Bereiche thematisieren, jedoch im Sinne eines integrierten Curriculums gemeinsam betrachtet werden sollen. So sind z.B. ambulantes Arbeiten, kommunikative und soziale Kompetenzen und Wissenschaftlichkeit keine getrennten Bereiche. Auch das Thema Prüfungen sollte nicht unabhängig vom Kontext betrachtet werden.

Wir begrüßen die deutliche Aufforderung, an allen Medizinischen Fakultäten longitudinale Kommunikationscurricula zu implementieren. Die Konzeption und Umsetzung dieser muss jedoch den jeweiligen Hochschulen überlassen werden, da diese die fachliche Expertise haben. Hier würde von Seiten der Politik ein Eingriff in die Autonomie der Hochschulen vorgenommen, der aus wissenschaftlicher Sicht nicht hinnehmbar wäre.

Elemente, die bereits an einzelnen Medizinischen Fakultäten etabliert sind, sollten nach eigenem Ermessen erhalten bleiben bzw. weiterentwickelt werden können. Daher ist insbesondere die Maßnahme der Einführung eines nationalen einheitlichen Kommunikationscurriculums an allen Medizinischen Fakultäten abzulehnen.

Beigetragen von (alphab.): Cadja Bachmann, Julia Freytag, Anja Härtl, Linn Hempel, Tanja Hitzblech, André Karger, Gudrun Karsten, Rolf Kienle, Claudia Kiessling, Tim Peters, Swetlana Philipp, Katrin Rockenbauch, Kai Schnabel, Andrea Schönbauer, Michael Sommer, Tina Stibane, Anja Zimmermann

Literaturverzeichnis

1. Bachmann C, Abramovitch H, Barbu CG, Cavaco AM, Elorza RD, Haak R, Loureiro E, Ratasjka A, Silverman J, Winterburn S, Rosenbaum M. A European consensus on learning objectives for a core communication curriculum in health care professions. Patient Educ Couns. 2013;93(1):18-26. doi: 10.1016/j.pec.2012.10.016
2. Von Fragstein M, Silverman J, Cushing A, Quilligan S, Salisbury H, Wiskin C; UK Council fo rClinical Communication Skills Teaching in Undergraduate Medical Eduation. UK consensus statement on the content of communication curricula in undergraduate medical education. Med Educ. 2008;42(11):1100-1107. doi: 10.1111/j.1365-2923.2008.03137.x

3. Jünger J, Mutschler A, Kröll K, Weiss C, Fellmer-Drügg E, Köllner V, Ringel N. Ärztliche Gesprächsführung in der medizinischen Aus-und Weiterbildung-Das Nationale longitudinale Mustercurriculum Kommunikation. Med Welt. 2015;66:189-192.
4. Europäische Union. Richtlinie 2005/36/EG des Europäischen Parlaments und des Rates vom 7. September 2005 über die Anerkennung von Berufsqualifikationen. Brüssel: Europäische Union; 2005.
5. Härtl A, Bachmann C, Blum K, Höfer S, Peters T, Preusche I,Raski B, Rüttermann S, Wagner-Menghin M, Wünsch A, Kiessling C; GMA-Ausschuss Kommunikative und Soziale Kompetenzen . Desire and reality–teaching and assessing communicative competencies in undergraduate medical education in German-speaking Europe–a survey. GMS Z Med Ausbild. 2015;32(5):Doc56. doi: 10.3205/zma000998
6. Kienle R, Arends P, Beck S, Dettmer S, Hölzer H, Sonntag U, Steinbart D, Peters H. Evaluation des longitudinalen und integrierten Curriculums der Lehrveranstaltung „Kommunikation, Interkation, Teamarbeit“. In: Jahrestagung der Gesellschaft für Medizinische Ausbildung (GMA); 25.09.-27.09.2014; Hamburg. Düsseldorf: German Medical Science GMS Publishing House; 2014. DocP433. doi: 10.3205/14gma161
7. Karger A, Hempel L. Wie angehende Ärzte medizinische Kommunikation lernen: Die Vermittlung kommunikativer Kompetenz im Medizinstudium am Beispiel von CoMeD. In: Bechmann S, ed. Sprache und Medizin: Interdisziplinäre Beiträge zur medizinischen Sprache und Kommunikation. Forum für Fachsprachen-Forschung. 138. Berlin: Frank & Timme; 2017. p.401-418.
8. Terzioglu P, Jonitz B, Schwantes U, Burger W. Kommunikative und soziale Kompetenzen-Vermittlung muss im Medizinstudium beginnen. Dtsch Ärztebl. 2003;100(36):2277-2278.

Stellungnahme des Ausschusses Simulationspatienten

Als Ausschuss begrüßen wir die im Masterplan Medizinstudium 2020 geforderte Ausweitung praktischer Anteile, wie z.B. Simulationen im Lehr- und Prüfungssektor, den frühen und kontinuierlichen Einsatz dieser Methoden sowie ihre stärkere Gewichtung in der Vermittlung von kommunikativen, sozialen und interprofessionellen Kompetenzen. Insbesondere die Stärkung der Arzt-Patient-Kommunikation halten wir ebenfalls für ausgesprochen wichtig.

Solche Kompetenzen können nur unzureichend rein theoretisch gelehrt und schriftlich geprüft werden. Sie bedürfen einer Unterfütterung durch praktischen Unterricht und praktische Prüfungen. Als effektive Methode hat sich dabei der Einsatz von Simulationspatienten (SP) bewährt. Auch für praxisorientierte Auswahlverfahren als zusätzliche Kriterien neben der Abiturnote sind Verfahren unter Einbezug von SPs denkbar und an einigen Standorten bereits in Erprobung.

Die Fakultäten haben in den letzten Jahren bereits SP-Programme etabliert, die die Vermittlung der geforderten Kompetenzen unterstützen, indem sie beispielsweise die praxisorientierte Ausbildung in Kleingruppen ausgebaut haben. Über das Training kommunikativer Kompetenzen hinaus werden mit SPs auch weitere praktische Fertigkeiten und Haltungen früh und kontinuierlich im Laufe der Curricula trainiert. Diese Bemühungen stehen im Einklang mit den Zielen des Masterplan Medizinstudium 2020.

Abgesehen von der inhaltlichen Befürwortung, halten wir es jedoch für unabdingbar, den einzelnen Fakultäten hierbei Gestaltungsmöglichkeiten zu überlassen, um ein Longitudinales Kommunikations-Curriculum dem jeweiligen Standort und dem dort bestehenden Curriculum anpassen zu können.

Durch die mit dem Masterplan Medizinstudium 2020 angestrebten Entwicklungen wird die Methode des Einsatzes von SPs an Bedeutung in Lehre und Prüfung gewinnen.

Wenn in den verschiedenen oben genannten Formaten SPs eingesetzt werden, dann müssen dabei die Qualitätskriterien eingehalten werden, die sowohl national [1] als auch international [2], [3] formuliert wurden. Hinter diese Standards darf gerade bei einer massiven Erweiterung von SP-Einsätzen und deren Integration in high-stakes-Prüfungen nicht zurückgefallen werden; im Gegenteil gilt es, sie mit Leben zu füllen und konstruktiv weiterzuentwickeln. Bezüglich der Einführung von OSCEs als Teile der Staatsexamina sind klare Standards für die Durchführung – unter anderem für die Konzeption von Prüfungsstationen und die Qualifizierung der Prüferinnen und Prüfer – unabdingbar. Wir fordern in diesem Zusammenhang, dass bei der Entwicklung dieser Standards die Fakultäten einbezogen werden und die dort bereits vorhandene Expertise zur Durchführung von OSCEs genutzt wird.

Auch in etwaigen Auswahlverfahren mithilfe von Simulationen müssen die gleichen hohen Standards wie bei Prüfungen gelten - mit den entsprechenden Konsequenzen für Ressourcen und Qualitätssicherung.

Die Fakultäten haben beim Einsatz von SPs eine große Expertise entwickelt und Erfahrungen gesammelt. Diese gilt es gezielt weiterzuentwickeln und zu fördern. Die wissenschaftliche Erforschung der Methode muss weiter vorangebracht werden. Gerade der angestrebte Einsatz von SPs in der Ärztlichen Prüfung stellt einen Entwicklungssprung dar. Die Anforderungen an die Qualität der Darstellung z.B. in Bezug auf Standardisierung müssen der Bedeutung der Ärztlichen Prüfung für den Berufsstart gerecht werden. Die Bedingungen

unter denen dies im deutschen Ausbildungssystem mit einer so großen Zahl von SPs, die nicht gemeinsam trainiert werden können, gelingen kann, müssen erst noch erforscht werden.

Die im Masterplan geforderte Ausweitung der Lehre in diesem Bereich und die zusätzlichen Prüfungen stellen einen Aufgabenzuwachs dar, der mit den vorhanden personellen und materiellen Ressourcen nicht bewältigt werden kann. Daher müssen die Fakultäten mit den entsprechenden finanziellen Mitteln ausgestattet werden, um dies zu leisten. Dies beinhaltet Mittel für:

- Eine angemessene Vergütung einer ausreichenden Zahl von SPs,
- Didaktisch qualifiziertes Personal für die Rekrutierung, Schulung der SPs sowie Lehr- und Prüfungspersonal,
- Personal für die Entwicklung longitudinaler Curricula,
- Lehrbegleitforschung,
- Fortlaufende Qualifizierungsmaßnahmen,
- Räumliche und weitere materielle Ressourcen.

Beigetragen von (alphab.): Anke Adelt, Julia Freytag, Angelika Hiroko Fritz, Tim Peters, Renate Strohmer, Christian Thrien

Literaturverzeichnis

1. Peter T, Thrien C. Simulationspatienten: Handbuch für die Aus- und Weiterbildung in medizinischen und Gesundheitsberufen. Bern: Hogrefe; 2018.
2. Cleland JA, Abe K, Rethans JJ. The use of simulated patients in medical education: AMEE Guide No 42. Med Teach. 2009;31(6):477-486.
3. Lewis KL, Bohnert CA, Gammon WL, Hölzer H, Lyman L, Smith C, Thompson TM, Wallace A, Gilva-McConvey G. The association of standardized patient educators (ASPE) standards of best practice (SOBP). Adv Simul (London). 2017;2(1):10. doi: 10.1186/s41077-017-0043-4

Stellungnahme des Ausschusses Interprofessionelle Ausbildung

Der Ausschuss für interprofessionelle Ausbildung begrüsst grundsätzlich den Masterplan Medizinstudium 2020. Die zukünftige PatientInnenversorgung wird mehr und mehr als Teamarbeit verschiedener Gesundheitsberufe erfolgen müssen, und auf diese notwendige Zusammenarbeit muss das Medizinstudium besser vorbereiten. Die Evidenz interprofessioneller Ausbildung auf die spätere Zusammenarbeit ist belegt [1]. Die Zusammenarbeit mit anderen Gesundheitsberufen wird im Masterplan Medizinstudium 2020 zumindest auf den Seiten 3, 4 und 5 erwähnt, der Terminus „multiprofessionell" findet sich (S. 4), „interprofessionell" fehlt jedoch.

Somit wird interprofessionelles Arbeiten nicht als notwendiger Bestandteil des „neuen" Curriculums benannt, sondern eher umschrieben. Der Ausschuss für Interprofessionelle Ausbildung befürchtet, dass der Aspekt der Zusammenarbeit und des gemeinsamen Lernens mit anderen Gesundheitsberufen somit nicht explizit genug betrachtet wird. Reeves et al. [1] definieren interprofessionelle Ausbildung: „*…as an intervention where the members of more than one health or social care profession, or both, learn interactively together, for the explicit purpose of improving interprofessional collaboration or the health/well being of patients/clients, or both.*" Zur besseren Darstellung sollte sich der Masterplan Medizinstudium 2020 auf „interprofessionelle Ausbildung" beziehen.

Um die im Masterplan Medizinstudium 2020 formulierte „multiprofessionelle Arbeit in Teams […] in der gemeinsamen Ausbildung" umsetzen zu können [2] sind sowohl interprofessionell kompetente Ärztinnen und Ärzte als Lehrende, aber auch Lehrende (und Auszubildende) anderer Gesundheitsberufe in Pflege, Therapie und Diagnostik gefordert. Dazu müssen interprofessionelle didaktische Aus- und Weiterbildungsformate geschaffen werden.

Um Nachhaltigkeit zu erzeugen, müssen im Sinne des *constructive alignments* auch interprofessionelle Prüfungen für Studierende (wie z. B. Team- OSCE [3]) etabliert werden. Im Sinne der Kompetenzorientierung müssten dafür zunächst interprofessionelle Kompetenzen formuliert werden. Hier bietet sich der Zugriff auf Kompetenzrahmen an.

Grundsätzlich bieten sich interprofessionelle Lehrformate auch für fast alle genannten Inhalte des Masterplan Medizinstudium 2020 an: kommunikative Kompetenzen, wissenschaftliche Kompetenzen, aber auch die Themen Grundversorgung und Digitalisierung.

Die Ausbildungen von Medizinstudierenden aber auch von anderen Gesundheitsfachberufen sollten zukünftig stets auch interprofessionelle Curricula beinhalten, d.h. eine longitudinale Dimension aufweisen. Ähnlich wie der Ausschuss Kommunikative und soziale Kompetenzen empfiehlt, sollte es kein deutschlandweites Mustercurriculum geben, sondern es sollte eher an den Erfahrungen der medizinischen Fakultäten mit interprofessionellen Modulen angedockt werden (vgl. z. B. https://www.bosch-stiftung.de/de/projekt/operation-team-interprofessionelles-lernen).

Letztlich muss die Kapazitätsverordnung nicht-ärztliche Lehrkräfte im Pflichtcurriculum ermöglichen, ohne die Aufnahmekapazitäten der jeweiligen Fakultät zu beeinflussen. Nur so können verbindliche (Wahl-)Pflichtveranstaltungen in die Curricula implementiert werden. Interprofessionelle Ausbildungsstationen im praktischen Ausbildungsabschnitt umgehen

diese Thematik, müssen aber im Sinne eines Curriculums mit theoretischen Lehrveranstaltungen verknüpft sein.

Konkrete Forderungen des Ausschusses für Interprofessionelle Ausbildung sind:

- Interprofessionelle Ausbildung als Lernen voneinander, miteinander und übereinander zu verstehen
- Verbindliche, möglichst verpflichtende und longitudinale Integration von Interprofessionalität ins medizinische Curriculum
- Ausloten von Modellen für die erfolgreiche Integration interprofessioneller Lehrveranstaltungen in die Curricula der beteiligten Gesundheitsberufe
- Entwicklung von Aus- und Weiterbildungen für interprofessionell Lehrende
- Definition interprofessioneller Kompetenzen unter Verwendung von Kompetenzrahmen
- Entwicklung von Prüfungen interprofessioneller Kompetenzen
- Entwicklung interprofessioneller Evaluationsformaten
- Qualitätssicherung und -entwicklung der PatientInnenversorgung steht im Mittelpunkt aller interprofessionellen Ausbildungen.

Beigetragen von (alphab.): Sebastian Bode, Marion Huber, Sylvia Kaap-Fröhlich, Birgit Wershofen

Literaturverzeichnis

1. Reeves S, Perrier L, Goldman J, Freeth D, Zwarenstein M. Interprofessional education: effects on professional practice and healthcare outcomes (update). Cochrane Database Syst Rev. 2013;3(3):CD002213. doi: 10.1002/14651858.CD002213.pub3
2. Bundesministerium für Bildung und Forschung. Masterplan Medizinstudium 2020. Berlin: Bundesministerium für Bildung und Forschung; 2017. Zugänglich unter/available from: https://www.bmbf.de/files/2017-03-31_Masterplan%20Beschlusstext.pdf
3. Singleton A, Smith F, Harris T, Ross-Harper R, Hilton S. An evaluation of the team objective structured clinical examination (TOSCE). Med Educ. 1999;33(1):34-41.

Stellungnahme des Ausschusses Integrative Medizin und Perspektivenpluralismus

Der Ausschuss Integrative Medizin und Perspektivenpluralismus begrüßt und befürwortet die im Masterplan Medizinstudium 2020 entwickelte frühzeitige und konsequente Orientierung des Studiums der Humanmedizin an Patienten und ihren Bedürfnissen ausdrücklich. Im Folgenden werden Schwerpunktsetzungen zur Gestaltung des Masterplans entwickelt und begründet; abschließend werden konkrete Maßnahmen aufgelistet.

Die Schwerpunktsetzungen betreffen

- Anthropologische Konzepte und Auseinandersetzung mit verschiedenen Modellen zu Gesundheit und Krankheitsverarbeitung
- Früh im Studium beginnende, didaktisch strukturierte Begegnungen mit Patienten und aktive Partizipation in der Patientenversorgung
- Berücksichtigung der grundlegenden Prinzipien einer Evidenz-basierten Medizin incl. der Anliegen der Patienten
- Integrative Medizin und Auseinandersetzung mit der Perspektivenpluralität vorhandener Ansätze in Wissenschaft und Gesundheitsversorgung
- Ärztliche Selbstfürsorge und -regulation als Bestandteil professioneller Persönlichkeitsentwicklung.

Anthropologische Konzepte und Auseinandersetzung mit verschiedenen Modellen zu Gesundheit und Krankheitsverarbeitung

Für ein umfassendes Verständnis von Patienten und ihren Bedürfnissen ist die Auseinandersetzung mit und Diskussion von anthropologischen Grundkonzepten im Medizinstudium erforderlich [1]. Ohne dies droht die Gefahr einer unbewussten und unreflektierten Zugrundelegung eines antiquierten mechanistischen Erklärungsmodells des Menschen, das sich aus einem reduktionistischen Wissenschaftsparadigma speist. In diesem Zusammenhang ist zudem eine intensivierte Auseinandersetzung mit verschiedenen Modellen von Gesundheit (vgl. u.a. [2], [3]) sowie Krankheitsverarbeitung (z.B. [4]) als Grundlegung für ein vertieftes Verstehen der Genese von Gesundheit und Krankheit sowie des Befindens und der Sicht des Patienten zu empfehlen.

Früh im Studium beginnende, didaktisch strukturierte Begegnungen mit Patienten und aktive Partizipation in der Patientenversorgung

Ärztliche Praxis, diagnostisches Erkennen und therapeutisches Handeln nehmen ihren Ausgang vom Hilfeersuchen individueller Personen, die gesundheitsbedingt in eine Notsituation geraten sind [5]. Um hierzu qualifiziert auszubilden, sind insbesondere didaktisch gut gestaltete Situationen resp. Lernumgebungen longitudinal im Studienverlauf zu schaffen, in denen Studierende realen und Simulationspatienten begegnen und deren komplexe Krankheits- und mehrdimensionale Lebenssituation sowie individuelle Bedürfnislage, einschließlich Sinnsuche, mit genügend Zeit erfassen lernen.

Dies kann und sollte bereits früh im Studium beispielsweise in geführten Interviews oder Explorationen von Patienten zu deren Erlebnissen im Gesundheitswesen, in z.B. Falltagen mit realen Patienten [6] oder in ähnlich konzipierten Veranstaltungen mit Simulationspatienten erfolgen. Mit zunehmendem Studienverlauf erhält die aktive Teilhabe respektive supportierte Mitarbeit von Studierenden bis hin zur eigenständigen

Patientenversorgung durch Studierende einen wichtigen Stellenwert [7]. Blockpraktika und Praktisches Jahr sind entsprechend didaktisch gut zu strukturieren und mit Reflexionsangeboten auszustatten [8]. Als Modell zur entsprechenden Umsetzung im PJ wurden Ausbildungsstationen entwickelt: Studierende werden in das klinische, multiprofessionelle Versorgungsteam integriert; sie übernehmen als studentisches Team sämtliche ärztliche Aufgaben auf der Station, werden von Lehrärzten supervidiert und unterrichtet und erhalten Support seitens des multiprofessionellen Teams [9]. Zudem werden sie durch geführte Reflexion im Rahmen eines klinischen Reflexionstrainings in ihrer professionellen Entwicklung unterstützt [10]. In einem solchen Setting tragen die Studierenden zu einer hohen Patientenorientierung der Patientenversorgung bei [11].

Mit einer sich durch das gesamte Studium erstreckenden systematischen Ausbildung können Entrustable Professional Activities (EPAs) stufenweise eingeübt und überprüft werden (vgl. [12], [13]); die Kompetenzen für die Gestaltung einer vertrauensbildenden Patienten-Arzt-Beziehung können solchermaßen intensiviert vermittelt werden.

Vollständige Berücksichtigung der Prinzipien einer Evidenz-basierten Medizin incl. der Anliegen der Patienten und Befähigung zum wissenschaftlichen Arbeiten

Die Begegnung mit Patienten in geeigneten Settings bietet ein praktisches Lern- und Anwendungsfeld auch für das „Grundlagenwissen über (…) die geistig-seelischen Eigenschaften des Menschen“ (§ 1 ÄApprO, Abs. 1, Satz 4) bzw. dessen individuelle Konkretisierung. Dies kann in seiner Bedeutung für das Erlernen und spätere Realisieren einer Evidenz-basierten Medizin (EBM) kaum hoch genug eingeschätzt werden. Hierbei ist darauf zu achten, dass bei Vermittlung von EBM nicht nur die externe Evidenz gewürdigt wird. Die Praxis der EBM ist definiert als die individuelle klinische Expertise *inklusive* der sorgfältigen Identifikation und anteilnehmenden Berücksichtigung der individuellen Notlage, Rechte und Präferenzen des Patienten, welche für die klinische Urteilsbildung in Diagnostik und Therapie in Verknüpfung mit der besten verfügbaren externen klinischen Evidenz aus systematischer Forschung zu bringen ist [14].

Anderenfalls droht medizinischer Ausbildung und Praxis eine Vereinseitigung mit Schwerpunktbildung auf Algorithmen, während die in der klinischen Praxis notwendige Kontextualisierung und Individualisierung zu kurz kommen [15]. Dies ist von immenser Bedeutung, da Deindividualisierung und Beeinträchtigungen beim Eintreten sowie Fürsprache für Patienten („impaired patient agency“) als Bestandteile einer in der medizinischen Praxis beobachtbaren Dehumanisierung beschrieben werden [16].

Integrative Medizin und Auseinandersetzung mit der Perspektivenpluralität vorhandener Ansätze in Wissenschaft und Gesundheitsversorgung

Gerade im Hinblick auf Patientenpräferenzen und die adäquate Auswahl diagnostischer und therapeutischer Schritte, aber auch zur Stärkung des „routinierten Umgang[s] mit wissenschaftlichen Methoden und Konzepten“ (Masterplan Medizinstudium 2020, Abs. 4) kann die Einbindung der Integrativen Medizin in die Primärausbildung zur Ärztin/zum Arzt entscheidende Beiträge leisten. Integrative Medizin wird verstanden als „die Praxis der Medizin, welche die Bedeutung der Beziehung zwischen Arzt und Patienten betont. Sie konzentriert sich auf die ganze Person und ist durch Evidenz informiert. Sie nutzt alle angemessenen therapeutischen und lebensstilbezogenen Möglichkeiten und angemessene Kollaboration aller Gesundheitsberufe und -disziplinen, um optimale Gesundheit und Heilung zu erreichen.“ [https://imconsortium.org/about/introduction/]. Zudem nutzt

Integrative Medizin die reiche Vielfalt therapeutischer Systeme mit konventionellen und komplementären Herangehensweisen [17], [18]. Diese Auseinandersetzung ist einerseits eine wichtige und prinzipielle Vorbereitung für den akademischen Diskurs, indem methodisch die Frage nach dem wechselseitigen Ergänzungspotential sowie dem gegenseitigen Ausschluss der unterschiedlichen medizinischen Ansätze zu verfolgen ist [19], [20]. Andererseits bereitet sie auf die alltägliche Versorgungsrealität bei hoher nationaler und internationaler Inanspruchnahme von Komplementärmedizin durch Patienten bzw. Anwendung in der ambulanten Versorgung vor [21-23]. Weiterhin fördert sie den respektvollen Dialog innerhalb der Ärzteschaft im Sinne des Medizinpluralismus (vgl. [24]).

Studierende fordern eine Auseinandersetzung mit komplementärmedizinischen Verfahren; die theoretische Vermittlung solcher Lehrinhalte, kombiniert mit praktischen Erfahrungen „am eigenen Leib" kann zur kritischen Reflexion und zur Entwicklung einer ganzheitlichen, patientenorientierten Haltung unter Medizinstudierenden beitragen [25]. Ein weiteres, erfahrungsbasiertes Unterrichtsmodell mit der Zielsetzung der metakognitiven Auseinandersetzung mit verschiedenen medizinischen Systemen, deren Grundannahmen und Herangehensweisen wurde seitens des Ausschusses entwickelt [26]. Die Förderung einer eigenen Urteilsbildung ist gerade vor dem Hintergrund der o.g. hohen Versorgungsrelevanz, Berücksichtigung der Patientenpräferenz, Beratungsbedarfs und Diskussion zur EBM von Relevanz.

Ärztliche Selbstfürsorge und -regulation als Bestandteil professioneller Persönlichkeitsentwicklung

Um den sich im 21. Jahrhundert wandelnden An- und Herausforderungen des ärztlichen Berufs und damit der medizinischen Ausbildung [27] begegnen zu können, scheint eine frühe Förderung von Selbstfürsorge und -regulation bei Studierenden indiziert. Dies entspricht auch dem neu in das ärztliche Gelöbnis resp. der Genfer Deklaration des Weltärztebundes aufgenommen Passus: „Ich werde auf meine eigene Gesundheit, mein Wohlergehen und meine Fähigkeiten achten, um eine Behandlung auf höchstem Niveau leisten zu können" [28].

Zur Konkretisierung und Umsetzung der obigen Gesichtspunkte werden folgende Maßnahmen vorgeschlagen:

- Stärkere Orientierung an mehrdimensionalen anthropologischen Modellen und Thematisierung der verschiedenen, auch sozio-kulturell-historisch bedingten, Konzepte von und Perspektiven auf Gesundheit, Krankheit und Heilung.
- Schaffung strukturierter Lernumgebungen zum Erwerb von Kompetenzen resp. vertrauenswürdigen professionellen Aktivitäten (EPAs) hinsichtlich/unter Nutzung
    a. patientenzentrierter Beziehungsgestaltung und Kommunikation unter Berücksichtigung aller (einschließlich der komplementären) Diagnose- und Behandlungsoptionen und sowie Möglichkeiten der Selbstfürsorge (self-care) sowie Lebensstil-Veränderungen.
    b. umfassender Ausbildung der Anamnese- und Untersuchungstechniken auf den Grundlagen der integrativen Versorgungsansätze.
    c. früher und longitudinaler Einbindung von Real- und Simulationspatienten in die praktische, ambulante und klinisch-praktische Lehre unter Supervision, Feedback und Reflexion ihrer psychosozialen und individuellen Bedürfnisse, beginnend im ersten Studienjahr und über den gesamten Studienverlauf.

d. früher und longitudinaler Einbindung interprofessioneller Settings in die Patientenbegegnungen, Anamnese und Therapieplanung unter Orientierung am Therapieziel des Patienten und dessen Ressourcen.

- Vermittlung von Fähigkeiten zur strukturierten Förderung der Patienten-Selbstfürsorge (self-care) und Salutogenese (ressourcenintegrierend), sowie der Selbstfürsorge und -regulation der Vermittlung von Fähigkeiten zur strukturierten Förderung der Selbstfürsorge und -regulation der Studierenden
- Verbindliche Vermittlung von Basiswissen und Förderung eigenständiger, kritischer Urteilsbildung zur Integrativen Medizin, unter Berücksichtigung und Auseinandersetzung mit verschiedenen medizinischen Systemen wie Traditioneller Chinesischer Medizin, Homöopathischer Medizin, Ayurvedischer Medizin, Anthroposophischer Medizin, ausgewählten komplementärmedizinischen Verfahren sowie klassischen Naturheilverfahren, incl. stärkerer Thematisierung der Auswirkungen von Placebo- und Noceboeffekten im Bereich der konventionellen und integrativen/komplementären medizinischen Versorgung.
- Curricular angebotene Praktika in integrativmedizinischen Einrichtungen (Kliniken, Instituten, Lehrpraxen, etc.), um die Ausübung von Perspektivenpluralismus im Praxisalltag erleben und erfahren zu können.

Beigetragen von (alphab.): Angelika Homberg, Stefanie Joos, Stefanie Merse, Beate Stock-Schröer, Christian Scheffer, Diethard Tauschel, Jan Valentini

Literaturverzeichnis

1. Heusser P, Scheffer C, Neumann M, Tauschel D, Edelhäuser F. Towards non-reductionistic medical anthropology, medical education and practitioner–patient-interaction: The example of Anthroposophic Medicine. Patient Educ Couns. 2012;89(3):455-460. doi: 10.1016/j.pec.2012.01.004
2. Huber M, Knottnerus JA, Green L, van der Horst H, Jadad AR, Kromhout D, Leonard B, Lorig K, Loureiro MI, van der Meer JW, Schnabel P, Smith R, van Weel C, Smid H. How should we define health? BMJ. 2011;343:d4163. doi: 10.1136/bmj.d4163
3. Witt CM, Chiaramonte D, Berman S, Chesney MA, Kaplan GA, Stange KC, Woolf SH, Berman BM. Defining health in a comprehensive context: A new definition of integrative health. Am J Prev Med. 2017;53(1):134-137. doi: 10.1016/j.amepre.2016.11.029
4. Leventhal H, Leventhal EA, Cameron L. Representations, procedures, and affect in illness self-regulation: A perceptual-cognitive model. In: Baum A, Revenson TA, Singer JE, eds. Handbook of health psychology. Mahwah: Lawrence Erlbaum; 2001. p.19-47
5. Matthiessen PF. Einzelfallforschung zwischen Evidence based Medicine und Narrative based Medicine. 11 Internationaler Coethener Erfahrungsaustausch (ICE 11). Köthen (Anhalt): WissHom; 2011.
6. Tauschel D, Scheffer C, Balzereit S, Hofmann M, Edelhäuser F. How can clinical thinking be taught and trained practically? The concept of Real Patient Study Days. AMEE Conference; Trondheim: Association for Medical Education in Europe (AMEE); 2007. p.116
7. Steven K, Wenger E, Boshuizen H, Scherpbier A, Dornan T. How clerkship students learn from real patients in practice settings. Acad Med. 2014;89(3):469-476. doi: 10.1097/ACM.0000000000000129
8. Sandars J. The use of reflection in medical education: AMEE Guide No. 44. Med Teach. 2009;31(8):685-695.

9. Scheffer C, Tauschel D, Cysarz D, Hahn E, Längler A, Riechmann M, Edelhäuser F. Lernen durch aktive Partizipation in der klinischen Patientenversorgung-Machbarkeitsstudie einer internistischen PJ-Ausbildungsstation. GMS Z Med Ausbild. 2009;26(3):Doc31. doi:10.3205/zma000623
10. Lutz G, Scheffer C, Edelhaeuser F, Tauschel D, Neumann M. A reflective practice intervention for professional development, reduced stress and improved patient care—A qualitative developmental evaluation. Patient Educ Couns. 2013;92(3):337-345. doi: 10.1016/j.pec.2013.03.020
11. Scheffer C, Valk-Draad MP, Tauschel D, Büssing A, Humbroich K, Längler A, Zuzak T, Köster W, Edelhäuser F, Lutz G. Students with an autonomous role in hospital care–patients perceptions. Med Teach. 2018;40(9):944-952. doi: 10.1080/0142159X.2017.1418504
.12. Peters H, Holzhausen Y, Boscardin C, ten Cate O, Chen HC. Twelve tips for the implementation of EPAs for assessment and entrustment decisions. Med Teach. 2017;39(8):802-807. doi: 10.1080/0142159X.2017.1331031
13. ten Cate O, Chen HC, Hoff RG, Peters H, Bok H, van der Schaaf M. Curriculum development for the workplace using entrustable professional activities (EPAs): AMEE guide no. 99. Med Teach. 2015;37(11):983-1002. doi: 10.3109/0142159X.2015.1060308
14. Sackett DL, Rosenberg WM, Gray JM, Haynes RB, Richardson WS. Evidence based medicine: what it is and what it isn't. 1996. Clin Orthop Relat Res. 2007;455:3-5.
15. Greenhalgh T, Howick J, Maskrey N. Evidence based medicine: a movement in crisis? BMJ. 2014;348:g3725. doi: 10.1136/bmj.g3725
16. Haque OS, Waytz A. Dehumanization in medicine: Causes, solutions, and functions. Perspect Psychol Sci. 2012;7(2):176-186. doi: 10.1177/1745691611429706
17. Briggs JP. 14e: Complementary, Alternative, and Integrative Health Practices. In: Kasper D, Fauci A, Hauser S, Longo D, Jameson JL, Loscalzo J, eds. Harrison's Principles of Internal Medicine; 2017.
18. Kligler B, Maizes V, Schachter S, Park CM, Gaudet T, Benn R, Lee R, Remen RN; Education Working Group, Consortium of Academic Health Centers for Integrative Medicine. Core competencies in integrative medicine for medical school curricula: a proposal. Acad Med. 2004;79(6):521-531.
19. Matthiessen PF. Paradigmenpluralität und Individualmedizin. In: Matthiessen PF, ed. enorientierung und Professionalität Festschrift 10 Jahre Dialogforum Pluralismus in der Medizin. 2. erweiterte Auflage ed. Bad Homburg: Verlag Akademische Schriften (VAS); 2011.
20. Matthiessen PF. 10 Jahre Dialogforum Pluralismus in der Medizin. Warum es uns gibt, wer wir sind und was wir wollen. In: Matthiessen PF, ed. enorientierung und Professionalität Festschrift 10 Jahre Dialogforum Pluralismus in der Medizin. 2. erweiterte Auflage ed. Bad Homburg: Verlag Akademische Schriften (VAS); 2011.
21. Horneber M, Bueschel G, Dennert G, Less D, Ritter E, Zwahlen M. How many cancer patients use complementary and alternative medicine: a systematic review and metaanalysis. Integr Cancer Ther. 2012;11(3):187-203. doi: 10.1177/1534735411423920
22. Linde K, Alscher A, Friedrichs C, Joos S, Schneider A. Die Verwendung von Naturheilverfahren, komplementären und alternativen Therapien in Deutschland-eine systematische Übersicht bundesweiter Erhebungen. Compl Med Res. 2014;21(2):111-8.
23. Barnes PM, Bloom B, Nahin RL. Complementary and alternative medicine use among adults and children; United States, 2007. Nat Health Stat Report. 2008;12(December 10).

24. Kiene H, Heimpel H. Ärztliche Professionalität und Komplementärmedizin: Was ist seriöse ärztliche Therapie? Dtsch Arztebl. 2010;107(12):477.
25. Valentini J, Glassen K, Eicher C, Washington-Dorando P, Weinschenk S, Musselmann B, Steinhäuser J, Joos S. „Kritische Diskussion sollte mehr gefördert werden!"–Eine qualitative Auswertung der Lehrevaluation von Medizinstudierenden zur komplementärmedizinischen Lehre. Dtsch Med Wochenschr. 2018;143(14):e125-e130. doi: 0.1055/a-0575-6851
26. Tauschel D, Edelhäuser F, Scheffer C. How can approaches of different medical systems be made visible, brought into dialogue, and reflected? - Real Patient Study Days Integrative Medicine. AMEE Conference; Basel: Association for Medical Education in Europe (AMEE); 2018. p.929
27. Frenk J, Chen L, Bhutta Z, Cohen J, Crisp N, Evens T, Fineberg H, Garcia P, Ke Y, Kelly P, Kistnasamy B, Meleis A, Naylor D, Pablos-Mendez A, Reddy S, Scrimshaw S, Sepulveda J, Serwadda D, Zurayk H. Health professionals for a new century: transforming education to strengthen health systems in an interdependent world. Lancet. 2011;376(9756):1923-1958. doi: 10.1016/S0140-6736(10)61854-5
28. World Medical Association. WMA Declaration of Geneva. The Physician's Pledge 2017 Geneva: World Medical Association; 2018. Zugänglich unter/available from: https://www.wma.net/policies-post/wma-declaration-of-geneva/

Stellungnahme des Ausschusses Gender, Diversity und Karriereentwicklung in der medizinischen Aus- und Weiterbildung

Der Ausschuss begrüßt den vorliegenden Masterplan Medizinstudium 2020 mit dem Ziel einer kompetenzorientierten, wissenschaftlichen und praxisnahen Ausbildung, der Stärkung der Allgemeinmedizin, der konsequenten Orientierung an den Patientinnen und Patienten und deren Bedürfnisse sowie der Integration kommunikativer Kompetenzen.

Die bestehende und zunehmende Diversität in unserer Bevölkerung und somit auch in der Patientenschaft und unter Studierenden machen es zunehmend notwendig, dass Diversitäts- und Genderaspekte ausreichend in der Aus- und Weiterbildung in der Medizin und in den Gesundheitsprofessionen berücksichtigt werden. Nach dem Allgemeinen Gleichbehandlungsgesetz (AGG) umfasst Diversität sechs Kategorien: Alter, Geschlecht, Ethnie, körperliche Beeinträchtigung, sexuelle Orientierung und Religion [https://dejure.org/gesetze/AGG]. Darüber hinaus spielen aber auch viele andere Aspekte eine Rolle, wenn es um Inklusion oder Diskriminierung geht (z.B. soziale Zugehörigkeit, Bildungshistorie, individuelle körperliche, funktionelle und geistige Merkmalsausprägungen bis hin zu Haarfarbe und Körperform, Leistungsfähigkeit, Aussprache, um einige wenige zu nennen).

Der Ausschuss betrachtet es daher als notwendig, dass Diversitäts-, insbesondere Genderkompetenzen, umfassender als bislang in das Curriculum mitaufgenommen werden, insbesondere da im medizinischen Bereich der Umgang mit Diversität explizit Teil der beruflichen Aufgaben darstellt. Wir verweisen hier auf das Positionspapier des Ständigen Ausschusses der Europäischen Ärztinnen und Ärzte (CPME: Comité Permanent des Médecins Européens) von 2016, das die Bedeutung der curricularen Integration der Kategorie Geschlecht betont [1]. Neben der inhaltlichen Integration in die Curricula, sollten Diversitätsaspekte auch in der Hochschuldidaktik Berücksichtigung finden [2].

Im Jahr 2013 hat der Ausschuss 207 diversitäts- und genderbezogene Lernziele für den Nationalen Kompetenzbasierten Lernzielkatalog Medizin (NKLM) vorgeschlagen, davon wurden 82 übernommen, das entspricht 4% der Gesamtzahl der Lernziele im NKLM [3]. Dem Ausschuss ist es wichtig, dass diese Lernziele langfristig nicht nur im NKLM verankert bleiben, sondern auch Eingang in die die Gegenstandskataloge bzw. die Ärztliche Prüfung finden. Insbesondere auch im Sinne der Patienten/innenorientierung und der individualisierten Medizin sind gendermedizinische und gendersensible Lehrinhalte unerlässlich. Mittelfristig ist anzustreben, auch andere Diversitäts-relevante Lernziele zu diskutieren und nachhaltig in die Medizinische Ausbildung einzubetten.

Im Bereich der wissenschaftlichen Kompetenzen sieht der Ausschuss ebenfalls dringenden Bedarf, Diversitäts- und Genderaspekte zu integrieren, so dass bereits in der Planung von (medizinischen) Studien und in den Studienkohorten beide Geschlechter vertreten sind und Studienergebnisse geschlechterspezifisch und diversitätssensibel ausgewertet werden. Wir verweisen hier auf die Veröffentlichung der National Institutes of Health, das die Integration von geschlechtsspezifischen Aspekten in die Forschung fordert [4].

Den Studierenden sollten zudem früh Informationen über Möglichkeiten der Karriereentwicklung für Ärztinnen und Ärzten geboten werden. Die Vereinbarkeit von Studium und Familie sollte gegeben sein. Aufgrund der steigenden Zahl von Studentinnen und der zukünftig höheren Anzahl von Ärztinnen sollten Teilzeitarbeits- sowie Teilzeit-

Studienmodelle ermöglicht werden. Es sollte dabei aber auch erleichtert werden, trotz Familiengründung Vollzeit zu arbeiten/zu studieren bzw. Karrierenachteile durch Teilzeittätigkeit zu verhindern/abzubauen.

Diskriminierungsfreie Prüfungen, die der Diversität der Studierenden gerecht werden, hält der Ausschuss für obligat und essenziell. Weder bei den Prüfungen noch bei der Zulassung zum Studium dürfen Diskriminierungen nach Alter, Geschlecht, ethnischer Herkunft, körperlicher Beeinträchtigung, Religion, sexueller Orientierung oder anderer Diversitätskategorien erfolgen.

Insgesamt plädieren wir für die Integration von Diversitäts-, insbesondere Genderaspekten, in medizinische Curricula, in die Hochschuldidaktik, in Studiengangsstrukturen, in die Regularien zur Zulassung zum Studium sowie für deren Berücksichtigung bei der Planung, Durchführung und Bewertung von Prüfungen.

Beigetragen von (alphab.): Irene Brunk, Sabine Ludwig

Literaturverzeichnis

1. Comité Permanent Des Médecins Européens (CPME). CPME Policy on Sex and Gender in medicine. Brüssel: Comité Permanent Des Médecins Européens (CPME); 2016.
2. Ebenfeld M. Checkliste zur gender- und diversitätsbewussten Didaktik Berlin: Freie Universität Berlin; 2017. Zugänglich unter/available from: https://www.htw-berlin.de/fileadmin/HTW/Zentral/ZR_VI_-_Frauenfoerderung_und_Gleichstellung/GenderindieLehre/Checkliste_gender-diversitaet-Didaktik_Ebenfeld.pdf.
3. Ludwig S, Romero YR, Balz J, Petzold M. The use of quality assurance instruments and methods to integrate diversity aspects into health professions study programmes. MedEdPublish. 2018;7.
4. Clayton JA, Collins FS. Policy: NIH to balance sex in cell and animal studies. Nature News. 2014;509(7500):282-283.

Stellungnahme des Ausschusses Kulturelle Kompetenz und Global Health

Der Ausschuss begrüßt die Zielrichtung des Masterplan Medizinstudium 2020, die Lehre künftig stärker „an der Vermittlung arztbezogener Kompetenzen“ auszurichten und „die konsequente Orientierung am Patienten und seinen Bedürfnissen“ in den Mittelpunkt zu stellen. Auch die Betonung der Arzt-Patienten-Kommunikation sowie der sozialen und kommunikativen Kompetenzen der Studierenden unterstützen wir ausdrücklich. Um dieser Zielsetzung aber tatsächlich gerecht werden zu können, ist es unserer Ansicht nach notwendig, auch die soziokulturellen und internationalen Aspekte von Gesundheit, Medizin und ärztlichem Handeln systematisch zu berücksichtigen [1].

Im Hinblick auf die generelle Ausrichtung des Masterplan Medizinstudium 2020 stellen wir fest, dass neben dem demographischen Wandel und der Unterversorgung strukturschwacher Regionen – den zentralen Themen des Masterplans – auch Migration und eine wachsende sozio-kulturelle Diversität der Bevölkerung zu den zentralen Herausforderungen unserer Gesellschaft gehören und in Zukunft nicht an Bedeutung verlieren werden. Wir verweisen hier auf die jüngsten Empfehlungen der Hochschulrektorenkonferenz zur Internationalisierung der Curricula von 2017 [2].

Konkret ist es notwendig, im Hinblick auf die Kompetenzorientierung des Medizinstudiums, die Förderung kultureller Kompetenz ausdrücklich in das Regelstudium zu integrieren. Der NKLM bietet dazu bereits jetzt eine wichtige Orientierung. Im Hinblick auf die Arzt-Patienten-Kommunikation ist ferner eine systematische Integration von Lerninhalten zu Kommunikation über sprachliche Grenzen hinweg nötig, einschließlich der fachgerechten Nutzung technischer Hilfsmittel und der Zusammenarbeit mit Sprach- und Kulturmittlern. Auch zu diesem Thema bietet der NKLM geeignete Lernziele. Für Erhalt, Förderung und Ausbau sozialer und kommunikativer Kompetenzen von Studierenden gibt es schließlich einen Bedarf an bisher im Masterplan nicht angesprochenen Lehrangeboten, die die Reflexionsfähigkeit und das systematische Nachdenken über soziale, ethische und moralische Fragen fördern. Es ist vielfach dokumentiert, dass Lehrinhalte aus dem Themenfeld Kulturelle Kompetenz und Global Health (z.B. anknüpfend an studienbezogene Auslandsaufenthalte von Studierenden mit systematischer Vor- und Nachbereitung) hier ein erhebliches Potential besitzen [1], [2]. Insgesamt plädieren wir für eine systematische Integration internationaler und interkultureller Inhalte als Voraussetzung für eine zeitgemäße medizinische Ausbildung. Oder, wie es die Bundesvertretung der Medizinstudierenden in Deutschland (bvmd) bereits vor knapp 10 Jahren forderte: „Lehre am Puls der Zeit“ [3].

Beigetragen von (alphab.): Michael Knipper, Claudia Mews

Literaturverzeichnis

1. Mews C, Schuster S, Vajda C, Lindtner-Rudolph H, Schmidt LE, Bösner S, Güzelsoy L, Kressing F, Hallal H, Peters T, Gestmann M, Hempel L, Grützmann T, Sievers E, Knipper M. Cultural Competence and Global Health: Perspectives for Medical Education–Position paper of the GMA Committee on Cultural Competence and Global Health. GMS J Med Educ. 2018;35(3):Doc28. doi: 10.3205/zma001174

2. Hochschulrektorenkonferenz. Zur Internationalisierung der Curricula. Empfehlung der HRK-Mitgliederversammlung vom 9.5.2017 [press release]. Bonn: Hochschulrektorenkonferenz; 2017.
3. Bozorgmehr K, Last K, Müller A, Schubert K. Lehre am Puls der Zeit-Global Health in der Medizinischen Ausbildung: Positionen, Lernziele und methodische Empfehlungen. GMS Z Med Ausbild. 2009;26(2):Doc20. doi: 10.3205/zma000612

Stellungnahme des Ausschusses Personal- und Organisationsentwicklung in der Lehre

Der Ausschuss für Personal- und Organisationsentwicklung in der Lehre der GMA begrüßt viele der im Masterplan Medizinstudium 2020 beschriebenen Vorschläge zur Veränderung der Studienstruktur und der Ausbildungsinhalte im Medizinstudium ausdrücklich. Aus der Perspektive derjenigen, die durch die Qualifizierung der Lehrenden an den Medizinischen Fakultäten zur Qualitätsentwicklung und -sicherung der Lehre beitragen, stellen sich jedoch auch einige Fragen.

Eine der zentralen Maßnahmen des Masterplan Medizinstudium 2020 ist die Umsetzung einer kompetenzorientierten ärztlichen Ausbildung auf Grundlage des NKLM. Damit sind tiefgreifende strukturelle und curriculare Veränderungen an den Fakultäten verbunden. So machen etwa die stärkere curriculare Integration der ambulanten Medizin und der Ausbau der interprofessionellen Ausbildung neue Kooperationen und Konzepte – auch für die Personal- und Organisationsentwicklung in der Lehre erforderlich. Außerdem müssen Lehr- und Prüfungsformate neu entwickelt bzw. angepasst werden, um den verschiedenen im NKLM definierten Kompetenzbereichen gerecht zu werden.

Um diese Veränderungen bewältigen zu können, sind umfassende Angebote zur Personal- und Organisationsentwicklung, zusätzlich zu den bereits etablierten Maßnahmen an den Fakultäten notwendig. Diese Maßnahmen müssen auf die jeweiligen lokalen Besonderheiten der Fakultäten zugeschnitten werden, da die standortspezifischen Rahmenbedingungen großen Einfluss auf die Qualität von Lehre haben [1]. Im Rahmen des Medizindidaktik-Netzwerks (MDN), einer Arbeitsgruppe des Medizinischen Fakultätentages, an der alle Medizinischen Fakultäten in Deutschland beteiligt sind, wurden in den letzten Jahren Qualitätsstandards für Qualifizierungsmaßnahmen entwickelt und erfolgreich implementiert [2]. Die meisten Fakultäten verfügen bereits über eigene Angebote der medizindidaktischen Qualifizierung von Lehrenden, viele sind untereinander auf Landes- oder Bundeseben vernetzt und ermöglichen den Lehrenden die Teilnahme an Qualifizierungsmaßnahmen anderer Standorte. Diese bereits etablierten, akzeptierten und gut funktionierenden Strukturen sollten in erster Linie für den durch den Masterplan Medizinstudium 2020 notwendig werdenden Qualifizierungsbedarf genutzt werden.

Das gilt, neben den Forderungen nach der Integration für ambulante Medizin, interprofessionelle Lehre und mehr Wissenschaftlichkeit im Studium auch für die im Masterplan Medizinstudium 2020 unter Punkt 27 beschriebenen Schulungen für die OSCE-Prüfungen. Viele Fakultäten verfügen bereits über langjährige Erfahrungen mit diesem Prüfungsformat und haben entsprechende Qualifizierungsmaßnahmen für ihre Lehrenden entwickelt [3], [4]. Vor diesem Hintergrund erscheint es naheliegender, diese bereits bestehende Expertise als Grundlage für weitere Entwicklungen zu nutzen als in erster Linie auf zentrale Schulungen durch das IMPP zu setzen. Insgesamt erscheint es auch aus praktischen Erwägungen (zeitlicher Aufwand, Reisekosten, etc.) und vor dem Hintergrund der bereits etablierten Netzwerke und Strukturen sinnvoller, Multiplikatorinnen und Multiplikatoren zu schulen, die dann vor Ort entsprechende Qualifizierungsmaßnahmen anbieten und durchführen können, als die Lehrenden in der Breite zentral auszubilden. Die Standards und Qualitätskriterien für die entsprechenden Qualifizierungsangebote sowie die Konzepte für die Umsetzung könnten dabei von den Fakultäten selbst – innerhalb der vorhandenen Strukturen – entwickelt werden. Zudem ist sicherzustellen, dass

Lehrverantwortliche (z.B. in den Dekanaten, aber auch an einzelnen Institutionen und Kliniken) sowie Lehrende adäquat und rechtzeitig auf Neuerungen (z.B. Unterrichtsformate, Evaluationskonzepte und Prüfungen) vorbereitet werden. Dies muss professionell und unter Gewährleistung bundesweit gültiger Qualitätskriterien erfolgen.

Für viele der im Masterplan Medizinstudium 2020 beschriebenen Vorschläge sind somit neue und vielfältige Angebote der Personal- und Organisationsentwicklung erforderlich. Diese können aufgrund der dargestellten langjährigen Erfahrungen und angesichts der starken Vernetzung untereinander am besten von den Fakultäten selbst konzipiert und durchgeführt werden. Erforderlich sind dazu allerdings umfangreiche personelle und finanzielle Ressourcen, die den Fakultäten zusätzlich zur Verfügung gestellt werden müssen.

Literaturverzeichnis

1. Giesler M, Karsten G, Ochsendorf F, Breckwoldt J. Rahmenbedingungen für exzellente Lehre in der Medizin; Das Frankfurter Modell der Rahmenbedingungen zur Sicherung der Lehr-und Lernqualität. GMS J Med Educ. 2017;34(4):Doc46. doi: 10.3205/zma001123
2. Lammerding-Koeppel M, Ebert T, Goerlitz A, Karsten G, Nounla C, Schmidt S, Stosch C, Dieter P. German MedicalTeachingNetwork (MDN) implementing national standards for teacher training. Med Teach. 2016;38(4):378-384. doi: 10.3109/0142159X.2015.1047752
3. Müller S, Dahmen U, Settmacher U. Objective Structured Clinical Examination (OSCE) an Medizinischen Fakultäten in Deutschland–eine Bestandsaufnahme. Gesundheitswesen. 2018;80(12):1099-1103. doi: 10.1055/s-0042-116435
4. Graf J, Smolka R, Holderried F, Wosnik A, Lammerding-Köppel M, Mohr D, Vlad E, Nikendei C, Zipfel S, Herrmann-Werner A. Ten Years of Objective Structured Clinical Examination at the Medical Faculty of Tübingen, Germany: Item Analysis and Students' Satisfaction. J Health Sci Educ. 2018;2(2):1-7. doi: 10.0000/JHSE.1000133

Stellungnahme des Ausschusses Digitalisierung – Technologie-unterstütztes Lernen und Lehren

Der von der Bund-Länder AG am 31.3.2017 beschlossene Masterplan Medizinstudium 2020 enthält eine Vielzahl möglicher Maßnahmen und zukünftiger Schwerpunkte zur Reform des Medizinstudiums. Obwohl der aktuelle Koalitionsvertrag und das Hochschulforum Digitalisierung dem Stellenwert der Digitalisierung höchste Bedeutung beimessen, wird diese erstaunlicherweise mit keinem Wort im Masterplan Medizinstudium 2020 erwähnt. Dies ist allein dadurch schon erstaunlich, da die Digitalisierung einen weitreichenden Wandel in nahezu allen Bereichen der Gesellschaft hervorgerufen hat [1], [2]. Auch die Strukturen der Lehr- und Lernorganisation sowie die Rollen und Anforderungsprofile von Studierenden und Lehrenden haben sich verändert. Dabei entstehen neue didaktische Möglichkeiten hinsichtlich der Kompetenz- und Wissensvermittlung, aber auch Herausforderungen, auf die die medizinischen Hochschulen entsprechend reagieren müssen [3], [4]. Ein Paradigmenwechsel hat die Rolle der Lehrpersonen von Wissensvermittelnden hin zu „Wissensbereitstellenden" verändert. Die veränderten Rollen der Lehrpersonen, die nicht mehr alleinige Bereitstellen von Wissen und Informationen sind, sondern vielmehr Lehrinhalte kollektiv suchen, entwickeln und diskutieren, erfordert andere Kompetenzen [5] und können durch die Nutzung digitaler Lehrformate unterstützt werden [6].

Es wird durch den GMA-Ausschuss Digitalisierung prognostiziert, dass die Digitalisierung im Gesundheitswesen bereits für heutige Medizinstudierende - und noch viel mehr für zukünftige Studierendengenerationen - wichtige Themen- und Arbeitsbereiche bestimmen wird. Derzeit gibt es zwei große Herausforderungen, auf die das Medizinstudium adäquat reagieren muss, die Digitale Lehr- und Lerntechnologien sinnvoll und flächendeckend einsetzten sowie digitale Kompetenzen vermitteln [7].

Digitale Lehre

In der medizinischen Ausbildung halten zunehmend neue digitale Szenarien, Konzepte und Methoden Einzug in die Lehre [8]. Im Gesundheitswesen wird der Ausbildung und dem Erwerb sowohl professionsspezifischer als auch interprofessioneller Kompetenzen eine zunehmende Bedeutsamkeit zugesprochen, für die spezifische Lehrformate erprobt und etabliert werden müssen [9]. Die Wirksamkeit von Blended-Learning-Szenarien konnte bereits in einigen Studien nachgewiesen werden [10]. Es zeigt sich ebenfalls, dass sich neue und innovative technologie-unterstützte Lern- und Lehrmethoden wie die Inverted-Classroom-Methode langsam etablieren [11]. Nicht zuletzt bieten die heute an vielen Stellen bereits eingesetzten informationstechnologischen Werkzeuge und Methoden (Lehrvideos, Open Educational Resources, TED u.a. elektronisches (self)Assessment, Simulationen, Virtuelle Patienten) einen so hohen didaktischen Mehrwert, dass Universitäten schwerlich darauf verzichten können [12], [13]. Die große Anzahl erfolgreicher (v.a. kommerzieller) E-Learning-Angebote ist ein deutlicher Indikator für die Sinnhaftigkeit des Einsatzes digitaler Medien im Rahmen der medizinischen Aus- sowie Weiterbildung und relevant für eine praxisnahe Ausbildung.

Des Weiteren erfordert die moderne Lehre in der hochschulmedizinischen Ausbildung anpassungsfähige informationstechnologische Infrastrukturen, wie Lernplattformen, um innovative Lehrkonzepte nicht von vornherein zu erschweren. Förderungsmaßnahmen, wie durch die Digitalisierungsstrategie des Bundes in Aussicht gestellt, sind bei gegebenen knappen Mittelzuweisungen an die medizinischen Fakultäten absolut notwendig.

Digitale Kompetenzen

Ein weiteres Beispiel sind die Stichworte „BigData“ für die Forschung oder „Telemedizin“ für die zukünftige Arbeitswelt von Ärztinnen und Ärzten. Die Entwicklung neuer informationstechnologischer Werkzeuge, wie elektronischen Patientenakten, Krankenhaus- und Röntgeninformationssystemen oder elektronischen Gesundheitskarten sind heute im Berufsalltag der Ärztinnen und Ärzten sehr bedeutsam. Schon während des Medizinstudiums sollte deshalb der Umgang mit diesen Werkzeugen, aber auch die kritische Auseinandersetzung mit möglichen informationstechnologischen Weiterentwicklungen und deren Folgen für die Medizin und die Gesellschaft gefordert und gefördert werden. Die im Masterplan erwähnten arztbezogenen Kompetenzen müssen dringend auch die digitalen Kompetenzen mitberücksichtigen, welche als Anforderung der Kompetenzorientierung von Hochschulstudiengängen laut der Bologna-Reform beschrieben werden. Ausbildungsinhalte des Medizinstudiums sollten folgerichtig nicht nur wissenschaftliche Erkenntnisse und praktische Fertigkeiten, sondern auch die „digitale Kompetenz“ zur Nutzung aktueller und zukünftiger Informationstechnologien miteinschließen. Curriculare Konzepte zur Vermittlung digitaler Kompetenzen, wie das bereits existierende Curriculum „Medizin im digitalen Zeitalter“ der Universitätsmedizin Mainz [14] sollten breiter eingesetzt werden.

Telemedizin

Auf S. 4 des Masterplan Medizinstudium 2020 wird postuliert, „die medizinischen Versorgungsprozesse werden immer komplexer. In Zukunft wird daher eine arbeitsteilige Zusammenarbeit mit mitbehandelnden bzw. hinzuzuziehenden Ärztinnen und Ärzten anderer Fachrichtungen […] eine noch stärkere Rolle spielen als bisher.“ Die naheliegende Forderung einer Schulung und Stärkung der kommunikativen Kompetenzen soll an dieser Stelle um die Forderung der Aufnahme von Lernzielen zum Umgang mit relevanten informationstechnologischen Entwicklungen und Konzepten ergänzt werden. Solche Lernziele sind bereits im NKLM aufzufinden (Bsp.: 10.7.1, 11.2.3, und besonders 14c.6.3). Es ist davon auszugehen, dass es auch auf Seiten der Lehre noch keine fertigen Konzepte zur Vermittlung von beispielsweise der NKLM-Lernziele 10.7.1.2 „im klinischen Arbeitsplatzsystem (KAS) Untersuchungen anfordern, Befunde dokumentieren …“ oder 10.7.1.5 „Lösungen der Telemedizin patientenorientiert einsetzen und Rahmenbedingungen der Gesundheitstelematik erläutern“ gibt. Es erscheint durchaus angemessen, auch auf diese große didaktische Herausforderung schon im Masterplan Medizinstudium 2020 direkt einzugehen, zumal die für einen sinnvollen Praxisbezug schon im Studium erforderlichen Lerninhalte und Vermittlungsmethoden vermutlich noch an keiner Stelle suffizient existieren.

Abschließend ist es von hoher Bedeutung, dass aktuelle Barrieren der Digitalisierung, wie beispielsweise geringe Medienkompetenz der Lehrenden, das Fehlen einer einheitlichen Regelung zur Anrechnung von Lehrdeputat von digitaler Lehre [15] und die mangelnde Unterstützung der Institutionen abgebaut, bzw. reduziert werden.

Der Ausschuss Digitalisierung fordert daher die Entwicklung einer nationalen Digitalisierungsstrategie im Masterplan Medizinstudium 2020. Die Entwicklungen der Digitalisierung müssen in der zukünftigen medizinischen Ausbildung abgebildet werden. Berücksichtigt werden sollen dabei sowohl die Studierenden als auch die Mitglieder der Fakultäten und weiterer Entscheidungsträger. Nur mit einer einheitlichen Integration der Digitalisierung in die medizinische Ausbildung können Ärztinnen und Ärzte auf die heutigen und zukünftigen Herausforderungen der modernen Berufswelt vorbereitet werden.

Konkret fordern wir:

- Die Implementierung von Themen der Digitalisierung in die studentische Ausbildung
- Eine nationale Initiative "Medizinische Ausbildung im digitalen Zeitalter"
- Die Stärkung der hochschuldidaktischen Ausbildung, insbesondere der Fachgebiete Medienpädagogik und Mediendidaktik
- Die Implementierung der Inverted-Classroom-Methode
- Die Stärkung und Verbreitung freier Bildungsressourcen nach der Open Educational Resources-Idee
- Die Nutzung "virtueller" Patienten
- Den Einsatz web-/cloud-basierter Anwendungen (Kommunikations-Plattformen; Social Media; Artefakt-Gestaltung mittels Apps wie z.B. Lehr-Videos, -Comics, -Blogs, -Internetseiten, MindMaps, Concept Maps, Storytelling, Document / Media Sharing)
- Klärung und Vereinheitlichung der Anrechnung des Lehrdeputats für digitale Lehre.

Verfasst als kooperatives Projekt aller Ausschussmitglieder

Literaturverzeichnis

1. Bischof L, von Stuckrad T. Die digitale (R)evolution? Chancen und Risiken der Digitalisierung akademischer Lehre. Gütersloh: CHE gemeinnütziges Centrum für Hochschulentwicklung; 2013.
2. Hochschulforum Digitalisierung. The Digital Turn – Hochschulbildung im digitalen Zeitalter. Berlin: Hochschulforum Digitalisierung; 2016.
3. Handke J. Handbuch Hochschullehre Digital: Leitfaden für eine moderne und mediengerechte Lehre. Marburg: Tectum Wissenschaftsverlag; 2015.
4. Hochschulforum Digitalisierung. Diskussionspapier - 20 Thesen zur Digitalisierung der Hochschulbildung. Berlin: Hochschulforum Digitalisierung; 2015.
5. Lackner E, Kopp M. Lernen und Lehren im virtuellen Raum. Herausforderungen, Chancen, Möglichkeiten. In: Rummler K, ed. Lernräume gestalten - Bildungskontexte vielfältig denken. Medien in der Wissenschaft. Münster: Waxmann; 2014. p.174-186
6. Rummler K. Lernräume gestalten - Bildungskontexte vielfältig denken. Münster: Waxmann; 2014.
7. Haag M, Igel C, Fischer MR, German Medical Education Society (GMA), Committee "Digitization - Technology-Assited Learning and Teaching". Digital Teaching and Digital Medicine: A national initiative is needed. GMS J Med Educ. 2018;35(3):Doc43. doi: 10.3205/zma001189
8. Kuhn S, Frankenhauser S, Tolks D. Digitale Lehr-und Lernangebote in der medizinischen Ausbildung. Bundesgesundheitsblatt Gesundheitsforschung Gesundheitsschutz. 2018;61(2):201-209. doi: 10.1007/s00103-017-2673-z
9. Fabry G, Fischer MR. Medical Education in Germany–Work in Progress. GMS Z Med Ausbild. 2014;31(3):Doc36. doi: 10.3205/zma000928
10. Stegmann K, Fischer F. Auswirkungen digitaler Medien auf den Wissens-und Kompetenzerwerb an der Hochschule. München: Ludwig-Maximilian-Universität; 2016.
11. Tolks D, Bischoff T, Bauer D. Eine Einführung in die Inverted-Classroom-Methode in der medizinischen Ausbildung. In: Jahrestagung der Gesellschaft für Medizinische Ausbildung (GMA); 14.09.-17.09.2016; Bern. Düsseldorf: German Medical Science GMS Publishing House; 2016. DocWS-P04-323. doi: 10.3205/16gma007

12. Guo PJ, Kim J, Rubin R. How video production affects student engagement: an empirical study of MOOC videos. Proceedings of the first ACM conference on Learning@ scale conference; 2014.
13. Hege I, Kononowicz AA, Tolks D, Edelbring S, Kuehlmeyer K. A qualitative analysis of virtual patient descriptions in healthcare education based on a systematic literature review. BMC Med Educ. 2016;16(1):146. doi: 10.1186/s12909-016-0655-8
14. Kuhn S, Kadioglu D, Deutsch K, Michl S. Data Literacy in der Medizin. Onkologe. 2018;24(5):368-377.
15. Müller C, Füngerlings S, Tolks D; Arbeitsgruppe E-Learning des Komptenznetzes Medizinlehre Bayern. Teaching load–a barrier to digitalisation in higher education? A position paper on the framework surrounding higher education medical teaching in the digital age using Bavaria, Germany as an example. GMS J Med Educ. 2018;35(3):Doc34. doi: 10.3205/zma001180

Stellungnahme des Ausschusses Prüfungen

Im Masterplan Medizinstudium 2020 steckt Potential zur Weiterentwicklung der Prüfungskultur in der medizinischen Ausbildung in Deutschland, auch der Ärztlichen Prüfung. Dieses ergäbe sich aus einer konsequenten Umsetzung des übergeordneten Leitgedankens der kompetenzorientierten Ausbildung geradezu notwendig.

Zwar verzichtet der Masterplan Medizinstudium 2020 auf eine Definition von Kompetenz, referenziert aber mehrfach den NKLM [1], der sich wiederum auf Weinerts Definition [2] bezieht, den *„bei einem Individuum verfügbaren oder durch sie erlernbaren kognitiven Fähigkeiten und Fertigkeiten, um bestimmte Probleme zu lösen, sowie die damit verbundenen motivationalen, volitionalen und sozialen Bereitschaften und Fähigkeiten, um die Problemlösungen in variablen Situationen erfolgreich und verantwortungsvoll nutzen zu können.“* Was bedeutet dies nun für Prüfungen? Frank und Kollegen destillieren in einer systematischen Analyse von Definitionen kompetenzbasierter Ausbildung heraus, welche zentrale Schnittmenge sich für eine kompetenzorientierte Prüfungskultur ergibt: Demzufolge orientieren sich Prüfungen an vordefinierten Standards oder Meilensteinen, die den Fortschritt auf das definierte Ausbildungsziel abbilden. Prüfungsleistungen wären demzufolge kriteriumsorientiert zu bewerten, so dass der Lernerfolg der Lernenden nicht untereinander, sondern in Bezug auf ebendiese definierten Standards bewertet wird. Dies schließe auch nicht aus, dass Prüfungshürden eingebaut werden, die es zu überwinden gelte, bevor man im Curriculum weiter voranschreiten dürfe [3].

Der erste Punkt verweist direkt auf das didaktische Konzept des *Constructive Alignment* [4], also die durch Lernziele koordinierte Abstimmung von Unterricht, Lernen und Prüfungen zugunsten der Sinnbildung im Lernenden. Betrachtet man nun im Masterplan Medizinstudium 2020 die zumindest für einige Fakultäten neuen Ausbildungsinhalte Arzt-Patienten-Kommunikation und Zusammenarbeit im interdisziplinären bzw. interprofessionellen Team sowie das wissenschaftliche Arbeiten, stellt sich die Frage, wieso diese im Sinne ebendiesen Constructive Alignments nicht auch im weiteren Verlauf als Prüfungsinhalte genannt werden. Während einerseits den Fakultäten hier ausreichend Gestaltungsspielraum zu belassen ist, entwertet die Nichtnennung dieser Prüfungsinhalte im gleichen Schriftzug deren Relevanz im Vergleich zu anderen Studieninhalten.

Für die Umsetzung gibt es glücklicherweise bereits Erfahrungen, z.B. patientenorientierte Kommunikation in entsprechend verfassten OSCE-Stationen zu prüfen [5], [6], oder in OSCE-Stationen standardisierte Teammitglieder einsetzen [7], [8]. Leider versäumt es das Maßnahmenpapier aber, die „Befähigung zum wissenschaftlichen Arbeiten“ genauer zu operationalisieren. Hier ist zu hoffen, dass die Fakultäten bei der Interpretation dieser Maßnahme nicht nur die forschungsorientierten Kompetenzen des *Gelehrten* (Scholar) berücksichtigen.

Geht es um die Prüfungsformate, die den Studieninhalten bzw. der Umsetzung der Maßnahmen dienlich sind, sind Gelassenheit und Zuversicht angebracht. Gelassenheit, weil die in der Literatur beschriebenen und in Deutschland teils pilotierten teils etablierten Formate zuerst einmal ausreichend scheinen, die Herausforderungen des Maßnahmenpapiers umzusetzen. Die Quintessenz der internationalen Literatur zu kompetenzorientierter Ausbildung in der Medizin kommt zum Schluss, dass es in diesem Zuge individuelle Bedürfnisse der Studierenden zu bedienen gilt, die Studierenden ihr Lernen aktiv managen und regelmäßiges Self-Assessment betreiben und deren Lernfortschritt

regelmäßig erhoben und zurückgemeldet werden muss [3]. Dies ist nichts als ein Plädoyer für mehr Progress Testing in Kombination mit Mentorenprogrammen, einer lebendigen Reflexions- und Feedbackkultur und Lern-Portfolios, alles Angelegenheiten, mit der die deutsche Hochschulmedizin, der Politik vielleicht vorgreifend, bereits Erfahrungen gesammelt hat [9], [10]. Gelassenheit auch deswegen, weil ein geeignetes Format der summativen kompetenzorientierten Prüfung – der bereits erwähnte OSCE – in Deutschland bereits weit verbreitet ist und an zahlreichen Fakultäten damit intensive Erfahrungen gemacht wurden: Ein Format, das dezidiert zur Prüfung klinischer Kompetenz gedacht ist [11] und in Variationen z.B. in der Schweiz [12], den USA [13] und in Kanada [14] einen Teil der Abschlussprüfung des Medizinstudiums darstellt. Es wäre wünschenswert, dass bei der Entwicklung der neuen Ärztlichen Prüfung alle Stakeholder, auch die Fakultäten aktiv in den Entwicklungsprozess einbezogen werden, was sich in der Schweiz als vorteilhaft erwies (12).

Selbstverständlich muss berücksichtigt werden, dass der logistische und finanzielle Aufwand, einen OSCE durchzuführen, erheblich ist [15], weswegen nicht vergessen werden sollte, dass etablierte schriftliche Prüfungsformate weiterhin zur Verfügung stehen. So z.B. sind Multiple Choice-Fragen und Key Feature-Fälle, soweit sie auf der Ebene Problemlösen prüfen (es sei an die Definition der Kompetenz erinnert), weiterhin relevante Formate - auch wenn der Aufwand, gute Fragen bzw. Fälle zu erstellen, auch hier hoch ist und die Erfahrung der meisten AutorInnen darin oft nur gering. Die elektronischen Unterstützung würde den Einsatz mancher dieser Formate zwar erleichtern, wirft aber selbst neue Fragen bzgl. Logistik und Finanzierung auf.

Und schließlich ist Zuversicht angebracht, da das Maßnahmenpapier erklärt, die Anzahl und Notenpflicht der Leistungsnachweise würde überprüft. Die schlichte Anzahl summativ durchzuführender Prüfungen, die nur bedingt lernförderlich scheinen, sondern wohl primär juristisch belastbaren Bestehens-/Nichtbestehens-Entscheiden dienen sollen, steht in eindeutigem Gegensatz zu einer Kultur des Assessment for Learning [16], also des Prüfens nicht mit dem Ziel, das Gelernte zu messen, als vielmehr mit dem Ziel, das Lernen zu unterstützen. Es bleibt zu hoffen, dass neben der Diskussion über die Benotung auch die Bewertung der Prüfungsleistung im Staatsexamen weiter erörtert wird. Zwar gibt es Noten in der Ärztlichen Prüfung bzw. ihren Vorläufern seit wenigstens 1869 [17], jedoch ist der vorrangige Zweck einer Abschlussprüfung, die Entscheidung zu ermöglichen, ob einE PrüfungskandidatIn die Mindestqualifikation besitzt, um unter Aufsicht der Weiterbildungsbefugten an RealpatientInnen weiter zu lernen. Es liegt auf der Hand, dass dies eine Ja/Nein-Frage ist, weswegen diese Prüfung dafür optimiert sein sollte, ebendiese Frage zu beantworten [18]. Der Aufgabenmix sanktionierender Prüfungen (Staatsprüfung, summative fakultäre Prüfungen) muss hierfür optimiert sein. Für diese gilt es auch, die Ablösung der bestenfalls historisch legitimierten 60%-Bestehensgrenze zu diskutieren und eine dem Geiste der Kompetenzorientierung angemessene Kriteriumsorientierung anzudenken. Im Sinne von Kanes Rahmenwerk zur Validierung von Tests umreisst der Masterplan Teile der sog. Interpretations-Debatte [19], [20] und gemeinsam mit dem IMPP muss auch die Validierungs-Debatte noch geführt werden.

Zwei Kuriositäten fallen noch auf: Einerseits, dass trotz der im Masterplan Medizinstudium 2020 geforderten Kompetenzorientierung von Lehre und Prüfungen immer noch eine Fächerlogik der Curricula postuliert wird, z.B. bei den in den mündlichen Ärztlichen Prüfungen zu berücksichtigenden Inhalten. Nun sind aber modernere Curricula (wie auch Kliniken) in Deutschland teilweise schon jenseits der Fächer angelangt. Der Beratungsanlass eines Patienten, weswegen er ärztliche Hilfe in Anspruch nimmt, ist schließlich ebenso wenig

fachorientiert wie die Kompetenzen, die das Lösen des Patientenproblems ermöglichen. Eine Loslösung von der Fächerlogik würde auch die Umsetzung des OSCE im Staatsexamen logistisch und psychometrisch wesentlich erleichtern, sodass nicht pro Wahlfach ein eigener Subtyp der Prüfung vorgehalten werden muss. Eine wahrlich kompetenzorientierte Prüfungskultur und die Überarbeitung des NKLM sollten Restriktionen einer erzwungenen Fächerzuordnung tunlichst vermeiden. Kurios als zweites, dass mit dem Masterplan Medizinstudium 2020 erstmals das Format eines universitären Leistungsnachweises vorgegeben wird und damit den Fakultäten ein Freiheitsgrad abgerungen wird.

Beigetragen von (alphab.): Daniel Bauer, Monika Himmelbauer, Maren März

Literaturverzeichnis

1. Fischer MR, Bauer D, Mohn K, NKLM-Projektgruppe. Finally finished! National Competence Based Catalogues of Learning Objectives for Undergraduate Medical Education (NKLM) and Dental Education (NKLZ) ready for trial. GMS Z Med Ausbild. 2015;32(3):Doc35. doi: 10.3205/zma000977
2. Weinert FE. Vergleichende Leistungsmessung in Schulen - eine umstrittene Selbstverständlichkeit. In: Weinert FE, ed. Leistungsmessungen in Schulen. Weinhein, Basel: Beltz Verlag; 2001. p.17-32
3. Frank JR, Mungroo R, Ahmad Y, Wang M, De Rossi S, Horsley T. Toward a definition of competency-based education in medicine: a systematic review of published definitions. Med Teach. 2010;32(8):631-637. doi: 10.3109/0142159X.2010.500898
4. Biggs J. Enhancing teaching through constructive alignment. High Educ. 1996;32(3):347-364.
5. Bachmann C, Hölzer H, Dieterich A, Fabry G, Langewitz W, Lauber H, Ortwein H, Pruskil S, Schubert S, Sennekamp M, Simmenroth-Nayda A, Silbernagel W, Scheffer S, Kiessling C. Longitudinales, bologna-kompatibles Modell-Curriculum" Kommunikative und soziale Kompetenzen": Ergebnisse eines interdisziplinären Workshops deutschsprachiger medizinischer Fakultäten. GMS Z Med Ausbild. 2009;26(4):Doc38. doi: 10.3205/zma000631
6. Cömert M, Zill JM, Christalle E, Dirmaier J, Härter M, Scholl I. Assessing communication skills of medical students in objective structured clinical examinations (OSCE)-A systematic review of rating scales. PloS one. 2016;11(3):e0152717. doi: 10.1371/journal.pone.0152717
7. Guise J-M, Deering SH, Kanki BG, Osterweil P, Li H, Mori M, Lowe NK. Validation of a tool to measure and promote clinical teamwork. Simul Healthc. 2008;3(4):217-223. doi: 10.1097/SIH.0b013e31816fdd0a
8. Wright MC, Phillips-Bute BG, Petrusa ER, Griffin KL, Hobbs GW, Taekman JM. Assessing teamwork in medical education and practice: relating behavioural teamwork ratings and clinical performance. Med Teach. 2009;31(1):30-38. doi: 10.1080/01421590802070853
9. Meinel FG, Dimitriadis K, von der Borch P, Störmann S, Niedermaier S, Fischer MR. More mentoring needed? A cross-sectional study of mentoring programs for medical students in Germany. BMC Med Educ. 2011;11(1):68. doi: 10.1186/1472-6920-11-68
10. Nouns ZM, Georg W. Progress testing in German speaking countries. Med Teach. 2010;32(6):467-470. doi: 10.3109/0142159X.2010.485656
11. Harden R, Stevenson M, Downie WW, Wilson G. Assessment of clinical competence using objective structured examination. Br Med J. 1975;1(5955):447-451.

12. Guttormsen S, Beyeler C, Bonvin R, Feller S, Schirlo C, Schnabel K, Schurter T, Berendonk C. The new licencing examination for human medicine: from concept to implementation. Swiss Med Wkly. 2013;143:w13897. doi: 10.4414/smw.2013.13897. eCollection 2013
13. Papadakis MA. The Step 2 clinical-skills examination. N Engl J Med. 2004;350(17):1703-1705.
14. Reznick R, Smee S, Rothman A, Chalmers A, Swanson D, Dufresne L, Lacombe G, Baumber J, Poldre P, Levasseur L, et al. An objective structured clinical examination for the licentiate: report of the pilot project of the Medical Council of Canada. Acad Med. 1992;67(8):487-494.
15. Brown C, Cleland J, Walsh K. The costs of medical education assessment. Med Teach. 2016;38(2):111-112. doi: 10.3109/0142159X.2015.1105946
16. Schuwirth LW, Van der Vleuten CP. Programmatic assessment: from assessment of learning to assessment for learning. Med Teach. 2011;33(6):478-485. doi: 10.3109/0142159X.2011.565828
17. Rentschler HE. Zur Geschichte der ärztlichen Prüfungen. Med Ausbild. 1988;5(2):77-82. Zugänglich unter/available from: https://gesellschaft-medizinische-ausbildung.org/publizieren/zma-archiv/1988.html
18. AERA, APA, NCME. The Standards for Educational and Psychological Testing. Washington, DC: American Psychological Association; 2013. Zugänglich unter/available from: https://www.apa.org/science/programs/testing/standards
19. Kane M. Validating high-stakes testing programs. Educ Measurement. 2002;21(1):31-41. doi: 10.1111/j.1745-3992.2002.tb00083.x
20. Fischer V. Gütekriterien bei universitären Prüfungen im Lichte von Kanes Rahmenwerk. Wien Med Wochenschr. 2019;169(5-6):110-118. doi: 10.1007/s10354-018-0661-z

Stellungnahme des Ausschusses Lehrevaluation

Im Masterplan Medizinstudium 2020 werden Maßnahmen aufgeführt, die mit curricularen Änderungen einhergehen werden. Eine gut begründete und sorgfältig konzipierte Evaluation ist dafür unabdingbar. Evaluation ist hier im Sinne von Cronbach zu verstehen, indem Informationen gesammelt und verarbeitet werden mit dem Ziel, Entscheidungen über ein Curriculum zu fällen [1]. Diese Entscheidungen können Curriculumverbesserungen betreffen, auf Individuen bezogen sein (z.B. Ermittlung der Bedürfnisse, Lernfortschritt) und/oder sich auf administrative Regelungen beziehen. Nach Gibson werden des Weiteren vier grundlegende Dimensionen der Lehrqualität definiert (Strukturen, Prozesse, Lehrende und „Outcomes") [2]. Diese werden von den im Masterplan Medizinstudium 2020 genannten Maßnahmen beeinflusst und sollten entsprechend auch Gegenstand der begleitenden Evaluation sein.

Da die Evaluation künftig nicht nur der fakultätsinternen Bewertung der Lehre dienen wird, sondern insbesondere auch zur Erfolgskontrolle und Bewertung der politischen Maßnahmen notwendig ist, sollten frühzeitig fakultätsübergreifende Evaluationsziele und -methoden konsentiert werden. Dies ist wichtig, damit Daten erhoben werden können, die den Nachweis bringen, ob und inwiefern die geforderten Maßnahmen tatsächlich wirksam sind. Die Evaluationskonzepte müssen sich an internationalen Standards orientieren, und es ist empfehlenswert, die (Neu-)Entwicklung geeigneter Evaluationsinstrumente auch wissenschaftlich zu begleiten. Hierfür müssen adäquate finanzielle Ressourcen zur Verfügung gestellt werden. Eine zentrale Koordination durch den MFT unter Einbindung der GMA (z.B. Ausschuss Lehrevaluation und Ausschuss für Methodik der Ausbildungsforschung) ist empfehlenswert. Zusätzlich sollten alle Maßnahmen bereits vor einer Neufassung der ÄApprO unter dem Aspekt der Anwendbarkeit, der Wirksamkeit bzw. Übertragbarkeit kritisch beleuchtet werden. Ein wichtiger Aspekt, der vor Verabschiedung der neuen ÄApprO im Fokus stehen sollte, sind die Kosten-Nutzen-Relationen der angestrebten Maßnahmen.

Im Folgenden wird auf einzelne konkrete Maßnahmen eingegangen, für die eine begleitende Evaluation wesentlich sein wird.

<1> Die ärztliche Ausbildung wird kompetenzorientiert ausgestaltet

Die Ausgestaltung der ärztlichen Ausbildung anhand von Kompetenzen muss sich einerseits in der Etablierung kongruenter Prüfungen niederschlagen (Ergebnis-Ebene). Zugleich aber muss im Rahmen der begleitenden Evaluation zuverlässig untersucht werden, ob der Schritt von einer primär wissensbasierten zu einer kompetenzbasierten Ausbildung auch auf struktureller und prozeduraler Ebene vollzogen wurde. Als mögliches Instrument zur Erhebung der hierfür benötigten Daten eignen sich fakultätsübergreifende Befragungen. Auch studentische Evaluationen können zu diesem Zweck eingesetzt werden.

<5> Die Studien- und Prüfungsinhalte werden künftig stärker auf die wesentlichen Lernziele fokussiert.

In dieser Maßnahme wird die Passung zwischen Lernzielen und Lehraktivitäten („constructive alignment") angesprochen. Es existieren Evaluationsinstrumente, mit denen das Ausmaß dieser Passung anhand studentischer Einschätzungen erfasst werden kann; diese Instrumente werden jedoch bisher im Medizinstudium kaum eingesetzt. Neben der studentischen Evaluation kann auch die o.g. Befragung der medizinischen Fakultäten

Aufschluss darüber geben, welche Maßnahmen zur Erreichung der hier genannten Ziele ergriffen wurden.

<10> Zur strukturierten Vermittlung wissenschaftlicher Kompetenzen wird künftig ein Leistungsnachweis vorgegeben. Grundlage dafür sind die Empfehlungen des Wissenschaftsrates.

Die Vermittlung wissenschaftlicher Kompetenzen wird im Medizinstudium künftig deutlich an Gewicht gewinnen. Aktuell fehlen Prüfungs- und Evaluationsmethoden, mit denen die Qualität der diesbezüglichen Ausbildung reliabel und valide überprüft werden könnten. Daher ist es erforderlich, parallel zur Entwicklung der Lehrformate (die sich nicht in Laborrotationen erschöpfen sollten) auch zum Lernziel kongruente Prüfungs- und Evaluationsinstrumente zu entwickeln. Die Evaluation der Qualität der Lehre sollte in erster Linie durch Studierende erfolgen; dabei sollte ein besonderer Schwerpunkt auf das Lehr-/Lernergebnis liegen. Im Gegensatz hierzu sollte die strukturelle Verankerung der neuen Lehre im Curriculum im Rahmen fakultätsübergreifender Befragungen evaluiert werden.

<15> Lehrpraxen werden verstärkt in die ärztliche Ausbildung einbezogen.

Nicht alle in Lehrpraxen tätigen Ärztinnen und Ärzte sind medizindidaktisch qualifiziert. Eine Evaluation der hier erbrachten Lehrleistung ist besonders wichtig, um einerseits die Qualität der Lehre überprüfen zu können und andererseits gezielte Fortbildungsangebote für Lehrende ohne weitergehende didaktische Qualifikation erarbeiten zu können. Problematisch ist, dass je Praxis nur eine geringe Anzahl von Studierenden unterrichtet wird. Dies stellt eine quantitative Evaluation hinsichtlich der Reliabilität und Validität vor schwierige Aufgaben. Es erscheint notwendig, speziell für dieses Setting qualitative Evaluationsmethoden zu entwickeln, die auch eine enge Ankopplung an die jeweils zuständige Fakultät einschließen.

<27> Das IMPP entwickelt für die Ein- und Durchführung der OSCE-Prüfungen in der Ärztlichen Prüfung verbindlichen Vorgaben; dies beinhaltet auch die Standardisierung der mündlich-praktischen Prüfung am Patientenbett und Vorgaben zur Prüferqualifizierung.
<28> Wir erwarten, dass die Universitäten diese Vorgaben in der Qualifizierung und Fortbildung der Lehrenden sowie der Prüferinnen und Prüfer nachvollziehen.

Die Qualitätssicherung der hier benannten Schritte ist auch eine Form der Evaluation. Sofern qualitätssichernde Maßnahmen nicht als inhärenter Bestandteil der Prüfungsentwicklung vorgesehen sind, müssen sie mittels geeigneter (und auf die Prüfungsinhalte und -formate abgestimmter) Weise konzipiert und implementiert werden. Insbesondere die Standardisierung der Prüfungen sowie die Qualität der Prüferqualifizierung im Rahmen von Faculty Development-Maßnahmen sind einer quantitativen und qualitativen Evaluation zugänglich. Es wird empfohlen, diese qualitätssichernden Maßnahmen standortübergreifend zu konzipieren.

<33> Wir erwarten, dass die Hochschulen stärker als bisher von der Möglichkeit der Approbationsordnung für Ärzte Gebrauch machen und ihre Gestaltungsspielräume nutzen, um mehr Lehrkrankenhäuser auch im ländlichen Raum dauerhaft einzubinden.

Analog zu den zum Punkt <15> angestellten Überlegungen sind auch an Krankenhäusern, an denen die Voraussetzungen für die Anerkennung als akademisches Lehrkrankenhaus hinsichtlich der didaktischen Qualifikation der Ärztinnen und Ärzte nicht vollständig erfüllt sind, entsprechende fakultätsentwickelnde Maßnahmen anzustoßen und ihre Effektivität zu evaluieren. Dies schließt nicht nur eine Evaluation der Fakultätsentwicklungsprogramme

(hier: medizindidaktische Trainings) ein, sondern auch eine Beurteilung der Qualität der Lehre, die von den hier qualifizierten Ärztinnen und Ärzten geleistet wird. Diese Evaluation wird sich in erster Linie auf studentisches Feedback stützen; eine zentrale Organisation der Datenerhebung und -analyse erscheint ratsam.

Vor allem bezüglich der Punkte <10> und <15> ist die anstehende Entwicklungsarbeit erheblich und ohne eine entsprechende Finanzierung nicht umsetzbar. Für die fakultätsübergreifenden Evaluationen müssen Strukturen etabliert werden, die sicherstellen, dass einerseits Personal vorhanden ist, das über die erforderliche Kompetenz zur Datenerhebung und -analyse verfügt und die andererseits den Ansprüchen der Datenschutzgrundverordnung genügen können.

Beigetragen von (alphab.): Marianne Giesler, Tobias Raupach, Alexandra Scherg, Katrin Schüttpelz-Brauns

Literaturverzeichnis

1. Cronbach LJ. Evaluation zur Verbesserung von Curricula. In: Wulf C, ed. Evaluation Beschreibung und Bewertung von Unterricht, Curricula und Schulversuchen. München: R. Piper & Co. Verlag; 1972. p.41-59
2. Gibson KA, Boyle P, Black DA, Cunningham M, Grimm MC, McNeil HP. Enhancing evaluation in an undergraduate medical education program. Acad Med. 2008;83(8):787-793. doi: 10.1097/ACM.0b013e31817eb8ab

Stellungnahme des Ausschusses Methodik der Ausbildungsforschung

Im Masterplan Medizinstudium 2020 werden Maßnahmen, die sich aus best-practice-Beispielen und aus der Forschung ergeben, gleichwertig neben politisch motivierten Maßnahmen aufgeführt. So erscheinen Maßnahmen, die sich z.B. auf die Nachwuchsgewinnung in der Allgemeinmedizin beziehen, eher politisch motiviert als evidenzbasiert (Maßnahmen Nrs. 16, 17.1, 20; [1]). Die Medizinstudierenden in Deutschland sind sich der Bedeutung des Faches Allgemeinmedizin bewusst und sie sind durchaus willens, viel Zeit ihres Studiums darauf zu verwenden [2]. Das lässt daher eher die Erhöhung des Anteils an Wahlveranstaltungen der Allgemeinmedizin im Studium sinnvoll erscheinen [3]. Eine Verpflichtung aller Studierenden könnte sich ggf. sogar nachteilig auswirken, wenn z.B. der Eindruck entstünde, mit der Betonung der Allgemeinmedizin sei zunächst einmal eine Einschränkung der per se geringen (Wahl-) Freiheiten im Studium verbunden.

Andererseits muss das bloße Umsetzen als erfolgreich beschriebener Maßnahmen nicht zum Erfolg führen. Bereits in der ersten Leitlinie zu „Best Evidence Medical Education“ wurde betont, dass die Übertragbarkeit von Forschungsergebnissen und Konzepten stets zu überprüfen ist [4]. Dabei ist zum einen zu berücksichtigen, ob die Rahmenbedingungen („Setting“) vergleichbar sind. Zum anderen muss sichergestellt sein, dass es nicht zu (ungewollten) Modifikationen des zu übertragenden Ansatzes kommt, die die Wirksamkeit der Maßnahme gefährden. Daher können Ergebnisse, die im Rahmen der Ausbildungsforschung gewonnen werden, nicht generell übernommen werden, zumindest nicht ohne den Erfolg des Transfers durch Evaluation und ggf. wissenschaftliche Begleitung sicherzustellen.

In Maßnahme Nr. 29 („Ergänzend sind die Hochschulen aufgefordert, neue kompetenzorientierte Lehr- und Prüfungsformate zu erproben und weiterzuentwickeln“) wird dies als „Anwendungsforschung“ aufgegriffen [1]. Jedoch ist es unklar, inwiefern die angesprochene Zielgruppe in der Lage ist, diese Anforderungen kompetent zu erfüllen. Gibt es genügend personelle und materielle Ressourcen bzw. methodische Expertise, um die Effektivität neuer Lehr- und Prüfungsformate adäquat zu überprüfen? Falls dies nicht der Fall ist, bleibt diese Forderung nach der „Anwendungsforschung“ an der Oberfläche und ein reines Lippenbekenntnis, ohne den Anspruch zu erfüllen, wissenschaftliche Erkenntnis zu generieren. So konnten Reed et al. anhand von 210 Studien in der Medizinischen Ausbildungsforschung einen Zusammenhang zwischen der finanziellen Förderung der Studie und deren Qualität (Expertenrating, Zitationsrate, sowie Höhe des Impact Factors) aufzeigen [5].

Die vom Wissenschaftsrat bereits 2014 geforderte Stärkung und Ausweitung der Ausbildungsforschung [6] sollte daher durch die Einrichtung von entsprechenden Lehrstühlen, Instituten und Kompetenzzentren an den Medizinischen Fakultäten realisiert werden. Es ist wünschenswert, dass das BMBF Drittmittel für die wissenschaftliche Begleitung und Evaluation bereitstellt, da aktuell keine ausreichenden Ressourcen für die wissenschaftliche Überprüfung des etwaigen Effekts einer implementierten Maßnahme zur Verfügung stehen.

Gleichzeitig sollte darauf geachtet werden, den Masterplan nicht als alleinige Agenda für medizinische Ausbildungsforschung in Deutschland zu verwenden, um den Anschluss an internationale Forschung im Bereich von Medizindidaktik und Lehre nicht zu verpassen. An dieser Stelle bietet sich andererseits aber auch die Chance, Forschung zu betreiben, die

international von Interesse sein könnte und dadurch Deutschland als Standort von Ausbildungsforschung international sichtbar zu machen. Eine nachhaltige Institutionalisierung von Ausbildungsforschung sollte daher ein langfristiges Ziel sein.

Aus dem Vorherigen ergeben sich aus unserer Perspektive kurz gefasst folgende Empfehlungen:

- Neuerungen bzw. damit einhergehende Maßnahmen (z.B. Einführung von zusätzlichen bzw. alternativen Unterrichtsformaten) müssen durch nach Möglichkeit evidenzbasierte Minimalanforderungen definiert sein, aber ausreichend Spielraum bieten, um an z.B. lokale Gegebenheiten in Abstimmung mit dem MFT unter Einbindung der GMA angepasst zu werden.
- Eine Institutionalisierung der wissenschaftlichen Begleitung der Maßnahmen des Masterplans Medizinstudium 2020 inkl. der nachhaltigen Bereitstellung personeller und finanzieller Ressourcen ist unabdingbar, um den Prozess langfristig zu begleiten und wenn möglich noch vor Einführung der Novelle der ÄApprO zu modifizieren.

Beigetragen von (alphab.): Marianne Giesler, Wolf Hautz, Monika Himmelbauer, Jörg Marienhagen, Jan Matthes, Katrin Schüttpelz-Brauns

Literaturverzeichnis

1. Bundesministerium für Bildung und Forschung. Masterplan Medizinstudium 2020. Berlin: Bundesministerium für Bildung und Forschung; 2017. Zugänglich unter/available from: https://www.bmbf.de/files/2017-03-31_Masterplan%20Beschlusstext.pdf
2. Dafsari HS, Herzig S, Matthes J. A multi-centre student survey on weighing disciplines in medical curricula–a pilot study. GMS J Med Educ. 2017;34(2):Doc24. doi: 10.3205/zma001101
3. Jacob R, Kopp J, Schultz S. Berufsmonitoring Medizinstudenten 2014–Ergebnisse einer bundesweiten Befragung. Berlin: Kassenärztliche Bundesvereinigung (KBV); 2015.
4. Harden R, Grant J, Buckley G, Hart I. BEME Guide No. 1: Best evidence medical education. Med Teach. 1999;21(6):553-562.
5. Reed DA, Cook DA, Beckman TJ, Levine RB, Kern DE, Wright SM. Association between funding and quality of published medical education research. JAMA. 2007;298(9):1002-1009.
6. Wissenschaftsrat. Empfehlungen zur Weiterentwicklung des Medizinstudiums in Deutschland auf Grundlage einer Bestandsaufnahme der humanmedizinischen Modellstudiengänge. Dresden: Wissenschaftsrat; 2014.
