# Supplementary material for: Position of the advisory and executive board of the German Association for Medical Education (GMA) regarding the “masterplan for medical studies 2020”
Source: GMS J Med Educ. 2019 Aug 15;36(4):Doc46. doi: 10.3205/zma001254 (PMC6737258; doi:10.3205/zma001254)
Supplement: Student Selection Committee [german] [file JME-36-4-46-s-002.pdf]

Stellungnahme des Ausschusses Studierendenauswahl

Zum Thema Studierendenauswahl adressiert der Masterplan unterschiedliche Ziele, nämlich die Auswahl von Studierenden, die

i) mit hoher Wahrscheinlichkeit ihr Studium erfolgreich absolvieren.
ii) die beste Aussicht dafür bieten, gute Ärztinnen und Ärzte insbesondere in der Versorgung der Patientinnen und Patienten zu werden oder in der Wissenschaft und Forschung erfolgreich tätig zu sein.
iii) sich später als Ärzte in unterversorgten Regionen niederlassen.

Der Masterplan benennt mehrere Maßnahmen zum Erreichen dieser Ziele, u.a.:

a) Die Hochschulen sollen neben der Abiturnote mindestens zwei weitere Auswahlkriterien anwenden, die insbesondere die sozialen und kommunikativen Fähigkeiten sowie die Leistungsbereitschaft einbeziehen.
b) Unterstützung der Hochschulen bei der Anpassung ihrer Auswahlverfahren.
c) Weiterentwicklung von Zulassungsverfahren in der Weise, dass die ärztliche Versorgung in unterversorgten Regionen verbessert wird. Hierzu sollen bis zu 10% der Medizinstudienplätze an Bewerberinnen und Bewerber vergeben werden, die sich verpflichten, nach Abschluss des Studiums und der fachärztlichen Weiterbildung für bis zu zehn Jahre in der hausärztlichen Versorgung tätig zu sein.

Mit unserem Beitrag möchten wir aufzeigen, für welche Auswahlverfahren bereits Evidenzen vorliegen, dass sie zum angestrebten Erfolg führen.

Ad i) Studienerfolg

Da sich Studienerfolg relativ gut messen lässt, ist hier die Datenlage auch am übersichtlichsten: So haben sich weltweit für die Vergabe von Studienplätzen in der Humanmedizin kognitive Verfahren durchgesetzt, deren Ergebnisse mit hoher Wahrscheinlichkeit den erfolgreichen Abschluss des Studiums vorhersagen. Hier belegen nationale und internationale Studien, dass die Note der Hochschulzugangsberechtigung die größte Vorhersagekraft besitzt [1], [2]. Allerdings gibt es in zunehmendem Umfang Probleme durch eine stark steigende Zahl von Abiturienten mit Bestnoten und durch eine mangelnde Vergleichbarkeit der Abiturnoten z.B. in unterschiedlichen Bundesländern, weshalb das Bundesverfassungsgericht einen Ausgleichsmechanismus fordert [3]. Inkrementellen Erkenntnisgewinn bieten kognitive Tests, die auf einem Kontinuum eher wie der HAM-Nat Kenntnisse oder wie der TMS eine fachspezifische Studierfähigkeit prüfen [4], [5]. Nationale Studien zeigen eine prädiktive Validität von TMS und HAM-Nat für den kognitiven Studienerfolg in den ersten Semestern [6]. Die internationale Studienlage wertet Kenntnistests dabei höher als Fähigkeitstests. Prädiktive Validität und Fairness müssten jedoch für jeden einzelnen Test erneut nachgewiesen werden [1], [7]. Es gibt keine Studien, die eine Korrelation von vorheriger medizinnaher Berufstätigkeit, sozialem Engagement, Preisen im Bildungsbereich oder sportlichen Leistungen mit dem Studienerfolg zeigen.

Ad ii) Kompetenzen für die Patientenversorgung

Kognitive Fähigkeiten, die für die Patientenversorgung oder wissenschaftliches Arbeiten benötigt werden, werden auch in den unter i) genannten Verfahren adressiert. Bei den für den Arztberuf nötigen sozialen und kommunikativen Kompetenzen stehen vor allem die Kommunikations- und Teamfähigkeit im Fokus, die derzeit mittels klassischer Interviews,

multipler Mini-Interviews (MMI) im Sinne eines Interview-Parcours oder Situational Judgement Tests (SJT) erfasst werden sollen. Alle Messverfahren bergen die Gefahr, dass durch sozial erwünschte Antworten die Ergebnisse verfälscht werden. Zudem sind Aussagen zur prädiktiven Validität schwierig, da in Studium und Beruf kaum quantitative Daten zur psychosozialen Kompetenz erhoben werden. Weitere Probleme sind mangelnde Reliabilität klassischer Interviews und unklare Konstruktvalidität von Multiplen Mini-Interviews (MMI) und SJT [6]. Dennoch befinden sich SJT im Bereich der Studierendenauswahl bereits in Großbritannien, Belgien und Kanada im Einsatz und lassen wie die MMI moderate Zusammenhänge zwischen der jeweils adressierten Kompetenz und verwandten Studieninhalten z.B. in OSCE-Prüfungen erkennen [8-10]. Diese Ergebnisse ermutigen, weitere Anstrengungen in die Entwicklung theoretischer Erklärungsansätze zu investieren. Tatsächlich sind die Entwicklung von MMIs und SJTs Schwerpunkte in einem Forschungsprojekt, mit dem das BMBF die Fakultäten bei der Erforschung und Anpassung ihrer Auswahlverfahren im Rahmen des Masterplan Medizinstudium 2020 unterstützt. Auch hier gibt es keine Studien, die eine Korrelation von vorheriger medizinnaher Berufstätigkeit, sozialem Engagement, Preisen im Bildungsbereich oder sportlichen Leistungen mit sozialen oder kommunikativen Kompetenzen in Studium oder Arztberuf zeigen.

Zur Evaluation der verschiedenen Auswahlverfahren für Studienbewerber in der Humanmedizin sei hier auf einen kürzlich erschienenen Übersichtsartikel verwiesen [6].

Ad iii) Flächendeckende hausärztliche Versorgung

Medizinisch unterversorgte Regionen stellen ein weltweites Problem dar [11], auf das Japan bereits in den 1960er und 70er Jahren mit dem flächendeckenden Ausbau medizinischer Fakultäten – eine für jede der 47 Präfekturen – reagiert hat. Weitere Maßnahmen waren 1972 die Gründung einer Fakultät, in der ausschließlich „Landärzte“ ausgebildet wurden, sowie eine Quotenregelung, die aktuell 17% aller Studienplätze für angehende Landärzte bereitstellt [12]. Die Literatur belegt, dass die Abschlussrate in der Landarztquote mit 96,3% in den Jahren 2014 und 2015 über der Abschlussrate der übrigen Absolventen von 94,2% im gleichen Zeitraum lag und damit keinen Grund für die Annahme liefert, dass durch die Quotenregelung weniger erfolgreiche Studierende zugelassen werden [13]. In Australien wurde seit 2004 ein Viertel der Medizinstudienplätze unter der Auflage, bis zu 22 Jahre in ländlichen Regionen zu arbeiten, vergeben. Umfragen zeigen, dass etwa 25% der Studierenden ein „Herauskaufen“ aus dem Programm nach Studienabschluss erwägen. Weitere Probleme gibt es bei der Definition der ländlichen Regionen, der Facharztwahl der angehenden Ärzte, der Überwachung der Verpflichtung, der Komplexität der Maßnahme und mit einer Stigmatisierung der „Landarztstudierenden“ gegenüber den ungebundenen. Dennoch empfahl ein Regierungsbericht eine Weiterführung des (reformierten) Programms [14]. Über die spätere Verteilung fertiger Ärzte in unterversorgte Regionen liegen jedoch weder in Australien noch in Japan Daten vor. Eine systematische Übersicht der World Health Organization zeigt, dass in über 70 Staaten Programme mit einer Verpflichtung zur Tätigkeit in ländlichen Regionen existieren [15]. Auch wenn systematische Vergleiche zwischen Staaten mit und ohne Verpflichtungsprogrammen noch fehlten, könnten Anreizprogramme dennoch zur Versorgung unterversorgter Gebiete beitragen.

Neben der Einführung einer Quotenregelung gibt der Masterplan das Ziel vor, Studierende auszuwählen, die sich später als Ärzte in unterversorgten Regionen niederlassen. Eine internationale Metaanalyse zu gezielten Maßnahmen, Ärzte in ländlichen und unterversorgten Regionen anzusiedeln, zeigt als einziges Erfolg vorhersagendes Auswahlkriterium für angehende „Landärzte“ die eigene Herkunft vom Land auf [16]. Andere

zielführende Maßnahmen – wie z.B. längere Ausbildungsphasen in unterversorgten Regionen – lagen nicht in der Studierendenauswahl, sondern waren in der Struktur des jeweiligen Curriculums verankert [17].

Zusammenfassend weist der aktuelle Forschungsstand Abiturnote und Eignungstests eine gute prädiktive Validität in Bezug auf den kognitiven Studienerfolg zu. Die Auswahl mittels dieser Kriterien ermöglicht die Umsetzung des 1. Masterplanziels zum hohen Studienerfolg. Die wissenschaftliche Evidenz für den Einsatz von Auswahlverfahren zur sozialen und kommunikativen Kompetenz ist schwächer: Während klassische, eher unstrukturierte Interviews ungeeignet erscheinen, müssen Hinweise auf die Validität von MMI und SJT durch weitere Studien konsolidiert werden. Für die Auswahl angehender Landärzte gibt es jenseits der eigenen ländlichen Herkunft keine Evidenzen dafür, dass irgendein Auswahlkriterium die nachhaltige Niederlassung in unterversorgten Regionen vorhersagt [18]. Eine Auswahl nach „Herkunft vom Land" verbietet jedoch das Grundgesetz. Für die Effizienz einer Landarztquote gibt es keine gesicherte Evidenz.

Die Etablierung, Durchführung und Validierung der im Masterplan geforderten Auswahlverfahren wie z.B. deutschlandweit zentral durchgeführter kognitiver Tests und SJTs oder fakultäre Interviews sind kostenintensiv. Wie im Masterplan vorgesehen, werden die Fakultäten sie nur mit zusätzlicher Unterstützung einsetzen können.

Beigetragen von (alphab.): Wolfgang Hampe, Brigitte Müller-Hilke

Literaturverzeichnis

1. Patterson F, Knight A, Dowell J, Nicholson S, Cousans F, Cleland J. How effective are selection methods in medical education? A systematic review. Med Educ. 2016;50(1):36-60. doi: 10.1111/medu.12817
2. Trapmann S, Hell B, Weigand S, Schuler H. Die Validität von Schulnoten zur Vorhersage des Studienerfolgs-eine Metaanalyse. Z Päd Psychol. 2007;21(1):11-27.
3. Bundesverfassungsgericht. Urteil des Ersten Senats am Bundesverfassungsgericht zum Numerus clausus im Medizinstudium vom 19.12.2017. Karlsruhe: Bundesverfassungsgericht; 2017. Zugänglich unter/available from: https://www.bundesverfassungsgericht.de/SharedDocs/Entscheidungen/DE/2017/12/ls20171219_1bvl000314.html
4. Hell B, Trapmann S, Schuler H. Eine Metaanalyse der Validität von fachspezifischen Studierfähigkeitstests im deutschsprachigen Raum. Emp Pädagogik. 2007;21(3):251-270.
5. Hissbach JC, Klusmann D, Hampe W. Dimensionality and predictive validity of the HAM-Nat, a test of natural sciences for medical school admission. BMC Med Educ. 2011;11(1):83. doi: 10.1186/1472-6920-11-83
6. Schwibbe A, Lackamp J, Knorr M, Hissbach J, Kadmon M, Hampe W. Selection of medical students: Measurement of cognitive abilities and psychosocial competencies. Bundesgesundheitsblatt Gesundheitsforschung Gesundheitsschutz. 2018;61(2):178-186. doi: 10.1007/s00103-017-2670-2
7. Harris BH, Walsh JL, Lammy S. UK medical selection: lottery or meritocracy? Clin Med. 2015;15(1):40-46. doi: 10.7861/clinmedicine.15-1-40
8. Dore KL, Reiter HI, Kreuger S, Norman GR. CASPer, an online pre-interview screen for personal/professional characteristics: prediction of national licensure scores. Adv Health Sci Educ Theory Pract. 2017;22(2):327-336. doi: 10.1007/s10459-016-9739-9

9. Lievens F. Adjusting medical school admission: assessing interpersonal skills using situational judgement tests. Med Educ. 2013;47(2):182-189. doi: 10.1111/medu.12089
10. Patterson F, Cousans F, Edwards H, Rosselli A, Nicholson S, Wright B. The predictive validity of a text-based situational judgment test in undergraduate medical and dental school admissions. Acad Med. 2017;92(9):1250-1253. doi: 10.1097/ACM.0000000000001630
11. WHO. Increasing access to health workers in remote and rural areas through improved retention: Global policy recommendations. Geneva: World Health Organization; 2010. Zugänglich unter/available from: https://www.who.int/hrh/retention/guidelines/en/
12. Matsumoto M, Inoue K, Kajii E, Takeuchi K, Inoue M. Retention of physicians in rural Japan: concerted efforts of the government, prefectures, municipalities and medical schools. Rural Remote Health. 2010;10(2):1432.
13. Matsumoto M, Takeuchi K, Tanaka J, Tazuma S, Inoue K, Owaki T, Iquchi S, Maeda T. Follow-up study of the regional quota system of Japanese medical schools and prefecture scholarship programmes: a study protocol. BMJ Open. 2016;6(4):e011165. doi: 10.1136/bmjopen-2016-011165
14. Mason J. Review of Australian Government Health Workforce Programs. Canberra, Australia: Australian Government, Department of Health; 2013. Zugänglich unter/available from: https://www.health.gov.au/internet/publications/publishing.nsf/Content/work-review-australian-government-health-workforce-programs-toc
15. Frehywot S, Mullan F, Payne PW, Ross H. Compulsory service programmes for recruiting health workers in remote and rural areas: do they work? Bull World Health Organ. 2010;88(5):364-370. doi: 10.2471/BLT.09.071605
16. Reeve C, Woolley T, Ross SJ, Mohammadi L, Halili Jr SB, Cristobal F, Siega-Sur JL, Neusy AJ. The impact of socially-accountable health professional education: a systematic review of the literature. Med Teach. 2017;39(1):67-73. doi: 10.1080/0142159X.2016.1231914
17. Walker JH, Dewitt D, Pallant J, Cunningham C. Rural origin plus a rural clinical school placement is a significant predictor of medical students' intentions to practice rurally: a multi-university study. Rural Remote Health. 2012;12:1908.
18. Grobler L, Marais BJ, Mabunda S. Interventions for increasing the proportion of health professionals practising in rural and other underserved areas. Cochrane database of systematic reviews. 2015;(6):CD005314. doi: 10.1002/14651858.CD005314.pub3
