# Supplementary material for: Position of the advisory and executive board of the German Association for Medical Education (GMA) regarding the “masterplan for medical studies 2020”
Source: GMS J Med Educ. 2019 Aug 15;36(4):Doc46. doi: 10.3205/zma001254 (PMC6737258; doi:10.3205/zma001254)
Supplement: Primary Care Committee [german] [file JME-36-4-46-s-003.pdf]

## Stellungnahme des Ausschusses Primärversorgung

Alle Punkte des Masterplans wurden von den Mitgliedern der Arbeitsgruppe in einem zweistufigen Delphi-Verfahren daraufhin beurteilt, ob sich der Ausschuss Primärversorgung in erster Linie für die jeweiligen Inhalte zuständig fühlt. Identifiziert wurden die Punkte 6, 12, 13, 15, 16, 17.1, 18-23, 34.1, 34.2, 35, 36.1 und 37. Zu diesen Punkten wurde eine Recherche nationaler und internationaler Literatur durchgeführt mit dem Ziel, die geplanten Maßnahmen auf wissenschaftlich belegbare Evidenz hin zu überprüfen.

Die Punkte 6, 16, 17.1 und 23 wurden unter der Fragestellung „Tragen unterschiedliche Formate mit allgemeinmedizinischen Inhalten sowie allgemeinmedizinischer Beteiligung zu einer besseren Sichtbarkeit des Faches bei?“ zusammengefasst. Die Evidenzlage zu dieser Frage ist dürftig und widersprüchlich. Das ist auch die Quintessenz eines internationalen Reviews zu dieser Fragestellung [1]. Wie widersprüchlich die Diskussionslage in Deutschland ist, zeigt sich besonders gut an einer noch aktuellen Diskussion in der Zeitschrift für Allgemeinmedizin [2], [3]. Die Frage, ob unterschiedliche Formate mit allgemeinmedizinischen Inhalten zur besseren Sichtbarkeit des Faches beitragen, wurde direkt/explizit bisher nicht untersucht. Es liegt wenig Evidenz mittelmäßiger bis niedriger Qualität vor zur Institutionalisierung der Allgemeinmedizin und ihrer Sichtbarkeit im Studium.

Es scheint aber offensichtlich - und die vorhandene Literatur stützt diese These, wenn auch auf niedrigem Niveau - dass mehr Beteiligung der Allgemeinmedizin eine bessere Sichtbarkeit mit sich bringt. Ob das zu einer besseren Einstellung gegenüber der hausärztlichen Medizin führt, ist plausibel und wahrscheinlich, aber nicht gesichert [4-8].

Der Punkt 13 des Masterplans stellt eine politische Absichtserklärung dar, aus der sich u.E. keine wissenschaftliche Fragestellung ableiten lässt. Die Punkte 15, 18 und 34.1 wurden unter der Fragestellung „Sind Maßnahmen in der Ausbildung identifizierbar, die zu einer Attraktivitätssteigerung des ländlichen Raumes führen?“ zusammengefasst. Hier finden sich zahlreiche Reviews, vornehmlich aus dem anglo-amerikanischen Raum, die, sehr unterschiedliche und inhomogene Interventionen während der studentischen Ausbildung zusammenfassend, von eher moderaten Effekten im Hinblick auf eine spätere berufliche Tätigkeit im ländlichen Raum berichten [9-11]. Je komplexer und longitudinaler solche Interventionen werden, umso höher ihr Wirkungsgrad [12]. Sehr intensive Programme (z.B. Ausbildungsstätte im ländlichen Raum, Paten-Gemeinde und strikte Ausrichtung des Curriculums auf „Community Medicine“) zeigen Wirkungsgrade von bis zu 64%. Dabei ist allerdings der Faktor ländliche Herkunft nicht herausgerechnet [13]. Für Deutschland existieren einige Einzelstudien, die letztlich eine Motivationssteigerung der Studierenden für die Allgemeinmedizin belegen können, ohne die Karrierewege nach dem Studium weiter verfolgt zu haben [4], [14], [15]. Eine weitere Studie hat zeigen können, dass Ärzte in Weiterbildung, die ein PJ-Tertial Allgemeinmedizin absolviert haben, zu 60% den Facharzt für Allgemeinmedizin anstreben. Hier ist der Faktor ländliche Herkunft allerdings von entscheidender Bedeutung [16].

Die Wirksamkeit einer „Landarztquote“ wurde bislang nicht hinreichend untersucht. Man kann allerdings an dieser Stelle die Frage stellen, ob es eine Evidenz für geeignete Auswahlkriterien für Studierende gibt, die über die Landarztquote zugelassen werden sollen. Ein Cochrane Review belegt, dass ländliche Herkunft der einzig konsistente Prädiktor für eine

spätere Tätigkeit im ländlichen Raum ist, der sich durch nahezu alle Studien hindurchzieht [17]. Zu einer ähnlichen Einschätzung kommt ein WHO-Report [18].

Die Punkte 21 und 22 des Masterplans wurden unter der Frage „Tragen die Institutionalisierung des Faches Allgemeinmedizin und der Ausbau der allgemeinmedizinischen Forschung zu einer Attraktivitätssteigerung des Faches bei?“ zusammengefasst. Es gibt Hinweise, dass eine Institutionalisierung der Allgemeinmedizin (und damit indirekt eine Stärkung der Forschungstätigkeit) für eine positive Einstellung zum Fach und zur Motivation für eine spätere hausärztliche Tätigkeit beiträgt [7].

Die Punkte 12, 19, 20, 24.2, 35 und 36.1 wurden unter der Fragestellung: „Gibt es Hinweise auf sinnvolle flankierende Maßnahmen, die die Allgemeinmedizin bzw. den ländlichen Raum im Studium attraktiver gestalten?“ Die Allgemeinmedizin wird an den Medizinischen Fakultäten in Europa in sehr unterschiedlichem Ausmaß gelehrt [19]. Die Ansätze fokussieren im Wesentlichen auf entweder eine longitudinale Verankerung im Pflichtcurriculum, eine quantitativ und qualitativ hochwertige Lehre und/oder besondere Wahlpflichtangebote [20]. Es gibt Hinweise darauf, dass die frühe und longitudinale Integration der Allgemeinmedizin die Quote allgemeinärztlich tätiger Absolventen erhöht [21], [22].

Eine entscheidende Rolle für die Prägung von Berufswünschen – im ländlichen Bereich und/oder in der Allgemeinmedizin tätig zu werden – spielen positive Rollenmodelle. Eine der Grundvorrausetzungen, um diese zu erleben, sind zeitlich passende, relevante und gut ins Curriculum integriert Praktika [9]. Die Auslagerung von Curriculumsanteilen im Sinne eines dezentralen Trainings hat einen moderaten Effekt auf die landärztliche Tätigkeit von Absolventen in Australien [11] und anderen Ländern [23]. Zu berücksichtigen ist hierbei, dass diese dezentralen Anteile sehr heterogen aufgebaut sind. Gemeinsam ist den Programmen, dass die Studierenden aktiver in die Versorgung eingebunden sind und bessere Lerneffekte bezüglich versorgungsrelevanter Skills erwerben [21]. Entscheidend sind auch hier [9] ein passender curricularer Kontext, eine gute Infrastruktur (z.B. Internetanschlüsse) und eine gute didaktische Betreuung. Die örtlichen Supervisoren müssen adäquat auf die Tätigkeit vorbereitet werden [24]. Studierende sind mit Inhalten und Lernprozessessen in allgemeinmedizinischen Praxisrotationen zufrieden. Diese ergänzen zuvor Gelerntes und ermöglichen eine besondere Lernerfahrung im Umgang mit akuten und chronischen Zuständen, Gesundheitsförderung, Prävention, Kommunikation und spezifischer Problemlösung. Die Einstellungen von Studierenden bezüglich der Allgemeinmedizin verbessert sich, aber dies bleibt nicht zwingend bis zur Berufstätigkeit erhalten [1], [21]. Zur Qualifizierung der vor Ort klinisch tätigen Ausbilder in Hausarztpraxen und Kliniken werden vorbereitende und begleitende Qualifizierungs- Maßnahmen sowohl durch die Fakultäten als auch ggf. durch die Arbeitgeber vor Ort benötigt. Hierzu zählt auch eine finanzielle Unterstützung um genügend Zeit für die Lehre / Ausbildung aufbringen zu können [25].

*Beigetragen von (alphab.): Erika Baum, Klaus Böhme, Maren Ehrhardt, Folkert Fehr, Markus Gulich, Bert Huenges, Irmgard Streitlein-Böhme*

## Literaturverzeichnis

1. Turkeshi E, Michels NR, Hendrickx K, Remmen R. Impact of family medicine clerkships in undergraduate medical education: a systematic review. BMJ Open. 2015;5(8):e008265. doi: 10.1136/bmjopen-2015-008265

2. Rauscher C, Ernst B, Braun M, Schwindl G, Hoffmann H, Kleisch D, Salzberger B. Ausbildung für die regionale Versorgung – das Fach Allgemeinmedizin an der Universität Regensburg. Z Allg Med. 2016;92(9):352-356. doi: 10.3238/zfa.2016.0352–0356
3. Schneider A, Gensichen J, Tauscher M. Zur Notwendigkeit der akademischen Institutionalisierung des Faches Allgemeinmedizin. Z Allg Med. 2017;93(11):456-458. doi: 10.3238/zfa.2017.0456–0458
4. Böhme K, Sachs P, Niebling W, Kotterer A, Maun A. Macht das Blockpraktikum Allgemeinmedizin Lust auf den Hausarztberuf? Eine Analyse studentischer Evaluationen. Z Allg Med. 2016;92(5):220-225. doi: 10.3238/zfa.2016.0220–0225
5. Carney PA, Eliassen MS, Pipas CF, Genereaux SH, Nierenberg DW. Ambulatory care education: how do academic medical centers, affiliated residency teaching sites, and community-based practices compare? Acad Med. 2004;79(1):69-77.
6. O'Sullivan M, Martin J, Murray E. Students' perceptions of the relative advantages and disadvantages of community-based and hospital-based teaching: a qualitative study. Med Educ. 2000;34(8):648-655.
7. Schneider A, Karsch-Völk M, Rupp A, Fischer MR, Drexler H, Schelling J, Berberat P. Determinanten für eine hausärztliche Berufswahl unter Studierenden der Medizin: Eine Umfrage an drei bayerischen Medizinischen Fakultäten. GMS Z Med Ausbild. 2013;30(4):Doc45. doi: 10.3205/zma000888
8. Whitcomb ME. Ambulatory care education: what we know and what we don't. Acad Med. 2002;77(7):591-592.
9. Bunker J, Shadbolt N. Choosing general practice as a career-the influences of education and training. Aust Fam Physician. 2009;38(5):341-344.
10. Curran V, Rourke J. The role of medical education in the recruitment and retention of rural physicians. Med Teach. 2004;26(3):265-272.
11. O'Sullivan BG, McGrail MR, Russell D, Chambers H, Major L. A review of characteristics and outcomes of Australia's undergraduate medical education rural immersion programs. Hum Resour Health. 2018;16(1):8. doi: 10.1186/s12960-018-0271-2
12. Hsueh W, Wilkinson T, Bills J. What evidence-based undergraduate interventions promote rural health? N Z Med J. 2004;117(1204):U1117.
13. Rabinowitz HK, Diamond JJ, Markham FW, Wortman JR. Medical school programs to increase the rural physician supply: a systematic review and projected impact of widespread replication. Acad Med. 2008;83(3):235-243. doi: 10.1097/ACM.0b013e318163789b
14. Holst J, Normann O, Herrmann M. Strengthening training in rural practice in Germany: new approach for undergraduate medical curriculum towards sustaining rural health care. Rural Remote Health. 2015;15(4):3563.
15. Samos FA, Heise M, Fuchs S, Mittmann S, Bauer A, Klement A. Pilot phase evaluation of the elective general practice class: results of student surveys of the first two years. GMS J Med Educ. 2017;34(1)Doc4. doi: 10.3205/zma001081
16. Böhme K, Siegel A, Kotterer A, Streitlein-Böhme I, Maun A. PJ-Wahlfach Allgemeinmedizin - Eine Weichenstellung für die Hausarztkarriere. Z Allg Med. 2018;94(4):179-184.
17. Grobler L, Marais BJ, Mabunda S. Interventions for increasing the proportion of health professionals practising in rural and other underserved areas. Cochrane database of systematic reviews. 2015;(6):CD005314. doi: 10.1002/14651858.CD005314.pub3

18. Dolea C. Increasing access to health workers in remote and rural areas through improved retention: global policy recommendations. Geneva: World Health Organization; 2010.
19. Brekke M, Carelli F, Zarbailov N, Javashvili G, Wilm S, Timonen M, Tandeter H. Undergraduate medical education in general practice/family medicine throughout Europe–a descriptive study. BMC Med Educ. 2013;13(1):157. doi: 10.1186/1472-6920-13-157
20. Blozik E, Ehrhardt M, Scherer M. Förderung des allgemeinmedizinischen Nachwuchses. Bundesgesundheitsblatt Gesundheitsforschung Gesundheitsschutz. 2014;57(7):892-902.
21. Nair M, Fellmeth G. Current efforts in medical education to incorporate national health priorities. Med Educ. 2018;52(1):24-33. doi: 10.1111/medu.13395
22. Tandeter H, Granek-Catarivas M. Choosing primary care? Influences of medical school curricula on career pathways. Isr Med Assoc J. 2001;3(12):969-972.
23. Farmer J, Kenny A, McKinstry C, Huysmans RD. A scoping review of the association between rural medical education and rural practice location. Hum Resour Health. 2015;13(1):27. doi: 10.1186/s12960-015-0017-3
24. De Villiers M, Van Schalkwyk S, Blitz J, Couper I, Moodley K, Talib Z, Young T. Decentralised training for medical students: a scoping review. BMC Med Educ. 2017;17(1):196. doi: 10.1186/s12909-017-1050-9
25. von Below B, Hellquist G, Rödjer S, Gunnarsson R, Björkelund C, Wahlqvist M. Medical students' and facilitators' experiences of an Early Professional Contact course: Active and motivated students, strained facilitators. BMC Med Educ. 2008;8(1):56. doi: 10.1186/1472-6920-8-56
