# Supplementary material for: Position of the advisory and executive board of the German Association for Medical Education (GMA) regarding the “masterplan for medical studies 2020”
Source: GMS J Med Educ. 2019 Aug 15;36(4):Doc46. doi: 10.3205/zma001254 (PMC6737258; doi:10.3205/zma001254)
Supplement: Practical Skills Committee [german] [file JME-36-4-46-s-004.pdf]

Stellungnahme des Ausschusses Praktische Fertigkeiten

Den Praktischen Fertigkeiten im Medizinstudium kommt im Masterplan Medizinstudium 2020 ein hoher Stellenwert zu. So steht bereits im zweiten Satz der Pressemitteilung des Bundesministeriums für Bildung und Forschung [1] dazu *„Die Lehre wird an der Vermittlung arztbezogener Fähigkeiten ausgerichtet. Dabei gilt das besondere Augenmerk dem Arzt-Patienten-Gespräch, das maßgeblich die Arzt-Patienten-Beziehung, den Behandlungserfolg und das Wohlbefinden der Patientinnen und Patienten beeinflusst.“*

Dies wird vom Ausschuss für Praktische Fertigkeiten als Auftrag gesehen, die primär am Patienten ausgeübten Fertigkeiten möglichst praxisnah zu üben und die erwähnten Fertigkeiten zunächst in simulierten Settings erwerben und dann am Patientenbett zu festigen. Im Vordergrund steht dabei insbesondere die Patientensicherheit.

Auch im Beschlusstext [1] kommt den praktischen Fertigkeiten eine hohe Bedeutung zu. Die explizite Erwähnung von OSCE als nationale Prüfungsform wird vom Ausschuss ausdrücklich begrüßt und geht mit der Konsequenz einher, dass die räumliche, sächliche und personelle Ausstattung der Skillslabs, die mittlerweile flächendeckend in den medizinischen Fakultäten vorhanden sind [2], [3], entsprechend angepasst werden muss. Dies stellt die Fakultäten sicher vor einige Herausforderungen, die nach Einschätzung des Ausschusses aber gemeistert werden können. Auch die Ankündigung, dass der NKLM [4] als verbindlicher Teil der Approbationsordnung angesehen wird, wird ausdrücklich begrüßt, da der NKLM in den Kapiteln 14b (Klinisch-praktische Fertigkeiten), 14c (Ärztliche Gesprächsführung) und 17 (Notfallmedizin) weitgehend dem Positionspapier des Ausschusses für Praktische Fertigkeiten entspricht [5]. Auch in der Zukunft wird der Ausschuss für Praktische Fertigkeiten sich an der Entwicklung des NKLM beteiligen.

Die Stärkung der kommunikativen Fertigkeiten wird vom Ausschuss begrüßt, da er nach Einschätzung des Ausschusses die Patientensicherheit erhöhen wird [6]. Auch wenn der Umfang sicher noch zu diskutieren ist (ein nationales Mustercurriculum ist zu undifferenziert), werden die praktischen Prüfungen erhebliche Anforderungen an die räumliche, sächliche und personell-qualifizierte Ausstattung bzw. Bereitstellung der Fakultäten stellen. Die Verknüpfung klinischer und theoretischer Lerninhalte ab dem ersten Studienjahr bedingt aus Sicht des Ausschusses eine Stärkung des simulationsbasierten Unterrichts in Skills Labs schon in den ersten Jahren, um die Studierenden auf den Patientenkontakt angemessen vorbereiten zu können.

Als Manko sieht es der Ausschuss an, dass die Simulationssettings, die die Patientensicherheit erhöhen, weil sie nachweislich zu besseren Ergebnissen als das konventionelle Bedsideteaching führen [7] nicht expliziter erwähnt sind. Genauer sollten diese als „Unterricht am Krankenbett“ definiert sein, um eine adäquate Dozierenden-Studierenden-Relation zu erreichen. Hier könnte eine Win-Win-Situation durch den Einbezug moderner Ausbildungsstätten mit Skills Labs ressourcensparend für die Medizinischen Fakultäten entstehen, die über die Ausbildung hinaus wirksam ist („deliberate practice“ wirkt auch in der Weiter- und Fortbildung). Zudem weist der Entwurf Masterplan Medizinstudium 2020 nicht einen Hinweis auf interprofessionelle Ausbildung aller Gesundheitsberufe auf und verkennt damit erheblich das Potential, welches insbesondere im gemeinsamen Tun – also der Ausübung patientennaher Fertigkeiten – trainiert und reflektiert werden kann.

Der Ausschuss sieht es auch als kritisch an, dass keine Übergangsfristen avisiert sind, die den Fakultäten die Möglichkeit geben könnten, sich frühzeitig auf die stark gestiegenen Anforderungen an die Skillslabs einzustellen.

Insgesamt weist der Masterplan 2020 in die richtige Richtung und wird vom APF begrüßt.

Beigetragen von (alphab.): Kai Schnabel, Christoph Stosch

Literaturverzeichnis

1. Bundesministerium für Bildung und Forschung. Masterplan Medizinstudium 2020. Berlin: Bundesministerium für Bildung und Forschung; 2017. Zugänglich unter/available from: https://www.bmbf.de/de/masterplan-medizinstudium-2020-4024.html
2. Damanakis A. Statusreport von Skills Labs in der D-A-CH-Region und Aufbau einer Informationsplattform zur Katalogisierung und Bewertung von Simulatoren zur medizinischen Ausbildung. Marburg: Philipps-Universität-Marburg; 2015.
3. Stosch C, Schnabel KP. Didactic, practical, good! 20 years of clinical skills training in the German speaking countries. GMS J Med Educ. 2016;33(4):Doc67. doi: 10.3205/zma001066
4. Hickel R, Fischer M. Stand der nationalen kompetenzbasierten Lernzielkataloge Medizin (NKLM) und Zahnmedizin (NKLZ). Halle/Berlin: Medizinischer Fakultätentag; 2013.
5. Schnabel K, Boldt PD, Breuer G, Fichtner A, Karsten G, Kujumdshiev S, Schmidts M, Stosch C. Konsensusstatement „Praktische Fertigkeiten im Medizinstudium "-ein Positionspapier des GMA-Ausschusses für praktische Fertigkeiten. GMS Z Med Ausbild. 2011;28(4):Doc58. doi: 10.3205/zma000770
6. Tamblyn R, Abrahamowicz M, Dauphinee D, Wenghofer E, Jacques A, Klass D, Smee E, Blackmore D, Winslade N, Girad N, Du Berger R, Bartman I, Buckeridge DL, Hanley JA. Physician scores on a national clinical skills examination as predictors of complaints to medical regulatory authorities. JAMA. 2007;298(9):993-1001.
7. McGaghie WC, Issenberg SB, Cohen MER, Barsuk JH, Wayne DB. Does simulation-based medical education with deliberate practice yield better results than traditional clinical education? A meta-analytic comparative review of the evidence. Acad Med. 2011;86(6):706-711. doi: 10.1097/ACM.0b013e318217e119
