# Supplementary material for: Position of the advisory and executive board of the German Association for Medical Education (GMA) regarding the “masterplan for medical studies 2020”
Source: GMS J Med Educ. 2019 Aug 15;36(4):Doc46. doi: 10.3205/zma001254 (PMC6737258; doi:10.3205/zma001254)
Supplement: Communicative and Social Competences Committee [german] [file JME-36-4-46-s-005.pdf]

Stellungnahme des Ausschusses Kommunikative und soziale Kompetenzen

Der Implementierung sozialer und kommunikativer Kompetenzen in das Medizinstudium kommt im Masterplan Medizinstudium 2020 ein hoher Stellenwert zu. Der Ausschuss Kommunikative und soziale Kompetenzen der GMA begrüßt diese Entwicklung ausdrücklich, insbesondere das Ziel, dass longitudinale Kommunikationscurricula an allen Medizinischen Fakultäten Eingang finden. Neben der Arzt-Patienten-Kommunikation ist die Erweiterung kommunikativer und sozialer Kompetenzen um Aspekte der inter- und intraprofessionellen sowie der multidisziplinären und -professionellen Kommunikation zeitgemäß [1], [2].

Im Masterplan Medizinstudium 2020 wird die Umsetzung der Vorgaben basierend auf dem „Nationalen Mustercurriculum Kommunikation (LongKomm)“ [3] angestrebt. Dieses Mustercurriculum umfasst insgesamt 450 Unterrichtseinheiten (UE) (davon 350 UE als Pflichtveranstaltungen und 100 UE als Wahlpflichtveranstaltungen). Es sieht darüber hinaus vor, kommunikative Lehrinhalte auch im klinischen Unterricht zu integrieren. In der Regel umfassen die medizinischen Curricula an deutschen Fakultäten 5500 Stunden Lehre (vgl. §1(2) ÄAppO [https://www.gesetze-im-internet.de/_appro_2002/BJNR240500002.html], sowie Abschnitt 2 Artikel 24 (2)) RL 2005/36/EC, Abschnitt 2, Artikel 24 (2) [4]]. Eine Erweiterung dieses Lehrumfangs ist im Masterplan Medizinstudium 2020 nicht vorgesehen. Dies bedeutet, dass von 5500 Stunden über 8 Prozent der Pflicht- und Wahlpflichtlehre für die curriculare Vermittlung kommunikativer und sozialer Kompetenzen – ohne integrierte Veranstaltungen – veranschlagt werden. Im Vergleich zu z.B. allen Fächern der Vorklinik, die laut geltender Approbationsordnung mindestens 630 Stunden umfassen sollen, erscheint der Stundenumfang unverhältnismäßig hoch.

Der Nationale Kompetenzbasierte Lernzielkatalog Medizin [http://www.nklm.de] umfasst insgesamt 18 Kompetenz- und Lernzielbereiche und soll als Orientierung für die curriculare Gestaltung an den Fakultäten dienen. Im NKLM findet sich jedoch keine Angabe zu zeitlichen Unterrichtsumfängen der einzelnen Bereiche. Es ist nicht nachvollziehbar, warum dies nun für einen einzelnen Kompetenzbereich vorgenommen und wie dieser hohe Stundenumfang begründet wird. Eine Evidenz für diesen spezifischen Vorschlag im Masterplan Medizinstudium 2020 ist nicht gegeben.

Im Jahr 2015 hatte bereits ein Drittel aller medizinischen Fakultäten im deutschsprachigen Raum (Deutschland, Österreich, Schweiz; sog. DACH-Region) ein eigenes longitudinales Kommunikationscurriculum implementiert [6]. Zwar basieren viele Bestandteile des Mustercurriculums „LongKomm“ auf Erfahrungen und Best-Practice-Beispielen verschiedener Kommunikationscurricula an den Standorten der DACH-Region, jedoch ist eine verbindliche Vorgabe für ein Teilcurriculum für alle Fakultäten aus verschiedenen Gründen nicht sinnvoll. Zunächst finden sich im Masterplan Medizinstudium 2020 selbst Widerspruche, da einerseits ein national einheitliches Kommunikationscurriculum umgesetzt werden soll und andererseits die Fakultäten aufgefordert sind, Innovationen im Lehren und Lernen voran zu treiben. Weiterhin fällt auf, dass für diese angestrebte Implementierung der kommunikativen Lehr- und Lernziele bzw. eines longitudinalen Kommunikationscurriculums das Mustercurriculum „LongKomm“ prioritär vorgeschlagen wird. Diese Betonung wird im Masterplan Medizinstudium 2020 nicht begründet und ist daher nicht nachvollziehbar. Zum einen bestehen bereits Kommunikationscurricula, die beständig evaluiert und weiterentwickelt werden [5-8]. Zum anderen fehlen in den Ausführungen des Masterplan

Medizinstudium 2020 evidenzbasierte Gründe, um die Implementierung eines national einheitlichen Curriculums für alle Fakultäten, unabhängig von den eigenen Curriculumsinhalten und der Curriculumsgestaltung bzw. den -schwerpunkten und der Art des Curriculums (Regel- oder Modellstudiengang), nachvollziehbar zu rechtfertigen.

Darüber hinaus bleibt offen, inwieweit der Konzeptions- bzw. Entstehungsprozess des „LongKomm" wissenschaftlich fundiert erfolgt ist oder ob es vielmehr ein Meinungsbild einer Personengruppe darstellt. Zudem lässt das „LongKomm" selbst wissenschaftliche Evidenzen für ein qualitatives Alleinstellungsmerkmal vermissen.

Unabhängig von der Art des Kommunikationscurriculums erfordert die Implementierung bzw. der Ausbau der Lehre in diesem Bereich neben der Umstrukturierung sowohl für die Lehre als auch für die geforderten Prüfungen entsprechend qualifizierte Lehrende. Hierfür werden weder Ressourcen noch Strategien in den entsprechenden Maßnahmen thematisiert. Zudem umfasst der Masterplan Medizinstudium 2020 viele Maßnahmen, die scheinbar verschiedene Bereiche thematisieren, jedoch im Sinne eines integrierten Curriculums gemeinsam betrachtet werden sollen. So sind z.B. ambulantes Arbeiten, kommunikative und soziale Kompetenzen und Wissenschaftlichkeit keine getrennten Bereiche. Auch das Thema Prüfungen sollte nicht unabhängig vom Kontext betrachtet werden.

Wir begrüßen die deutliche Aufforderung, an allen Medizinischen Fakultäten longitudinale Kommunikationscurricula zu implementieren. Die Konzeption und Umsetzung dieser muss jedoch den jeweiligen Hochschulen überlassen werden, da diese die fachliche Expertise haben. Hier würde von Seiten der Politik ein Eingriff in die Autonomie der Hochschulen vorgenommen, der aus wissenschaftlicher Sicht nicht hinnehmbar wäre.

Elemente, die bereits an einzelnen Medizinischen Fakultäten etabliert sind, sollten nach eigenem Ermessen erhalten bleiben bzw. weiterentwickelt werden können. Daher ist insbesondere die Maßnahme der Einführung eines nationalen einheitlichen Kommunikationscurriculums an allen Medizinischen Fakultäten abzulehnen.

Beigetragen von (alphab.): Cadja Bachmann, Julia Freytag, Anja Härtl, Linn Hempel, Tanja Hitzblech, André Karger, Gudrun Karsten, Rolf Kienle, Claudia Kiessling, Tim Peters, Swetlana Philipp, Katrin Rockenbauch, Kai Schnabel, Andrea Schönbauer, Michael Sommer, Tina Stibane, Anja Zimmermann

Literaturverzeichnis

1. Bachmann C, Abramovitch H, Barbu CG, Cavaco AM, Elorza RD, Haak R, Loureiro E, Ratasjka A, Silverman J, Winterburn S, Rosenbaum M. A European consensus on learning objectives for a core communication curriculum in health care professions. Patient Educ Couns. 2013;93(1):18-26. doi: 10.1016/j.pec.2012.10.016
2. Von Fragstein M, Silverman J, Cushing A, Quilligan S, Salisbury H, Wiskin C; UK Council fo rClinical Communication Skills Teaching in Undergraduate Medical Eduation. UK consensus statement on the content of communication curricula in undergraduate medical education. Med Educ. 2008;42(11):1100-1107. doi: 10.1111/j.1365-2923.2008.03137.x

3. Jünger J, Mutschler A, Kröll K, Weiss C, Fellmer-Drügg E, Köllner V, Ringel N. Ärztliche Gesprächsführung in der medizinischen Aus-und Weiterbildung-Das Nationale longitudinale Mustercurriculum Kommunikation. Med Welt. 2015;66:189-192.
4. Europäische Union. Richtlinie 2005/36/EG des Europäischen Parlaments und des Rates vom 7. September 2005 über die Anerkennung von Berufsqualifikationen. Brüssel: Europäische Union; 2005.
5. Härtl A, Bachmann C, Blum K, Höfer S, Peters T, Preusche I,Raski B, Rüttermann S, Wagner-Menghin M, Wünsch A, Kiessling C; GMA-Ausschuss Kommunikative und Soziale Kompetenzen . Desire and reality–teaching and assessing communicative competencies in undergraduate medical education in German-speaking Europe–a survey. GMS Z Med Ausbild. 2015;32(5):Doc56. doi: 10.3205/zma000998
6. Kienle R, Arends P, Beck S, Dettmer S, Hölzer H, Sonntag U, Steinbart D, Peters H. Evaluation des longitudinalen und integrierten Curriculums der Lehrveranstaltung „Kommunikation, Interkation, Teamarbeit“. In: Jahrestagung der Gesellschaft für Medizinische Ausbildung (GMA); 25.09.-27.09.2014; Hamburg. Düsseldorf: German Medical Science GMS Publishing House; 2014. DocP433. doi: 10.3205/14gma161
7. Karger A, Hempel L. Wie angehende Ärzte medizinische Kommunikation lernen: Die Vermittlung kommunikativer Kompetenz im Medizinstudium am Beispiel von CoMeD. In: Bechmann S, ed. Sprache und Medizin: Interdisziplinäre Beiträge zur medizinischen Sprache und Kommunikation. Forum für Fachsprachen-Forschung. 138. Berlin: Frank & Timme; 2017. p.401-418.
8. Terzioglu P, Jonitz B, Schwantes U, Burger W. Kommunikative und soziale Kompetenzen-Vermittlung muss im Medizinstudium beginnen. Dtsch Ärztebl. 2003;100(36):2277-2278.
