# Supplementary material for: Position of the advisory and executive board of the German Association for Medical Education (GMA) regarding the “masterplan for medical studies 2020”
Source: GMS J Med Educ. 2019 Aug 15;36(4):Doc46. doi: 10.3205/zma001254 (PMC6737258; doi:10.3205/zma001254)
Supplement: Standardized Patients Committee [german] [file JME-36-4-46-s-006.pdf]

Stellungnahme des Ausschusses Simulationspatienten

Als Ausschuss begrüßen wir die im Masterplan Medizinstudium 2020 geforderte Ausweitung praktischer Anteile, wie z.B. Simulationen im Lehr- und Prüfungssektor, den frühen und kontinuierlichen Einsatz dieser Methoden sowie ihre stärkere Gewichtung in der Vermittlung von kommunikativen, sozialen und interprofessionellen Kompetenzen. Insbesondere die Stärkung der Arzt-Patient-Kommunikation halten wir ebenfalls für ausgesprochen wichtig.

Solche Kompetenzen können nur unzureichend rein theoretisch gelehrt und schriftlich geprüft werden. Sie bedürfen einer Unterfütterung durch praktischen Unterricht und praktische Prüfungen. Als effektive Methode hat sich dabei der Einsatz von Simulationspatienten (SP) bewährt. Auch für praxisorientierte Auswahlverfahren als zusätzliche Kriterien neben der Abiturnote sind Verfahren unter Einbezug von SPs denkbar und an einigen Standorten bereits in Erprobung.

Die Fakultäten haben in den letzten Jahren bereits SP-Programme etabliert, die die Vermittlung der geforderten Kompetenzen unterstützen, indem sie beispielsweise die praxisorientierte Ausbildung in Kleingruppen ausgebaut haben. Über das Training kommunikativer Kompetenzen hinaus werden mit SPs auch weitere praktische Fertigkeiten und Haltungen früh und kontinuierlich im Laufe der Curricula trainiert. Diese Bemühungen stehen im Einklang mit den Zielen des Masterplan Medizinstudium 2020.

Abgesehen von der inhaltlichen Befürwortung, halten wir es jedoch für unabdingbar, den einzelnen Fakultäten hierbei Gestaltungsmöglichkeiten zu überlassen, um ein Longitudinales Kommunikations-Curriculum dem jeweiligen Standort und dem dort bestehenden Curriculum anpassen zu können.

Durch die mit dem Masterplan Medizinstudium 2020 angestrebten Entwicklungen wird die Methode des Einsatzes von SPs an Bedeutung in Lehre und Prüfung gewinnen.

Wenn in den verschiedenen oben genannten Formaten SPs eingesetzt werden, dann müssen dabei die Qualitätskriterien eingehalten werden, die sowohl national [1] als auch international [2], [3] formuliert wurden. Hinter diese Standards darf gerade bei einer massiven Erweiterung von SP-Einsätzen und deren Integration in high-stakes-Prüfungen nicht zurückgefallen werden; im Gegenteil gilt es, sie mit Leben zu füllen und konstruktiv weiterzuentwickeln. Bezüglich der Einführung von OSCEs als Teile der Staatsexamina sind klare Standards für die Durchführung – unter anderem für die Konzeption von Prüfungsstationen und die Qualifizierung der Prüferinnen und Prüfer – unabdingbar. Wir fordern in diesem Zusammenhang, dass bei der Entwicklung dieser Standards die Fakultäten einbezogen werden und die dort bereits vorhandene Expertise zur Durchführung von OSCEs genutzt wird.

Auch in etwaigen Auswahlverfahren mithilfe von Simulationen müssen die gleichen hohen Standards wie bei Prüfungen gelten - mit den entsprechenden Konsequenzen für Ressourcen und Qualitätssicherung.

Die Fakultäten haben beim Einsatz von SPs eine große Expertise entwickelt und Erfahrungen gesammelt. Diese gilt es gezielt weiterzuentwickeln und zu fördern. Die wissenschaftliche Erforschung der Methode muss weiter vorangebracht werden. Gerade der angestrebte Einsatz von SPs in der Ärztlichen Prüfung stellt einen Entwicklungssprung dar. Die Anforderungen an die Qualität der Darstellung z.B. in Bezug auf Standardisierung müssen der Bedeutung der Ärztlichen Prüfung für den Berufsstart gerecht werden. Die Bedingungen

unter denen dies im deutschen Ausbildungssystem mit einer so großen Zahl von SPs, die nicht gemeinsam trainiert werden können, gelingen kann, müssen erst noch erforscht werden.

Die im Masterplan geforderte Ausweitung der Lehre in diesem Bereich und die zusätzlichen Prüfungen stellen einen Aufgabenzuwachs dar, der mit den vorhanden personellen und materiellen Ressourcen nicht bewältigt werden kann. Daher müssen die Fakultäten mit den entsprechenden finanziellen Mitteln ausgestattet werden, um dies zu leisten. Dies beinhaltet Mittel für:

- Eine angemessene Vergütung einer ausreichenden Zahl von SPs,
- Didaktisch qualifiziertes Personal für die Rekrutierung, Schulung der SPs sowie Lehr- und Prüfungspersonal,
- Personal für die Entwicklung longitudinaler Curricula,
- Lehrbegleitforschung,
- Fortlaufende Qualifizierungsmaßnahmen,
- Räumliche und weitere materielle Ressourcen.

Beigetragen von (alphab.): Anke Adelt, Julia Freytag, Angelika Hiroko Fritz, Tim Peters, Renate Strohmer, Christian Thrien

Literaturverzeichnis

1. Peter T, Thrien C. Simulationspatienten: Handbuch für die Aus- und Weiterbildung in medizinischen und Gesundheitsberufen. Bern: Hogrefe; 2018.
2. Cleland JA, Abe K, Rethans JJ. The use of simulated patients in medical education: AMEE Guide No 42. Med Teach. 2009;31(6):477-486.
3. Lewis KL, Bohnert CA, Gammon WL, Hölzer H, Lyman L, Smith C, Thompson TM, Wallace A, Gilva-McConvey G. The association of standardized patient educators (ASPE) standards of best practice (SOBP). Adv Simul (London). 2017;2(1):10. doi: 10.1186/s41077-017-0043-4
