# Supplementary material for: Position of the advisory and executive board of the German Association for Medical Education (GMA) regarding the “masterplan for medical studies 2020”
Source: GMS J Med Educ. 2019 Aug 15;36(4):Doc46. doi: 10.3205/zma001254 (PMC6737258; doi:10.3205/zma001254)
Supplement: Interprofessional Education Committee [german] [file JME-36-4-46-s-007.pdf]

Stellungnahme des Ausschusses Interprofessionelle Ausbildung

Der Ausschuss für interprofessionelle Ausbildung begrüsst grundsätzlich den Masterplan Medizinstudium 2020. Die zukünftige PatientInnenversorgung wird mehr und mehr als Teamarbeit verschiedener Gesundheitsberufe erfolgen müssen, und auf diese notwendige Zusammenarbeit muss das Medizinstudium besser vorbereiten. Die Evidenz interprofessioneller Ausbildung auf die spätere Zusammenarbeit ist belegt [1]. Die Zusammenarbeit mit anderen Gesundheitsberufen wird im Masterplan Medizinstudium 2020 zumindest auf den Seiten 3, 4 und 5 erwähnt, der Terminus „multiprofessionell" findet sich (S. 4), „interprofessionell" fehlt jedoch.

Somit wird interprofessionelles Arbeiten nicht als notwendiger Bestandteil des „neuen" Curriculums benannt, sondern eher umschrieben. Der Ausschuss für Interprofessionelle Ausbildung befürchtet, dass der Aspekt der Zusammenarbeit und des gemeinsamen Lernens mit anderen Gesundheitsberufen somit nicht explizit genug betrachtet wird. Reeves et al. [1] definieren interprofessionelle Ausbildung: „*…as an intervention where the members of more than one health or social care profession, or both, learn interactively together, for the explicit purpose of improving interprofessional collaboration or the health/well being of patients/clients, or both.*" Zur besseren Darstellung sollte sich der Masterplan Medizinstudium 2020 auf „interprofessionelle Ausbildung" beziehen.

Um die im Masterplan Medizinstudium 2020 formulierte „multiprofessionelle Arbeit in Teams […] in der gemeinsamen Ausbildung" umsetzen zu können [2] sind sowohl interprofessionell kompetente Ärztinnen und Ärzte als Lehrende, aber auch Lehrende (und Auszubildende) anderer Gesundheitsberufe in Pflege, Therapie und Diagnostik gefordert. Dazu müssen interprofessionelle didaktische Aus- und Weiterbildungsformate geschaffen werden.

Um Nachhaltigkeit zu erzeugen, müssen im Sinne des *constructive alignments* auch interprofessionelle Prüfungen für Studierende (wie z. B. Team- OSCE [3]) etabliert werden. Im Sinne der Kompetenzorientierung müssten dafür zunächst interprofessionelle Kompetenzen formuliert werden. Hier bietet sich der Zugriff auf Kompetenzrahmen an.

Grundsätzlich bieten sich interprofessionelle Lehrformate auch für fast alle genannten Inhalte des Masterplan Medizinstudium 2020 an: kommunikative Kompetenzen, wissenschaftliche Kompetenzen, aber auch die Themen Grundversorgung und Digitalisierung.

Die Ausbildungen von Medizinstudierenden aber auch von anderen Gesundheitsfachberufen sollten zukünftig stets auch interprofessionelle Curricula beinhalten, d.h. eine longitudinale Dimension aufweisen. Ähnlich wie der Ausschuss Kommunikative und soziale Kompetenzen empfiehlt, sollte es kein deutschlandweites Mustercurriculum geben, sondern es sollte eher an den Erfahrungen der medizinischen Fakultäten mit interprofessionellen Modulen angedockt werden (vgl. z. B. https://www.bosch-stiftung.de/de/projekt/operation-team-interprofessionelles-lernen).

Letztlich muss die Kapazitätsverordnung nicht-ärztliche Lehrkräfte im Pflichtcurriculum ermöglichen, ohne die Aufnahmekapazitäten der jeweiligen Fakultät zu beeinflussen. Nur so können verbindliche (Wahl-)Pflichtveranstaltungen in die Curricula implementiert werden. Interprofessionelle Ausbildungsstationen im praktischen Ausbildungsabschnitt umgehen

diese Thematik, müssen aber im Sinne eines Curriculums mit theoretischen Lehrveranstaltungen verknüpft sein.

Konkrete Forderungen des Ausschusses für Interprofessionelle Ausbildung sind:

- Interprofessionelle Ausbildung als Lernen voneinander, miteinander und übereinander zu verstehen
- Verbindliche, möglichst verpflichtende und longitudinale Integration von Interprofessionalität ins medizinische Curriculum
- Ausloten von Modellen für die erfolgreiche Integration interprofessioneller Lehrveranstaltungen in die Curricula der beteiligten Gesundheitsberufe
- Entwicklung von Aus- und Weiterbildungen für interprofessionell Lehrende
- Definition interprofessioneller Kompetenzen unter Verwendung von Kompetenzrahmen
- Entwicklung von Prüfungen interprofessioneller Kompetenzen
- Entwicklung interprofessioneller Evaluationsformaten
- Qualitätssicherung und -entwicklung der PatientInnenversorgung steht im Mittelpunkt aller interprofessionellen Ausbildungen.

Beigetragen von (alphab.): Sebastian Bode, Marion Huber, Sylvia Kaap-Fröhlich, Birgit Wershofen

Literaturverzeichnis

1. Reeves S, Perrier L, Goldman J, Freeth D, Zwarenstein M. Interprofessional education: effects on professional practice and healthcare outcomes (update). Cochrane Database Syst Rev. 2013;3(3):CD002213. doi: 10.1002/14651858.CD002213.pub3
2. Bundesministerium für Bildung und Forschung. Masterplan Medizinstudium 2020. Berlin: Bundesministerium für Bildung und Forschung; 2017. Zugänglich unter/available from: https://www.bmbf.de/files/2017-03-31_Masterplan%20Beschlusstext.pdf
3. Singleton A, Smith F, Harris T, Ross-Harper R, Hilton S. An evaluation of the team objective structured clinical examination (TOSCE). Med Educ. 1999;33(1):34-41.
