# Supplementary material for: Position of the advisory and executive board of the German Association for Medical Education (GMA) regarding the “masterplan for medical studies 2020”
Source: GMS J Med Educ. 2019 Aug 15;36(4):Doc46. doi: 10.3205/zma001254 (PMC6737258; doi:10.3205/zma001254)
Supplement: Integrative Medicine and Perspective Pluralism Committee [german] [file JME-36-4-46-s-008.pdf]

Stellungnahme des Ausschusses Integrative Medizin und Perspektivenpluralismus

Der Ausschuss Integrative Medizin und Perspektivenpluralismus begrüßt und befürwortet die im Masterplan Medizinstudium 2020 entwickelte frühzeitige und konsequente Orientierung des Studiums der Humanmedizin an Patienten und ihren Bedürfnissen ausdrücklich. Im Folgenden werden Schwerpunktsetzungen zur Gestaltung des Masterplans entwickelt und begründet; abschließend werden konkrete Maßnahmen aufgelistet.

Die Schwerpunktsetzungen betreffen

- Anthropologische Konzepte und Auseinandersetzung mit verschiedenen Modellen zu Gesundheit und Krankheitsverarbeitung
- Früh im Studium beginnende, didaktisch strukturierte Begegnungen mit Patienten und aktive Partizipation in der Patientenversorgung
- Berücksichtigung der grundlegenden Prinzipien einer Evidenz-basierten Medizin incl. der Anliegen der Patienten
- Integrative Medizin und Auseinandersetzung mit der Perspektivenpluralität vorhandener Ansätze in Wissenschaft und Gesundheitsversorgung
- Ärztliche Selbstfürsorge und -regulation als Bestandteil professioneller Persönlichkeitsentwicklung.

Anthropologische Konzepte und Auseinandersetzung mit verschiedenen Modellen zu Gesundheit und Krankheitsverarbeitung

Für ein umfassendes Verständnis von Patienten und ihren Bedürfnissen ist die Auseinandersetzung mit und Diskussion von anthropologischen Grundkonzepten im Medizinstudium erforderlich [1]. Ohne dies droht die Gefahr einer unbewussten und unreflektierten Zugrundelegung eines antiquierten mechanistischen Erklärungsmodells des Menschen, das sich aus einem reduktionistischen Wissenschaftsparadigma speist. In diesem Zusammenhang ist zudem eine intensivierte Auseinandersetzung mit verschiedenen Modellen von Gesundheit (vgl. u.a. [2], [3]) sowie Krankheitsverarbeitung (z.B. [4]) als Grundlegung für ein vertieftes Verstehen der Genese von Gesundheit und Krankheit sowie des Befindens und der Sicht des Patienten zu empfehlen.

Früh im Studium beginnende, didaktisch strukturierte Begegnungen mit Patienten und aktive Partizipation in der Patientenversorgung

Ärztliche Praxis, diagnostisches Erkennen und therapeutisches Handeln nehmen ihren Ausgang vom Hilfeersuchen individueller Personen, die gesundheitsbedingt in eine Notsituation geraten sind [5]. Um hierzu qualifiziert auszubilden, sind insbesondere didaktisch gut gestaltete Situationen resp. Lernumgebungen longitudinal im Studienverlauf zu schaffen, in denen Studierende realen und Simulationspatienten begegnen und deren komplexe Krankheits- und mehrdimensionale Lebenssituation sowie individuelle Bedürfnislage, einschließlich Sinnsuche, mit genügend Zeit erfassen lernen.

Dies kann und sollte bereits früh im Studium beispielsweise in geführten Interviews oder Explorationen von Patienten zu deren Erlebnissen im Gesundheitswesen, in z.B. Falltagen mit realen Patienten [6] oder in ähnlich konzipierten Veranstaltungen mit Simulationspatienten erfolgen. Mit zunehmendem Studienverlauf erhält die aktive Teilhabe respektive supportierte Mitarbeit von Studierenden bis hin zur eigenständigen

Patientenversorgung durch Studierende einen wichtigen Stellenwert [7]. Blockpraktika und Praktisches Jahr sind entsprechend didaktisch gut zu strukturieren und mit Reflexionsangeboten auszustatten [8]. Als Modell zur entsprechenden Umsetzung im PJ wurden Ausbildungsstationen entwickelt: Studierende werden in das klinische, multiprofessionelle Versorgungsteam integriert; sie übernehmen als studentisches Team sämtliche ärztliche Aufgaben auf der Station, werden von Lehrärzten supervidiert und unterrichtet und erhalten Support seitens des multiprofessionellen Teams [9]. Zudem werden sie durch geführte Reflexion im Rahmen eines klinischen Reflexionstrainings in ihrer professionellen Entwicklung unterstützt [10]. In einem solchen Setting tragen die Studierenden zu einer hohen Patientenorientierung der Patientenversorgung bei [11].

Mit einer sich durch das gesamte Studium erstreckenden systematischen Ausbildung können Entrustable Professional Activities (EPAs) stufenweise eingeübt und überprüft werden (vgl. [12], [13]); die Kompetenzen für die Gestaltung einer vertrauensbildenden Patienten-Arzt-Beziehung können solchermaßen intensiviert vermittelt werden.

Vollständige Berücksichtigung der Prinzipien einer Evidenz-basierten Medizin incl. der Anliegen der Patienten und Befähigung zum wissenschaftlichen Arbeiten

Die Begegnung mit Patienten in geeigneten Settings bietet ein praktisches Lern- und Anwendungsfeld auch für das „Grundlagenwissen über (…) die geistig-seelischen Eigenschaften des Menschen“ (§ 1 ÄApprO, Abs. 1, Satz 4) bzw. dessen individuelle Konkretisierung. Dies kann in seiner Bedeutung für das Erlernen und spätere Realisieren einer Evidenz-basierten Medizin (EBM) kaum hoch genug eingeschätzt werden. Hierbei ist darauf zu achten, dass bei Vermittlung von EBM nicht nur die externe Evidenz gewürdigt wird. Die Praxis der EBM ist definiert als die individuelle klinische Expertise *inklusive* der sorgfältigen Identifikation und anteilnehmenden Berücksichtigung der individuellen Notlage, Rechte und Präferenzen des Patienten, welche für die klinische Urteilsbildung in Diagnostik und Therapie in Verknüpfung mit der besten verfügbaren externen klinischen Evidenz aus systematischer Forschung zu bringen ist [14].

Anderenfalls droht medizinischer Ausbildung und Praxis eine Vereinseitigung mit Schwerpunktbildung auf Algorithmen, während die in der klinischen Praxis notwendige Kontextualisierung und Individualisierung zu kurz kommen [15]. Dies ist von immenser Bedeutung, da Deindividualisierung und Beeinträchtigungen beim Eintreten sowie Fürsprache für Patienten („impaired patient agency“) als Bestandteile einer in der medizinischen Praxis beobachtbaren Dehumanisierung beschrieben werden [16].

Integrative Medizin und Auseinandersetzung mit der Perspektivenpluralität vorhandener Ansätze in Wissenschaft und Gesundheitsversorgung

Gerade im Hinblick auf Patientenpräferenzen und die adäquate Auswahl diagnostischer und therapeutischer Schritte, aber auch zur Stärkung des „routinierten Umgang[s] mit wissenschaftlichen Methoden und Konzepten“ (Masterplan Medizinstudium 2020, Abs. 4) kann die Einbindung der Integrativen Medizin in die Primärausbildung zur Ärztin/zum Arzt entscheidende Beiträge leisten. Integrative Medizin wird verstanden als „die Praxis der Medizin, welche die Bedeutung der Beziehung zwischen Arzt und Patienten betont. Sie konzentriert sich auf die ganze Person und ist durch Evidenz informiert. Sie nutzt alle angemessenen therapeutischen und lebensstilbezogenen Möglichkeiten und angemessene Kollaboration aller Gesundheitsberufe und -disziplinen, um optimale Gesundheit und Heilung zu erreichen.“ [https://imconsortium.org/about/introduction/]. Zudem nutzt

Integrative Medizin die reiche Vielfalt therapeutischer Systeme mit konventionellen und komplementären Herangehensweisen [17], [18]. Diese Auseinandersetzung ist einerseits eine wichtige und prinzipielle Vorbereitung für den akademischen Diskurs, indem methodisch die Frage nach dem wechselseitigen Ergänzungspotential sowie dem gegenseitigen Ausschluss der unterschiedlichen medizinischen Ansätze zu verfolgen ist [19], [20]. Andererseits bereitet sie auf die alltägliche Versorgungsrealität bei hoher nationaler und internationaler Inanspruchnahme von Komplementärmedizin durch Patienten bzw. Anwendung in der ambulanten Versorgung vor [21-23]. Weiterhin fördert sie den respektvollen Dialog innerhalb der Ärzteschaft im Sinne des Medizinpluralismus (vgl. [24]).

Studierende fordern eine Auseinandersetzung mit komplementärmedizinischen Verfahren; die theoretische Vermittlung solcher Lehrinhalte, kombiniert mit praktischen Erfahrungen „am eigenen Leib" kann zur kritischen Reflexion und zur Entwicklung einer ganzheitlichen, patientenorientierten Haltung unter Medizinstudierenden beitragen [25]. Ein weiteres, erfahrungsbasiertes Unterrichtsmodell mit der Zielsetzung der metakognitiven Auseinandersetzung mit verschiedenen medizinischen Systemen, deren Grundannahmen und Herangehensweisen wurde seitens des Ausschusses entwickelt [26]. Die Förderung einer eigenen Urteilsbildung ist gerade vor dem Hintergrund der o.g. hohen Versorgungsrelevanz, Berücksichtigung der Patientenpräferenz, Beratungsbedarfs und Diskussion zur EBM von Relevanz.

Ärztliche Selbstfürsorge und -regulation als Bestandteil professioneller Persönlichkeitsentwicklung

Um den sich im 21. Jahrhundert wandelnden An- und Herausforderungen des ärztlichen Berufs und damit der medizinischen Ausbildung [27] begegnen zu können, scheint eine frühe Förderung von Selbstfürsorge und -regulation bei Studierenden indiziert. Dies entspricht auch dem neu in das ärztliche Gelöbnis resp. der Genfer Deklaration des Weltärztebundes aufgenommen Passus: „Ich werde auf meine eigene Gesundheit, mein Wohlergehen und meine Fähigkeiten achten, um eine Behandlung auf höchstem Niveau leisten zu können" [28].

Zur Konkretisierung und Umsetzung der obigen Gesichtspunkte werden folgende Maßnahmen vorgeschlagen:

- Stärkere Orientierung an mehrdimensionalen anthropologischen Modellen und Thematisierung der verschiedenen, auch sozio-kulturell-historisch bedingten, Konzepte von und Perspektiven auf Gesundheit, Krankheit und Heilung.
- Schaffung strukturierter Lernumgebungen zum Erwerb von Kompetenzen resp. vertrauenswürdigen professionellen Aktivitäten (EPAs) hinsichtlich/unter Nutzung
    a. patientenzentrierter Beziehungsgestaltung und Kommunikation unter Berücksichtigung aller (einschließlich der komplementären) Diagnose- und Behandlungsoptionen und sowie Möglichkeiten der Selbstfürsorge (self-care) sowie Lebensstil-Veränderungen.
    b. umfassender Ausbildung der Anamnese- und Untersuchungstechniken auf den Grundlagen der integrativen Versorgungsansätze.
    c. früher und longitudinaler Einbindung von Real- und Simulationspatienten in die praktische, ambulante und klinisch-praktische Lehre unter Supervision, Feedback und Reflexion ihrer psychosozialen und individuellen Bedürfnisse, beginnend im ersten Studienjahr und über den gesamten Studienverlauf.

d. früher und longitudinaler Einbindung interprofessioneller Settings in die Patientenbegegnungen, Anamnese und Therapieplanung unter Orientierung am Therapieziel des Patienten und dessen Ressourcen.

- Vermittlung von Fähigkeiten zur strukturierten Förderung der Patienten-Selbstfürsorge (self-care) und Salutogenese (ressourcenintegrierend), sowie der Selbstfürsorge und -regulation der Vermittlung von Fähigkeiten zur strukturierten Förderung der Selbstfürsorge und -regulation der Studierenden
- Verbindliche Vermittlung von Basiswissen und Förderung eigenständiger, kritischer Urteilsbildung zur Integrativen Medizin, unter Berücksichtigung und Auseinandersetzung mit verschiedenen medizinischen Systemen wie Traditioneller Chinesischer Medizin, Homöopathischer Medizin, Ayurvedischer Medizin, Anthroposophischer Medizin, ausgewählten komplementärmedizinischen Verfahren sowie klassischen Naturheilverfahren, incl. stärkerer Thematisierung der Auswirkungen von Placebo- und Noceboeffekten im Bereich der konventionellen und integrativen/komplementären medizinischen Versorgung.
- Curricular angebotene Praktika in integrativmedizinischen Einrichtungen (Kliniken, Instituten, Lehrpraxen, etc.), um die Ausübung von Perspektivenpluralismus im Praxisalltag erleben und erfahren zu können.

Beigetragen von (alphab.): Angelika Homberg, Stefanie Joos, Stefanie Merse, Beate Stock-Schröer, Christian Scheffer, Diethard Tauschel, Jan Valentini

Literaturverzeichnis

1. Heusser P, Scheffer C, Neumann M, Tauschel D, Edelhäuser F. Towards non-reductionistic medical anthropology, medical education and practitioner–patient-interaction: The example of Anthroposophic Medicine. Patient Educ Couns. 2012;89(3):455-460. doi: 10.1016/j.pec.2012.01.004
2. Huber M, Knottnerus JA, Green L, van der Horst H, Jadad AR, Kromhout D, Leonard B, Lorig K, Loureiro MI, van der Meer JW, Schnabel P, Smith R, van Weel C, Smid H. How should we define health? BMJ. 2011;343:d4163. doi: 10.1136/bmj.d4163
3. Witt CM, Chiaramonte D, Berman S, Chesney MA, Kaplan GA, Stange KC, Woolf SH, Berman BM. Defining health in a comprehensive context: A new definition of integrative health. Am J Prev Med. 2017;53(1):134-137. doi: 10.1016/j.amepre.2016.11.029
4. Leventhal H, Leventhal EA, Cameron L. Representations, procedures, and affect in illness self-regulation: A perceptual-cognitive model. In: Baum A, Revenson TA, Singer JE, eds. Handbook of health psychology. Mahwah: Lawrence Erlbaum; 2001. p.19-47
5. Matthiessen PF. Einzelfallforschung zwischen Evidence based Medicine und Narrative based Medicine. 11 Internationaler Coethener Erfahrungsaustausch (ICE 11). Köthen (Anhalt): WissHom; 2011.
6. Tauschel D, Scheffer C, Balzereit S, Hofmann M, Edelhäuser F. How can clinical thinking be taught and trained practically? The concept of Real Patient Study Days. AMEE Conference; Trondheim: Association for Medical Education in Europe (AMEE); 2007. p.116
7. Steven K, Wenger E, Boshuizen H, Scherpbier A, Dornan T. How clerkship students learn from real patients in practice settings. Acad Med. 2014;89(3):469-476. doi: 10.1097/ACM.0000000000000129
8. Sandars J. The use of reflection in medical education: AMEE Guide No. 44. Med Teach. 2009;31(8):685-695.

9. Scheffer C, Tauschel D, Cysarz D, Hahn E, Längler A, Riechmann M, Edelhäuser F. Lernen durch aktive Partizipation in der klinischen Patientenversorgung-Machbarkeitsstudie einer internistischen PJ-Ausbildungsstation. GMS Z Med Ausbild. 2009;26(3):Doc31. doi:10.3205/zma000623
10. Lutz G, Scheffer C, Edelhaeuser F, Tauschel D, Neumann M. A reflective practice intervention for professional development, reduced stress and improved patient care—A qualitative developmental evaluation. Patient Educ Couns. 2013;92(3):337-345. doi: 10.1016/j.pec.2013.03.020
11. Scheffer C, Valk-Draad MP, Tauschel D, Büssing A, Humbroich K, Längler A, Zuzak T, Köster W, Edelhäuser F, Lutz G. Students with an autonomous role in hospital care–patients perceptions. Med Teach. 2018;40(9):944-952. doi: 10.1080/0142159X.2017.1418504
.12. Peters H, Holzhausen Y, Boscardin C, ten Cate O, Chen HC. Twelve tips for the implementation of EPAs for assessment and entrustment decisions. Med Teach. 2017;39(8):802-807. doi: 10.1080/0142159X.2017.1331031
13. ten Cate O, Chen HC, Hoff RG, Peters H, Bok H, van der Schaaf M. Curriculum development for the workplace using entrustable professional activities (EPAs): AMEE guide no. 99. Med Teach. 2015;37(11):983-1002. doi: 10.3109/0142159X.2015.1060308
14. Sackett DL, Rosenberg WM, Gray JM, Haynes RB, Richardson WS. Evidence based medicine: what it is and what it isn't. 1996. Clin Orthop Relat Res. 2007;455:3-5.
15. Greenhalgh T, Howick J, Maskrey N. Evidence based medicine: a movement in crisis? BMJ. 2014;348:g3725. doi: 10.1136/bmj.g3725
16. Haque OS, Waytz A. Dehumanization in medicine: Causes, solutions, and functions. Perspect Psychol Sci. 2012;7(2):176-186. doi: 10.1177/1745691611429706
17. Briggs JP. 14e: Complementary, Alternative, and Integrative Health Practices. In: Kasper D, Fauci A, Hauser S, Longo D, Jameson JL, Loscalzo J, eds. Harrison's Principles of Internal Medicine; 2017.
18. Kligler B, Maizes V, Schachter S, Park CM, Gaudet T, Benn R, Lee R, Remen RN; Education Working Group, Consortium of Academic Health Centers for Integrative Medicine. Core competencies in integrative medicine for medical school curricula: a proposal. Acad Med. 2004;79(6):521-531.
19. Matthiessen PF. Paradigmenpluralität und Individualmedizin. In: Matthiessen PF, ed. enorientierung und Professionalität Festschrift 10 Jahre Dialogforum Pluralismus in der Medizin. 2. erweiterte Auflage ed. Bad Homburg: Verlag Akademische Schriften (VAS); 2011.
20. Matthiessen PF. 10 Jahre Dialogforum Pluralismus in der Medizin. Warum es uns gibt, wer wir sind und was wir wollen. In: Matthiessen PF, ed. enorientierung und Professionalität Festschrift 10 Jahre Dialogforum Pluralismus in der Medizin. 2. erweiterte Auflage ed. Bad Homburg: Verlag Akademische Schriften (VAS); 2011.
21. Horneber M, Bueschel G, Dennert G, Less D, Ritter E, Zwahlen M. How many cancer patients use complementary and alternative medicine: a systematic review and metaanalysis. Integr Cancer Ther. 2012;11(3):187-203. doi: 10.1177/1534735411423920
22. Linde K, Alscher A, Friedrichs C, Joos S, Schneider A. Die Verwendung von Naturheilverfahren, komplementären und alternativen Therapien in Deutschland-eine systematische Übersicht bundesweiter Erhebungen. Compl Med Res. 2014;21(2):111-8.
23. Barnes PM, Bloom B, Nahin RL. Complementary and alternative medicine use among adults and children; United States, 2007. Nat Health Stat Report. 2008;12(December 10).

24. Kiene H, Heimpel H. Ärztliche Professionalität und Komplementärmedizin: Was ist seriöse ärztliche Therapie? Dtsch Arztebl. 2010;107(12):477.
25. Valentini J, Glassen K, Eicher C, Washington-Dorando P, Weinschenk S, Musselmann B, Steinhäuser J, Joos S. „Kritische Diskussion sollte mehr gefördert werden!“–Eine qualitative Auswertung der Lehrevaluation von Medizinstudierenden zur komplementärmedizinischen Lehre. Dtsch Med Wochenschr. 2018;143(14):e125-e130. doi: 0.1055/a-0575-6851
26. Tauschel D, Edelhäuser F, Scheffer C. How can approaches of different medical systems be made visible, brought into dialogue, and reflected? - Real Patient Study Days Integrative Medicine. AMEE Conference; Basel: Association for Medical Education in Europe (AMEE); 2018. p.929
27. Frenk J, Chen L, Bhutta Z, Cohen J, Crisp N, Evens T, Fineberg H, Garcia P, Ke Y, Kelly P, Kistnasamy B, Meleis A, Naylor D, Pablos-Mendez A, Reddy S, Scrimshaw S, Sepulveda J, Serwadda D, Zurayk H. Health professionals for a new century: transforming education to strengthen health systems in an interdependent world. Lancet. 2011;376(9756):1923-1958. doi: 10.1016/S0140-6736(10)61854-5
28. World Medical Association. WMA Declaration of Geneva. The Physician’s Pledge 2017 Geneva: World Medical Association; 2018. Zugänglich unter/available from: https://www.wma.net/policies-post/wma-declaration-of-geneva/
