# Supplementary material for: Position of the advisory and executive board of the German Association for Medical Education (GMA) regarding the “masterplan for medical studies 2020”
Source: GMS J Med Educ. 2019 Aug 15;36(4):Doc46. doi: 10.3205/zma001254 (PMC6737258; doi:10.3205/zma001254)
Supplement: Committee on Gender, Diversity and Career Development in Medical Education [german] [file JME-36-4-46-s-009.pdf]

Stellungnahme des Ausschusses Gender, Diversity und Karriereentwicklung in der medizinischen Aus- und Weiterbildung

Der Ausschuss begrüßt den vorliegenden Masterplan Medizinstudium 2020 mit dem Ziel einer kompetenzorientierten, wissenschaftlichen und praxisnahen Ausbildung, der Stärkung der Allgemeinmedizin, der konsequenten Orientierung an den Patientinnen und Patienten und deren Bedürfnisse sowie der Integration kommunikativer Kompetenzen.

Die bestehende und zunehmende Diversität in unserer Bevölkerung und somit auch in der Patientenschaft und unter Studierenden machen es zunehmend notwendig, dass Diversitäts- und Genderaspekte ausreichend in der Aus- und Weiterbildung in der Medizin und in den Gesundheitsprofessionen berücksichtigt werden. Nach dem Allgemeinen Gleichbehandlungsgesetz (AGG) umfasst Diversität sechs Kategorien: Alter, Geschlecht, Ethnie, körperliche Beeinträchtigung, sexuelle Orientierung und Religion [https://dejure.org/gesetze/AGG]. Darüber hinaus spielen aber auch viele andere Aspekte eine Rolle, wenn es um Inklusion oder Diskriminierung geht (z.B. soziale Zugehörigkeit, Bildungshistorie, individuelle körperliche, funktionelle und geistige Merkmalsausprägungen bis hin zu Haarfarbe und Körperform, Leistungsfähigkeit, Aussprache, um einige wenige zu nennen).

Der Ausschuss betrachtet es daher als notwendig, dass Diversitäts-, insbesondere Genderkompetenzen, umfassender als bislang in das Curriculum mitaufgenommen werden, insbesondere da im medizinischen Bereich der Umgang mit Diversität explizit Teil der beruflichen Aufgaben darstellt. Wir verweisen hier auf das Positionspapier des Ständigen Ausschusses der Europäischen Ärztinnen und Ärzte (CPME: Comité Permanent des Médecins Européens) von 2016, das die Bedeutung der curricularen Integration der Kategorie Geschlecht betont [1]. Neben der inhaltlichen Integration in die Curricula, sollten Diversitätsaspekte auch in der Hochschuldidaktik Berücksichtigung finden [2].

Im Jahr 2013 hat der Ausschuss 207 diversitäts- und genderbezogene Lernziele für den Nationalen Kompetenzbasierten Lernzielkatalog Medizin (NKLM) vorgeschlagen, davon wurden 82 übernommen, das entspricht 4% der Gesamtzahl der Lernziele im NKLM [3]. Dem Ausschuss ist es wichtig, dass diese Lernziele langfristig nicht nur im NKLM verankert bleiben, sondern auch Eingang in die die Gegenstandskataloge bzw. die Ärztliche Prüfung finden. Insbesondere auch im Sinne der Patienten/innenorientierung und der individualisierten Medizin sind gendermedizinische und gendersensible Lehrinhalte unerlässlich. Mittelfristig ist anzustreben, auch andere Diversitäts-relevante Lernziele zu diskutieren und nachhaltig in die Medizinische Ausbildung einzubetten.

Im Bereich der wissenschaftlichen Kompetenzen sieht der Ausschuss ebenfalls dringenden Bedarf, Diversitäts- und Genderaspekte zu integrieren, so dass bereits in der Planung von (medizinischen) Studien und in den Studienkohorten beide Geschlechter vertreten sind und Studienergebnisse geschlechterspezifisch und diversitätssensibel ausgewertet werden. Wir verweisen hier auf die Veröffentlichung der National Institutes of Health, das die Integration von geschlechtsspezifischen Aspekten in die Forschung fordert [4].

Den Studierenden sollten zudem früh Informationen über Möglichkeiten der Karriereentwicklung für Ärztinnen und Ärzten geboten werden. Die Vereinbarkeit von Studium und Familie sollte gegeben sein. Aufgrund der steigenden Zahl von Studentinnen und der zukünftig höheren Anzahl von Ärztinnen sollten Teilzeitarbeits- sowie Teilzeit-

Studienmodelle ermöglicht werden. Es sollte dabei aber auch erleichtert werden, trotz Familiengründung Vollzeit zu arbeiten/zu studieren bzw. Karrierenachteile durch Teilzeittätigkeit zu verhindern/abzubauen.

Diskriminierungsfreie Prüfungen, die der Diversität der Studierenden gerecht werden, hält der Ausschuss für obligat und essenziell. Weder bei den Prüfungen noch bei der Zulassung zum Studium dürfen Diskriminierungen nach Alter, Geschlecht, ethnischer Herkunft, körperlicher Beeinträchtigung, Religion, sexueller Orientierung oder anderer Diversitätskategorien erfolgen.

Insgesamt plädieren wir für die Integration von Diversitäts-, insbesondere Genderaspekten, in medizinische Curricula, in die Hochschuldidaktik, in Studiengangsstrukturen, in die Regularien zur Zulassung zum Studium sowie für deren Berücksichtigung bei der Planung, Durchführung und Bewertung von Prüfungen.

Beigetragen von (alphab.): Irene Brunk, Sabine Ludwig

Literaturverzeichnis

1. Comité Permanent Des Médecins Européens (CPME). CPME Policy on Sex and Gender in medicine. Brüssel: Comité Permanent Des Médecins Européens (CPME); 2016.
2. Ebenfeld M. Checkliste zur gender- und diversitätsbewussten Didaktik Berlin: Freie Universität Berlin; 2017. Zugänglich unter/available from: https://www.htw-berlin.de/fileadmin/HTW/Zentral/ZR_VI_-_Frauenfoerderung_und_Gleichstellung/GenderindieLehre/Checkliste_gender-diversitaet-Didaktik_Ebenfeld.pdf.
3. Ludwig S, Romero YR, Balz J, Petzold M. The use of quality assurance instruments and methods to integrate diversity aspects into health professions study programmes. MedEdPublish. 2018;7.
4. Clayton JA, Collins FS. Policy: NIH to balance sex in cell and animal studies. Nature News. 2014;509(7500):282-283.
