# Supplementary material for: Position of the advisory and executive board of the German Association for Medical Education (GMA) regarding the “masterplan for medical studies 2020”
Source: GMS J Med Educ. 2019 Aug 15;36(4):Doc46. doi: 10.3205/zma001254 (PMC6737258; doi:10.3205/zma001254)
Supplement: Committee for Cultural Competence and Global Health [german] [file JME-36-4-46-s-010.pdf]

Stellungnahme des Ausschusses Kulturelle Kompetenz und Global Health

Der Ausschuss begrüßt die Zielrichtung des Masterplan Medizinstudium 2020, die Lehre künftig stärker „an der Vermittlung arztbezogener Kompetenzen" auszurichten und „die konsequente Orientierung am Patienten und seinen Bedürfnissen" in den Mittelpunkt zu stellen. Auch die Betonung der Arzt-Patienten-Kommunikation sowie der sozialen und kommunikativen Kompetenzen der Studierenden unterstützen wir ausdrücklich. Um dieser Zielsetzung aber tatsächlich gerecht werden zu können, ist es unserer Ansicht nach notwendig, auch die soziokulturellen und internationalen Aspekte von Gesundheit, Medizin und ärztlichem Handeln systematisch zu berücksichtigen [1].

Im Hinblick auf die generelle Ausrichtung des Masterplan Medizinstudium 2020 stellen wir fest, dass neben dem demographischen Wandel und der Unterversorgung strukturschwacher Regionen – den zentralen Themen des Masterplans – auch Migration und eine wachsende sozio-kulturelle Diversität der Bevölkerung zu den zentralen Herausforderungen unserer Gesellschaft gehören und in Zukunft nicht an Bedeutung verlieren werden. Wir verweisen hier auf die jüngsten Empfehlungen der Hochschulrektorenkonferenz zur Internationalisierung der Curricula von 2017 [2].

Konkret ist es notwendig, im Hinblick auf die Kompetenzorientierung des Medizinstudiums, die Förderung kultureller Kompetenz ausdrücklich in das Regelstudium zu integrieren. Der NKLM bietet dazu bereits jetzt eine wichtige Orientierung. Im Hinblick auf die Arzt-Patienten-Kommunikation ist ferner eine systematische Integration von Lerninhalten zu Kommunikation über sprachliche Grenzen hinweg nötig, einschließlich der fachgerechten Nutzung technischer Hilfsmittel und der Zusammenarbeit mit Sprach- und Kulturmittlern. Auch zu diesem Thema bietet der NKLM geeignete Lernziele. Für Erhalt, Förderung und Ausbau sozialer und kommunikativer Kompetenzen von Studierenden gibt es schließlich einen Bedarf an bisher im Masterplan nicht angesprochenen Lehrangeboten, die die Reflexionsfähigkeit und das systematische Nachdenken über soziale, ethische und moralische Fragen fördern. Es ist vielfach dokumentiert, dass Lehrinhalte aus dem Themenfeld Kulturelle Kompetenz und Global Health (z.B. anknüpfend an studienbezogene Auslandsaufenthalte von Studierenden mit systematischer Vor- und Nachbereitung) hier ein erhebliches Potential besitzen [1], [2]. Insgesamt plädieren wir für eine systematische Integration internationaler und interkultureller Inhalte als Voraussetzung für eine zeitgemäße medizinische Ausbildung. Oder, wie es die Bundesvertretung der Medizinstudierenden in Deutschland (bvmd) bereits vor knapp 10 Jahren forderte: „Lehre am Puls der Zeit" [3].

Beigetragen von (alphab.): Michael Knipper, Claudia Mews

Literaturverzeichnis

1. Mews C, Schuster S, Vajda C, Lindtner-Rudolph H, Schmidt LE, Bösner S, Güzelsoy L, Kressing F, Hallal H, Peters T, Gestmann M, Hempel L, Grützmann T, Sievers E, Knipper M. Cultural Competence and Global Health: Perspectives for Medical Education–Position paper of the GMA Committee on Cultural Competence and Global Health. GMS J Med Educ. 2018;35(3):Doc28. doi: 10.3205/zma001174

2. Hochschulrektorenkonferenz. Zur Internationalisierung der Curricula. Empfehlung der HRK-Mitgliederversammlung vom 9.5.2017 [press release]. Bonn: Hochschulrektorenkonferenz; 2017.
3. Bozorgmehr K, Last K, Müller A, Schubert K. Lehre am Puls der Zeit-Global Health in der Medizinischen Ausbildung: Positionen, Lernziele und methodische Empfehlungen. GMS Z Med Ausbild. 2009;26(2):Doc20. doi: 10.3205/zma000612
