# Supplementary material for: Position of the advisory and executive board of the German Association for Medical Education (GMA) regarding the “masterplan for medical studies 2020”
Source: GMS J Med Educ. 2019 Aug 15;36(4):Doc46. doi: 10.3205/zma001254 (PMC6737258; doi:10.3205/zma001254)
Supplement: Faculty and Organizational Development Committee in Education [german] [file JME-36-4-46-s-011.pdf]

Stellungnahme des Ausschusses Personal- und Organisationsentwicklung in der Lehre

Der Ausschuss für Personal- und Organisationsentwicklung in der Lehre der GMA begrüßt viele der im Masterplan Medizinstudium 2020 beschriebenen Vorschläge zur Veränderung der Studienstruktur und der Ausbildungsinhalte im Medizinstudium ausdrücklich. Aus der Perspektive derjenigen, die durch die Qualifizierung der Lehrenden an den Medizinischen Fakultäten zur Qualitätsentwicklung und -sicherung der Lehre beitragen, stellen sich jedoch auch einige Fragen.

Eine der zentralen Maßnahmen des Masterplan Medizinstudium 2020 ist die Umsetzung einer kompetenzorientierten ärztlichen Ausbildung auf Grundlage des NKLM. Damit sind tiefgreifende strukturelle und curriculare Veränderungen an den Fakultäten verbunden. So machen etwa die stärkere curriculare Integration der ambulanten Medizin und der Ausbau der interprofessionellen Ausbildung neue Kooperationen und Konzepte – auch für die Personal- und Organisationsentwicklung in der Lehre erforderlich. Außerdem müssen Lehr- und Prüfungsformate neu entwickelt bzw. angepasst werden, um den verschiedenen im NKLM definierten Kompetenzbereichen gerecht zu werden.

Um diese Veränderungen bewältigen zu können, sind umfassende Angebote zur Personal- und Organisationsentwicklung, zusätzlich zu den bereits etablierten Maßnahmen an den Fakultäten notwendig. Diese Maßnahmen müssen auf die jeweiligen lokalen Besonderheiten der Fakultäten zugeschnitten werden, da die standortspezifischen Rahmenbedingungen großen Einfluss auf die Qualität von Lehre haben [1]. Im Rahmen des Medizindidaktik-Netzwerks (MDN), einer Arbeitsgruppe des Medizinischen Fakultätentages, an der alle Medizinischen Fakultäten in Deutschland beteiligt sind, wurden in den letzten Jahren Qualitätsstandards für Qualifizierungsmaßnahmen entwickelt und erfolgreich implementiert [2]. Die meisten Fakultäten verfügen bereits über eigene Angebote der medizindidaktischen Qualifizierung von Lehrenden, viele sind untereinander auf Landes- oder Bundeseben vernetzt und ermöglichen den Lehrenden die Teilnahme an Qualifizierungsmaßnahmen anderer Standorte. Diese bereits etablierten, akzeptierten und gut funktionierenden Strukturen sollten in erster Linie für den durch den Masterplan Medizinstudium 2020 notwendig werdenden Qualifizierungsbedarf genutzt werden.

Das gilt, neben den Forderungen nach der Integration für ambulante Medizin, interprofessionelle Lehre und mehr Wissenschaftlichkeit im Studium auch für die im Masterplan Medizinstudium 2020 unter Punkt 27 beschriebenen Schulungen für die OSCE-Prüfungen. Viele Fakultäten verfügen bereits über langjährige Erfahrungen mit diesem Prüfungsformat und haben entsprechende Qualifizierungsmaßnahmen für ihre Lehrenden entwickelt [3], [4]. Vor diesem Hintergrund erscheint es naheliegender, diese bereits bestehende Expertise als Grundlage für weitere Entwicklungen zu nutzen als in erster Linie auf zentrale Schulungen durch das IMPP zu setzen. Insgesamt erscheint es auch aus praktischen Erwägungen (zeitlicher Aufwand, Reisekosten, etc.) und vor dem Hintergrund der bereits etablierten Netzwerke und Strukturen sinnvoller, Multiplikatorinnen und Multiplikatoren zu schulen, die dann vor Ort entsprechende Qualifizierungsmaßnahmen anbieten und durchführen können, als die Lehrenden in der Breite zentral auszubilden. Die Standards und Qualitätskriterien für die entsprechenden Qualifizierungsangebote sowie die Konzepte für die Umsetzung könnten dabei von den Fakultäten selbst – innerhalb der vorhandenen Strukturen – entwickelt werden. Zudem ist sicherzustellen, dass

Lehrverantwortliche (z.B. in den Dekanaten, aber auch an einzelnen Institutionen und Kliniken) sowie Lehrende adäquat und rechtzeitig auf Neuerungen (z.B. Unterrichtsformate, Evaluationskonzepte und Prüfungen) vorbereitet werden. Dies muss professionell und unter Gewährleistung bundesweit gültiger Qualitätskriterien erfolgen.

Für viele der im Masterplan Medizinstudium 2020 beschriebenen Vorschläge sind somit neue und vielfältige Angebote der Personal- und Organisationsentwicklung erforderlich. Diese können aufgrund der dargestellten langjährigen Erfahrungen und angesichts der starken Vernetzung untereinander am besten von den Fakultäten selbst konzipiert und durchgeführt werden. Erforderlich sind dazu allerdings umfangreiche personelle und finanzielle Ressourcen, die den Fakultäten zusätzlich zur Verfügung gestellt werden müssen.

Literaturverzeichnis

1. Giesler M, Karsten G, Ochsendorf F, Breckwoldt J. Rahmenbedingungen für exzellente Lehre in der Medizin; Das Frankfurter Modell der Rahmenbedingungen zur Sicherung der Lehr-und Lernqualität. GMS J Med Educ. 2017;34(4):Doc46. doi: 10.3205/zma001123
2. Lammerding-Koeppel M, Ebert T, Goerlitz A, Karsten G, Nounla C, Schmidt S, Stosch C, Dieter P. German MedicalTeachingNetwork (MDN) implementing national standards for teacher training. Med Teach. 2016;38(4):378-384. doi: 10.3109/0142159X.2015.1047752
3. Müller S, Dahmen U, Settmacher U. Objective Structured Clinical Examination (OSCE) an Medizinischen Fakultäten in Deutschland–eine Bestandsaufnahme. Gesundheitswesen. 2018;80(12):1099-1103. doi: 10.1055/s-0042-116435
4. Graf J, Smolka R, Holderried F, Wosnik A, Lammerding-Köppel M, Mohr D, Vlad E, Nikendei C, Zipfel S, Herrmann-Werner A. Ten Years of Objective Structured Clinical Examination at the Medical Faculty of Tübingen, Germany: Item Analysis and Students' Satisfaction. J Health Sci Educ. 2018;2(2):1-7. doi: 10.0000/JHSE.1000133
