# Supplementary material for: Position of the advisory and executive board of the German Association for Medical Education (GMA) regarding the “masterplan for medical studies 2020”
Source: GMS J Med Educ. 2019 Aug 15;36(4):Doc46. doi: 10.3205/zma001254 (PMC6737258; doi:10.3205/zma001254)
Supplement: Digitization – Technology-Assisted Learning and Teaching Committee [german] [file JME-36-4-46-s-012.pdf]

Stellungnahme des Ausschusses Digitalisierung – Technologie-unterstütztes Lernen und Lehren

Der von der Bund-Länder AG am 31.3.2017 beschlossene Masterplan Medizinstudium 2020 enthält eine Vielzahl möglicher Maßnahmen und zukünftiger Schwerpunkte zur Reform des Medizinstudiums. Obwohl der aktuelle Koalitionsvertrag und das Hochschulforum Digitalisierung dem Stellenwert der Digitalisierung höchste Bedeutung beimessen, wird diese erstaunlicherweise mit keinem Wort im Masterplan Medizinstudium 2020 erwähnt. Dies ist allein dadurch schon erstaunlich, da die Digitalisierung einen weitreichenden Wandel in nahezu allen Bereichen der Gesellschaft hervorgerufen hat [1], [2]. Auch die Strukturen der Lehr- und Lernorganisation sowie die Rollen und Anforderungsprofile von Studierenden und Lehrenden haben sich verändert. Dabei entstehen neue didaktische Möglichkeiten hinsichtlich der Kompetenz- und Wissensvermittlung, aber auch Herausforderungen, auf die die medizinischen Hochschulen entsprechend reagieren müssen [3], [4]. Ein Paradigmenwechsel hat die Rolle der Lehrpersonen von Wissensvermittelnden hin zu „Wissensbereitstellenden" verändert. Die veränderten Rollen der Lehrpersonen, die nicht mehr alleinige Bereitstellen von Wissen und Informationen sind, sondern vielmehr Lehrinhalte kollektiv suchen, entwickeln und diskutieren, erfordert andere Kompetenzen [5] und können durch die Nutzung digitaler Lehrformate unterstützt werden [6].

Es wird durch den GMA-Ausschuss Digitalisierung prognostiziert, dass die Digitalisierung im Gesundheitswesen bereits für heutige Medizinstudierende - und noch viel mehr für zukünftige Studierendengenerationen - wichtige Themen- und Arbeitsbereiche bestimmen wird. Derzeit gibt es zwei große Herausforderungen, auf die das Medizinstudium adäquat reagieren muss, die Digitale Lehr- und Lerntechnologien sinnvoll und flächendeckend einsetzten sowie digitale Kompetenzen vermitteln [7].

Digitale Lehre

In der medizinischen Ausbildung halten zunehmend neue digitale Szenarien, Konzepte und Methoden Einzug in die Lehre [8]. Im Gesundheitswesen wird der Ausbildung und dem Erwerb sowohl professionsspezifischer als auch interprofessioneller Kompetenzen eine zunehmende Bedeutsamkeit zugesprochen, für die spezifische Lehrformate erprobt und etabliert werden müssen [9]. Die Wirksamkeit von Blended-Learning-Szenarien konnte bereits in einigen Studien nachgewiesen werden [10]. Es zeigt sich ebenfalls, dass sich neue und innovative technologie-unterstützte Lern- und Lehrmethoden wie die Inverted-Classroom-Methode langsam etablieren [11]. Nicht zuletzt bieten die heute an vielen Stellen bereits eingesetzten informationstechnologischen Werkzeuge und Methoden (Lehrvideos, Open Educational Resources, TED u.a. elektronisches (self)Assessment, Simulationen, Virtuelle Patienten) einen so hohen didaktischen Mehrwert, dass Universitäten schwerlich darauf verzichten können [12], [13]. Die große Anzahl erfolgreicher (v.a. kommerzieller) E-Learning-Angebote ist ein deutlicher Indikator für die Sinnhaftigkeit des Einsatzes digitaler Medien im Rahmen der medizinischen Aus- sowie Weiterbildung und relevant für eine praxisnahe Ausbildung.

Des Weiteren erfordert die moderne Lehre in der hochschulmedizinischen Ausbildung anpassungsfähige informationstechnologische Infrastrukturen, wie Lernplattformen, um innovative Lehrkonzepte nicht von vornherein zu erschweren. Förderungsmaßnahmen, wie durch die Digitalisierungsstrategie des Bundes in Aussicht gestellt, sind bei gegebenen knappen Mittelzuweisungen an die medizinischen Fakultäten absolut notwendig.

Digitale Kompetenzen

Ein weiteres Beispiel sind die Stichworte „BigData“ für die Forschung oder „Telemedizin“ für die zukünftige Arbeitswelt von Ärztinnen und Ärzten. Die Entwicklung neuer informationstechnologischer Werkzeuge, wie elektronischen Patientenakten, Krankenhaus- und Röntgeninformationssystemen oder elektronischen Gesundheitskarten sind heute im Berufsalltag der Ärztinnen und Ärzten sehr bedeutsam. Schon während des Medizinstudiums sollte deshalb der Umgang mit diesen Werkzeugen, aber auch die kritische Auseinandersetzung mit möglichen informationstechnologischen Weiterentwicklungen und deren Folgen für die Medizin und die Gesellschaft gefordert und gefördert werden. Die im Masterplan erwähnten arztbezogenen Kompetenzen müssen dringend auch die digitalen Kompetenzen mitberücksichtigen, welche als Anforderung der Kompetenzorientierung von Hochschulstudiengängen laut der Bologna-Reform beschrieben werden. Ausbildungsinhalte des Medizinstudiums sollten folgerichtig nicht nur wissenschaftliche Erkenntnisse und praktische Fertigkeiten, sondern auch die „digitale Kompetenz“ zur Nutzung aktueller und zukünftiger Informationstechnologien miteinschließen. Curriculare Konzepte zur Vermittlung digitaler Kompetenzen, wie das bereits existierende Curriculum „Medizin im digitalen Zeitalter“ der Universitätsmedizin Mainz [14] sollten breiter eingesetzt werden.

Telemedizin

Auf S. 4 des Masterplan Medizinstudium 2020 wird postuliert, „die medizinischen Versorgungsprozesse werden immer komplexer. In Zukunft wird daher eine arbeitsteilige Zusammenarbeit mit mitbehandelnden bzw. hinzuzuziehenden Ärztinnen und Ärzten anderer Fachrichtungen […] eine noch stärkere Rolle spielen als bisher.“ Die naheliegende Forderung einer Schulung und Stärkung der kommunikativen Kompetenzen soll an dieser Stelle um die Forderung der Aufnahme von Lernzielen zum Umgang mit relevanten informationstechnologischen Entwicklungen und Konzepten ergänzt werden. Solche Lernziele sind bereits im NKLM aufzufinden (Bsp.: 10.7.1, 11.2.3, und besonders 14c.6.3). Es ist davon auszugehen, dass es auch auf Seiten der Lehre noch keine fertigen Konzepte zur Vermittlung von beispielsweise der NKLM-Lernziele 10.7.1.2 „im klinischen Arbeitsplatzsystem (KAS) Untersuchungen anfordern, Befunde dokumentieren …“ oder 10.7.1.5 „Lösungen der Telemedizin patientenorientiert einsetzen und Rahmenbedingungen der Gesundheitstelematik erläutern“ gibt. Es erscheint durchaus angemessen, auch auf diese große didaktische Herausforderung schon im Masterplan Medizinstudium 2020 direkt einzugehen, zumal die für einen sinnvollen Praxisbezug schon im Studium erforderlichen Lerninhalte und Vermittlungsmethoden vermutlich noch an keiner Stelle suffizient existieren.

Abschließend ist es von hoher Bedeutung, dass aktuelle Barrieren der Digitalisierung, wie beispielsweise geringe Medienkompetenz der Lehrenden, das Fehlen einer einheitlichen Regelung zur Anrechnung von Lehrdeputat von digitaler Lehre [15] und die mangelnde Unterstützung der Institutionen abgebaut, bzw. reduziert werden.

Der Ausschuss Digitalisierung fordert daher die Entwicklung einer nationalen Digitalisierungsstrategie im Masterplan Medizinstudium 2020. Die Entwicklungen der Digitalisierung müssen in der zukünftigen medizinischen Ausbildung abgebildet werden. Berücksichtigt werden sollen dabei sowohl die Studierenden als auch die Mitglieder der Fakultäten und weiterer Entscheidungsträger. Nur mit einer einheitlichen Integration der Digitalisierung in die medizinische Ausbildung können Ärztinnen und Ärzte auf die heutigen und zukünftigen Herausforderungen der modernen Berufswelt vorbereitet werden.

Konkret fordern wir:

- Die Implementierung von Themen der Digitalisierung in die studentische Ausbildung
- Eine nationale Initiative "Medizinische Ausbildung im digitalen Zeitalter"
- Die Stärkung der hochschuldidaktischen Ausbildung, insbesondere der Fachgebiete Medienpädagogik und Mediendidaktik
- Die Implementierung der Inverted-Classroom-Methode
- Die Stärkung und Verbreitung freier Bildungsressourcen nach der Open Educational Resources-Idee
- Die Nutzung "virtueller" Patienten
- Den Einsatz web-/cloud-basierter Anwendungen (Kommunikations-Plattformen; Social Media; Artefakt-Gestaltung mittels Apps wie z.B. Lehr-Videos, -Comics, -Blogs, -Internetseiten, MindMaps, Concept Maps, Storytelling, Document / Media Sharing)
- Klärung und Vereinheitlichung der Anrechnung des Lehrdeputats für digitale Lehre.

Verfasst als kooperatives Projekt aller Ausschussmitglieder

Literaturverzeichnis

1. Bischof L, von Stuckrad T. Die digitale (R)evolution? Chancen und Risiken der Digitalisierung akademischer Lehre. Gütersloh: CHE gemeinnütziges Centrum für Hochschulentwicklung; 2013.
2. Hochschulforum Digitalisierung. The Digital Turn – Hochschulbildung im digitalen Zeitalter. Berlin: Hochschulforum Digitalisierung; 2016.
3. Handke J. Handbuch Hochschullehre Digital: Leitfaden für eine moderne und mediengerechte Lehre. Marburg: Tectum Wissenschaftsverlag; 2015.
4. Hochschulforum Digitalisierung. Diskussionspapier - 20 Thesen zur Digitalisierung der Hochschulbildung. Berlin: Hochschulforum Digitalisierung; 2015.
5. Lackner E, Kopp M. Lernen und Lehren im virtuellen Raum. Herausforderungen, Chancen, Möglichkeiten. In: Rummler K, ed. Lernräume gestalten - Bildungskontexte vielfältig denken. Medien in der Wissenschaft. Münster: Waxmann; 2014. p.174-186
6. Rummler K. Lernräume gestalten - Bildungskontexte vielfältig denken. Münster: Waxmann; 2014.
7. Haag M, Igel C, Fischer MR, German Medical Education Society (GMA), Committee "Digitization - Technology-Assited Learning and Teaching". Digital Teaching and Digital Medicine: A national initiative is needed. GMS J Med Educ. 2018;35(3):Doc43. doi: 10.3205/zma001189
8. Kuhn S, Frankenhauser S, Tolks D. Digitale Lehr-und Lernangebote in der medizinischen Ausbildung. Bundesgesundheitsblatt Gesundheitsforschung Gesundheitsschutz. 2018;61(2):201-209. doi: 10.1007/s00103-017-2673-z
9. Fabry G, Fischer MR. Medical Education in Germany–Work in Progress. GMS Z Med Ausbild. 2014;31(3):Doc36. doi: 10.3205/zma000928
10. Stegmann K, Fischer F. Auswirkungen digitaler Medien auf den Wissens-und Kompetenzerwerb an der Hochschule. München: Ludwig-Maximilian-Universität; 2016.
11. Tolks D, Bischoff T, Bauer D. Eine Einführung in die Inverted-Classroom-Methode in der medizinischen Ausbildung. In: Jahrestagung der Gesellschaft für Medizinische Ausbildung (GMA); 14.09.-17.09.2016; Bern. Düsseldorf: German Medical Science GMS Publishing House; 2016. DocWS-P04-323. doi: 10.3205/16gma007

12. Guo PJ, Kim J, Rubin R. How video production affects student engagement: an empirical study of MOOC videos. Proceedings of the first ACM conference on Learning@ scale conference; 2014.
13. Hege I, Kononowicz AA, Tolks D, Edelbring S, Kuehlmeyer K. A qualitative analysis of virtual patient descriptions in healthcare education based on a systematic literature review. BMC Med Educ. 2016;16(1):146. doi: 10.1186/s12909-016-0655-8
14. Kuhn S, Kadioglu D, Deutsch K, Michl S. Data Literacy in der Medizin. Onkologe. 2018;24(5):368-377.
15. Müller C, Füngerlings S, Tolks D; Arbeitsgruppe E-Learning des Komptenznetzes Medizinlehre Bayern. Teaching load–a barrier to digitalisation in higher education? A position paper on the framework surrounding higher education medical teaching in the digital age using Bavaria, Germany as an example. GMS J Med Educ. 2018;35(3):Doc34. doi: 10.3205/zma001180
