# Supplementary material for: Position of the advisory and executive board of the German Association for Medical Education (GMA) regarding the “masterplan for medical studies 2020”
Source: GMS J Med Educ. 2019 Aug 15;36(4):Doc46. doi: 10.3205/zma001254 (PMC6737258; doi:10.3205/zma001254)
Supplement: Assessment Committee [german] [file JME-36-4-46-s-013.pdf]

Stellungnahme des Ausschusses Prüfungen

Im Masterplan Medizinstudium 2020 steckt Potential zur Weiterentwicklung der Prüfungskultur in der medizinischen Ausbildung in Deutschland, auch der Ärztlichen Prüfung. Dieses ergäbe sich aus einer konsequenten Umsetzung des übergeordneten Leitgedankens der kompetenzorientierten Ausbildung geradezu notwendig.

Zwar verzichtet der Masterplan Medizinstudium 2020 auf eine Definition von Kompetenz, referenziert aber mehrfach den NKLM [1], der sich wiederum auf Weinerts Definition [2] bezieht, den *„bei einem Individuum verfügbaren oder durch sie erlernbaren kognitiven Fähigkeiten und Fertigkeiten, um bestimmte Probleme zu lösen, sowie die damit verbundenen motivationalen, volitionalen und sozialen Bereitschaften und Fähigkeiten, um die Problemlösungen in variablen Situationen erfolgreich und verantwortungsvoll nutzen zu können.*“ Was bedeutet dies nun für Prüfungen? Frank und Kollegen destillieren in einer systematischen Analyse von Definitionen kompetenzbasierter Ausbildung heraus, welche zentrale Schnittmenge sich für eine kompetenzorientierte Prüfungskultur ergibt: Demzufolge orientieren sich Prüfungen an vordefinierten Standards oder Meilensteinen, die den Fortschritt auf das definierte Ausbildungsziel abbilden. Prüfungsleistungen wären demzufolge kriteriumsorientiert zu bewerten, so dass der Lernerfolg der Lernenden nicht untereinander, sondern in Bezug auf ebendiese definierten Standards bewertet wird. Dies schließe auch nicht aus, dass Prüfungshürden eingebaut werden, die es zu überwinden gelte, bevor man im Curriculum weiter voranschreiten dürfe [3].

Der erste Punkt verweist direkt auf das didaktische Konzept des *Constructive Alignment* [4], also die durch Lernziele koordinierte Abstimmung von Unterricht, Lernen und Prüfungen zugunsten der Sinnbildung im Lernenden. Betrachtet man nun im Masterplan Medizinstudium 2020 die zumindest für einige Fakultäten neuen Ausbildungsinhalte Arzt-Patienten-Kommunikation und Zusammenarbeit im interdisziplinären bzw. interprofessionellen Team sowie das wissenschaftliche Arbeiten, stellt sich die Frage, wieso diese im Sinne ebendiesen Constructive Alignments nicht auch im weiteren Verlauf als Prüfungsinhalte genannt werden. Während einerseits den Fakultäten hier ausreichend Gestaltungsspielraum zu belassen ist, entwertet die Nichtnennung dieser Prüfungsinhalte im gleichen Schriftzug deren Relevanz im Vergleich zu anderen Studieninhalten.

Für die Umsetzung gibt es glücklicherweise bereits Erfahrungen, z.B. patientenorientierte Kommunikation in entsprechend verfassten OSCE-Stationen zu prüfen [5], [6], oder in OSCE-Stationen standardisierte Teammitglieder einsetzen [7], [8]. Leider versäumt es das Maßnahmenpapier aber, die „Befähigung zum wissenschaftlichen Arbeiten“ genauer zu operationalisieren. Hier ist zu hoffen, dass die Fakultäten bei der Interpretation dieser Maßnahme nicht nur die forschungsorientierten Kompetenzen des *Gelehrten* (Scholar) berücksichtigen.

Geht es um die Prüfungsformate, die den Studieninhalten bzw. der Umsetzung der Maßnahmen dienlich sind, sind Gelassenheit und Zuversicht angebracht. Gelassenheit, weil die in der Literatur beschriebenen und in Deutschland teils pilotierten teils etablierten Formate zuerst einmal ausreichend scheinen, die Herausforderungen des Maßnahmenpapiers umzusetzen. Die Quintessenz der internationalen Literatur zu kompetenzorientierter Ausbildung in der Medizin kommt zum Schluss, dass es in diesem Zuge individuelle Bedürfnisse der Studierenden zu bedienen gilt, die Studierenden ihr Lernen aktiv managen und regelmäßiges Self-Assessment betreiben und deren Lernfortschritt

regelmäßig erhoben und zurückgemeldet werden muss [3]. Dies ist nichts als ein Plädoyer für mehr Progress Testing in Kombination mit Mentorenprogrammen, einer lebendigen Reflexions- und Feedbackkultur und Lern-Portfolios, alles Angelegenheiten, mit der die deutsche Hochschulmedizin, der Politik vielleicht vorgreifend, bereits Erfahrungen gesammelt hat [9], [10]. Gelassenheit auch deswegen, weil ein geeignetes Format der summativen kompetenzorientierten Prüfung – der bereits erwähnte OSCE – in Deutschland bereits weit verbreitet ist und an zahlreichen Fakultäten damit intensive Erfahrungen gemacht wurden: Ein Format, das dezidiert zur Prüfung klinischer Kompetenz gedacht ist [11] und in Variationen z.B. in der Schweiz [12], den USA [13] und in Kanada [14] einen Teil der Abschlussprüfung des Medizinstudiums darstellt. Es wäre wünschenswert, dass bei der Entwicklung der neuen Ärztlichen Prüfung alle Stakeholder, auch die Fakultäten aktiv in den Entwicklungsprozess einbezogen werden, was sich in der Schweiz als vorteilhaft erwies (12).

Selbstverständlich muss berücksichtigt werden, dass der logistische und finanzielle Aufwand, einen OSCE durchzuführen, erheblich ist [15], weswegen nicht vergessen werden sollte, dass etablierte schriftliche Prüfungsformate weiterhin zur Verfügung stehen. So z.B. sind Multiple Choice-Fragen und Key Feature-Fälle, soweit sie auf der Ebene Problemlösen prüfen (es sei an die Definition der Kompetenz erinnert), weiterhin relevante Formate - auch wenn der Aufwand, gute Fragen bzw. Fälle zu erstellen, auch hier hoch ist und die Erfahrung der meisten AutorInnen darin oft nur gering. Die elektronischen Unterstützung würde den Einsatz mancher dieser Formate zwar erleichtern, wirft aber selbst neue Fragen bzgl. Logistik und Finanzierung auf.

Und schließlich ist Zuversicht angebracht, da das Maßnahmenpapier erklärt, die Anzahl und Notenpflicht der Leistungsnachweise würde überprüft. Die schlichte Anzahl summativ durchzuführender Prüfungen, die nur bedingt lernförderlich scheinen, sondern wohl primär juristisch belastbaren Bestehens-/Nichtbestehens-Entscheiden dienen sollen, steht in eindeutigem Gegensatz zu einer Kultur des Assessment for Learning [16], also des Prüfens nicht mit dem Ziel, das Gelernte zu messen, als vielmehr mit dem Ziel, das Lernen zu unterstützen. Es bleibt zu hoffen, dass neben der Diskussion über die Benotung auch die Bewertung der Prüfungsleistung im Staatsexamen weiter erörtert wird. Zwar gibt es Noten in der Ärztlichen Prüfung bzw. ihren Vorläufern seit wenigstens 1869 [17], jedoch ist der vorrangige Zweck einer Abschlussprüfung, die Entscheidung zu ermöglichen, ob einE PrüfungskandidatIn die Mindestqualifikation besitzt, um unter Aufsicht der Weiterbildungsbefugten an RealpatientInnen weiter zu lernen. Es liegt auf der Hand, dass dies eine Ja/Nein-Frage ist, weswegen diese Prüfung dafür optimiert sein sollte, ebendiese Frage zu beantworten [18]. Der Aufgabenmix sanktionierender Prüfungen (Staatsprüfung, summative fakultäre Prüfungen) muss hierfür optimiert sein. Für diese gilt es auch, die Ablösung der bestenfalls historisch legitimierten 60%-Bestehensgrenze zu diskutieren und eine dem Geiste der Kompetenzorientierung angemessene Kriteriumsorientierung anzudenken. Im Sinne von Kanes Rahmenwerk zur Validierung von Tests umreisst der Masterplan Teile der sog. Interpretations-Debatte [19], [20] und gemeinsam mit dem IMPP muss auch die Validierungs-Debatte noch geführt werden.

Zwei Kuriositäten fallen noch auf: Einerseits, dass trotz der im Masterplan Medizinstudium 2020 geforderten Kompetenzorientierung von Lehre und Prüfungen immer noch eine Fächerlogik der Curricula postuliert wird, z.B. bei den in den mündlichen Ärztlichen Prüfungen zu berücksichtigenden Inhalten. Nun sind aber modernere Curricula (wie auch Kliniken) in Deutschland teilweise schon jenseits der Fächer angelangt. Der Beratungsanlass eines Patienten, weswegen er ärztliche Hilfe in Anspruch nimmt, ist schließlich ebenso wenig

fachorientiert wie die Kompetenzen, die das Lösen des Patientenproblems ermöglichen. Eine Loslösung von der Fächerlogik würde auch die Umsetzung des OSCE im Staatsexamen logistisch und psychometrisch wesentlich erleichtern, sodass nicht pro Wahlfach ein eigener Subtyp der Prüfung vorgehalten werden muss. Eine wahrlich kompetenzorientierte Prüfungskultur und die Überarbeitung des NKLM sollten Restriktionen einer erzwungenen Fächerzuordnung tunlichst vermeiden. Kurios als zweites, dass mit dem Masterplan Medizinstudium 2020 erstmals das Format eines universitären Leistungsnachweises vorgegeben wird und damit den Fakultäten ein Freiheitsgrad abgerungen wird.

Beigetragen von (alphab.): Daniel Bauer, Monika Himmelbauer, Maren März

Literaturverzeichnis

1. Fischer MR, Bauer D, Mohn K, NKLM-Projektgruppe. Finally finished! National Competence Based Catalogues of Learning Objectives for Undergraduate Medical Education (NKLM) and Dental Education (NKLZ) ready for trial. GMS Z Med Ausbild. 2015;32(3):Doc35. doi: 10.3205/zma000977
2. Weinert FE. Vergleichende Leistungsmessung in Schulen - eine umstrittene Selbstverständlichkeit. In: Weinert FE, ed. Leistungsmessungen in Schulen. Weinhein, Basel: Beltz Verlag; 2001. p.17-32
3. Frank JR, Mungroo R, Ahmad Y, Wang M, De Rossi S, Horsley T. Toward a definition of competency-based education in medicine: a systematic review of published definitions. Med Teach. 2010;32(8):631-637. doi: 10.3109/0142159X.2010.500898
4. Biggs J. Enhancing teaching through constructive alignment. High Educ. 1996;32(3):347-364.
5. Bachmann C, Hölzer H, Dieterich A, Fabry G, Langewitz W, Lauber H, Ortwein H, Pruskil S, Schubert S, Sennekamp M, Simmenroth-Nayda A, Silbernagel W, Scheffer S, Kiessling C. Longitudinales, bologna-kompatibles Modell-Curriculum" Kommunikative und soziale Kompetenzen": Ergebnisse eines interdisziplinären Workshops deutschsprachiger medizinischer Fakultäten. GMS Z Med Ausbild. 2009;26(4):Doc38. doi: 10.3205/zma000631
6. Cömert M, Zill JM, Christalle E, Dirmaier J, Härter M, Scholl I. Assessing communication skills of medical students in objective structured clinical examinations (OSCE)-A systematic review of rating scales. PloS one. 2016;11(3):e0152717. doi: 10.1371/journal.pone.0152717
7. Guise J-M, Deering SH, Kanki BG, Osterweil P, Li H, Mori M, Lowe NK. Validation of a tool to measure and promote clinical teamwork. Simul Healthc. 2008;3(4):217-223. doi: 10.1097/SIH.0b013e31816fdd0a
8. Wright MC, Phillips-Bute BG, Petrusa ER, Griffin KL, Hobbs GW, Taekman JM. Assessing teamwork in medical education and practice: relating behavioural teamwork ratings and clinical performance. Med Teach. 2009;31(1):30-38. doi: 10.1080/01421590802070853
9. Meinel FG, Dimitriadis K, von der Borch P, Störmann S, Niedermaier S, Fischer MR. More mentoring needed? A cross-sectional study of mentoring programs for medical students in Germany. BMC Med Educ. 2011;11(1):68. doi: 10.1186/1472-6920-11-68
10. Nouns ZM, Georg W. Progress testing in German speaking countries. Med Teach. 2010;32(6):467-470. doi: 10.3109/0142159X.2010.485656
11. Harden R, Stevenson M, Downie WW, Wilson G. Assessment of clinical competence using objective structured examination. Br Med J. 1975;1(5955):447-451.

12. Guttormsen S, Beyeler C, Bonvin R, Feller S, Schirlo C, Schnabel K, Schurter T, Berendonk C. The new licencing examination for human medicine: from concept to implementation. Swiss Med Wkly. 2013;143:w13897. doi: 10.4414/smw.2013.13897. eCollection 2013
13. Papadakis MA. The Step 2 clinical-skills examination. N Engl J Med. 2004;350(17):1703-1705.
14. Reznick R, Smee S, Rothman A, Chalmers A, Swanson D, Dufresne L, Lacombe G, Baumber J, Poldre P, Levasseur L, et al. An objective structured clinical examination for the licentiate: report of the pilot project of the Medical Council of Canada. Acad Med. 1992;67(8):487-494.
15. Brown C, Cleland J, Walsh K. The costs of medical education assessment. Med Teach. 2016;38(2):111-112. doi: 10.3109/0142159X.2015.1105946
16. Schuwirth LW, Van der Vleuten CP. Programmatic assessment: from assessment of learning to assessment for learning. Med Teach. 2011;33(6):478-485. doi: 10.3109/0142159X.2011.565828
17. Rentschler HE. Zur Geschichte der ärztlichen Prüfungen. Med Ausbild. 1988;5(2):77-82. Zugänglich unter/available from: https://gesellschaft-medizinische-ausbildung.org/publizieren/zma-archiv/1988.html
18. AERA, APA, NCME. The Standards for Educational and Psychological Testing. Washington, DC: American Psychological Association; 2013. Zugänglich unter/available from: https://www.apa.org/science/programs/testing/standards
19. Kane M. Validating high-stakes testing programs. Educ Measurement. 2002;21(1):31-41. doi: 10.1111/j.1745-3992.2002.tb00083.x
20. Fischer V. Gütekriterien bei universitären Prüfungen im Lichte von Kanes Rahmenwerk. Wien Med Wochenschr. 2019;169(5-6):110-118. doi: 10.1007/s10354-018-0661-z
