# Supplementary material for: Position of the advisory and executive board of the German Association for Medical Education (GMA) regarding the “masterplan for medical studies 2020”
Source: GMS J Med Educ. 2019 Aug 15;36(4):Doc46. doi: 10.3205/zma001254 (PMC6737258; doi:10.3205/zma001254)
Supplement: Teaching Evaluation Committee [german] [file JME-36-4-46-s-014.pdf]

Stellungnahme des Ausschusses Lehrevaluation

Im Masterplan Medizinstudium 2020 werden Maßnahmen aufgeführt, die mit curricularen Änderungen einhergehen werden. Eine gut begründete und sorgfältig konzipierte Evaluation ist dafür unabdingbar. Evaluation ist hier im Sinne von Cronbach zu verstehen, indem Informationen gesammelt und verarbeitet werden mit dem Ziel, Entscheidungen über ein Curriculum zu fällen [1]. Diese Entscheidungen können Curriculumverbesserungen betreffen, auf Individuen bezogen sein (z.B. Ermittlung der Bedürfnisse, Lernfortschritt) und/oder sich auf administrative Regelungen beziehen. Nach Gibson werden des Weiteren vier grundlegende Dimensionen der Lehrqualität definiert (Strukturen, Prozesse, Lehrende und „Outcomes“) [2]. Diese werden von den im Masterplan Medizinstudium 2020 genannten Maßnahmen beeinflusst und sollten entsprechend auch Gegenstand der begleitenden Evaluation sein.

Da die Evaluation künftig nicht nur der fakultätsinternen Bewertung der Lehre dienen wird, sondern insbesondere auch zur Erfolgskontrolle und Bewertung der politischen Maßnahmen notwendig ist, sollten frühzeitig fakultätsübergreifende Evaluationsziele und -methoden konsentiert werden. Dies ist wichtig, damit Daten erhoben werden können, die den Nachweis bringen, ob und inwiefern die geforderten Maßnahmen tatsächlich wirksam sind. Die Evaluationskonzepte müssen sich an internationalen Standards orientieren, und es ist empfehlenswert, die (Neu-)Entwicklung geeigneter Evaluationsinstrumente auch wissenschaftlich zu begleiten. Hierfür müssen adäquate finanzielle Ressourcen zur Verfügung gestellt werden. Eine zentrale Koordination durch den MFT unter Einbindung der GMA (z.B. Ausschuss Lehrevaluation und Ausschuss für Methodik der Ausbildungsforschung) ist empfehlenswert. Zusätzlich sollten alle Maßnahmen bereits vor einer Neufassung der ÄApprO unter dem Aspekt der Anwendbarkeit, der Wirksamkeit bzw. Übertragbarkeit kritisch beleuchtet werden. Ein wichtiger Aspekt, der vor Verabschiedung der neuen ÄApprO im Fokus stehen sollte, sind die Kosten-Nutzen-Relationen der angestrebten Maßnahmen.

Im Folgenden wird auf einzelne konkrete Maßnahmen eingegangen, für die eine begleitende Evaluation wesentlich sein wird.

<1> Die ärztliche Ausbildung wird kompetenzorientiert ausgestaltet

Die Ausgestaltung der ärztlichen Ausbildung anhand von Kompetenzen muss sich einerseits in der Etablierung kongruenter Prüfungen niederschlagen (Ergebnis-Ebene). Zugleich aber muss im Rahmen der begleitenden Evaluation zuverlässig untersucht werden, ob der Schritt von einer primär wissensbasierten zu einer kompetenzbasierten Ausbildung auch auf struktureller und prozeduraler Ebene vollzogen wurde. Als mögliches Instrument zur Erhebung der hierfür benötigten Daten eignen sich fakultätsübergreifende Befragungen. Auch studentische Evaluationen können zu diesem Zweck eingesetzt werden.

<5> Die Studien- und Prüfungsinhalte werden künftig stärker auf die wesentlichen Lernziele fokussiert.

In dieser Maßnahme wird die Passung zwischen Lernzielen und Lehraktivitäten („constructive alignment“) angesprochen. Es existieren Evaluationsinstrumente, mit denen das Ausmaß dieser Passung anhand studentischer Einschätzungen erfasst werden kann; diese Instrumente werden jedoch bisher im Medizinstudium kaum eingesetzt. Neben der studentischen Evaluation kann auch die o.g. Befragung der medizinischen Fakultäten

Aufschluss darüber geben, welche Maßnahmen zur Erreichung der hier genannten Ziele ergriffen wurden.

<10> Zur strukturierten Vermittlung wissenschaftlicher Kompetenzen wird künftig ein Leistungsnachweis vorgegeben. Grundlage dafür sind die Empfehlungen des Wissenschaftsrates.

Die Vermittlung wissenschaftlicher Kompetenzen wird im Medizinstudium künftig deutlich an Gewicht gewinnen. Aktuell fehlen Prüfungs- und Evaluationsmethoden, mit denen die Qualität der diesbezüglichen Ausbildung reliabel und valide überprüft werden könnten. Daher ist es erforderlich, parallel zur Entwicklung der Lehrformate (die sich nicht in Laborrotationen erschöpfen sollten) auch zum Lernziel kongruente Prüfungs- und Evaluationsinstrumente zu entwickeln. Die Evaluation der Qualität der Lehre sollte in erster Linie durch Studierende erfolgen; dabei sollte ein besonderer Schwerpunkt auf das Lehr-/Lernergebnis liegen. Im Gegensatz hierzu sollte die strukturelle Verankerung der neuen Lehre im Curriculum im Rahmen fakultätsübergreifender Befragungen evaluiert werden.

<15> Lehrpraxen werden verstärkt in die ärztliche Ausbildung einbezogen.

Nicht alle in Lehrpraxen tätigen Ärztinnen und Ärzte sind medizindidaktisch qualifiziert. Eine Evaluation der hier erbrachten Lehrleistung ist besonders wichtig, um einerseits die Qualität der Lehre überprüfen zu können und andererseits gezielte Fortbildungsangebote für Lehrende ohne weitergehende didaktische Qualifikation erarbeiten zu können. Problematisch ist, dass je Praxis nur eine geringe Anzahl von Studierenden unterrichtet wird. Dies stellt eine quantitative Evaluation hinsichtlich der Reliabilität und Validität vor schwierige Aufgaben. Es erscheint notwendig, speziell für dieses Setting qualitative Evaluationsmethoden zu entwickeln, die auch eine enge Ankopplung an die jeweils zuständige Fakultät einschließen.

<27> Das IMPP entwickelt für die Ein- und Durchführung der OSCE-Prüfungen in der Ärztlichen Prüfung verbindlichen Vorgaben; dies beinhaltet auch die Standardisierung der mündlich-praktischen Prüfung am Patientenbett und Vorgaben zur Prüferqualifizierung.
<28> Wir erwarten, dass die Universitäten diese Vorgaben in der Qualifizierung und Fortbildung der Lehrenden sowie der Prüferinnen und Prüfer nachvollziehen.

Die Qualitätssicherung der hier benannten Schritte ist auch eine Form der Evaluation. Sofern qualitätssichernde Maßnahmen nicht als inhärenter Bestandteil der Prüfungsentwicklung vorgesehen sind, müssen sie mittels geeigneter (und auf die Prüfungsinhalte und -formate abgestimmter) Weise konzipiert und implementiert werden. Insbesondere die Standardisierung der Prüfungen sowie die Qualität der Prüferqualifizierung im Rahmen von Faculty Development-Maßnahmen sind einer quantitativen und qualitativen Evaluation zugänglich. Es wird empfohlen, diese qualitätssichernden Maßnahmen standortübergreifend zu konzipieren.

<33> Wir erwarten, dass die Hochschulen stärker als bisher von der Möglichkeit der Approbationsordnung für Ärzte Gebrauch machen und ihre Gestaltungsspielräume nutzen, um mehr Lehrkrankenhäuser auch im ländlichen Raum dauerhaft einzubinden.

Analog zu den zum Punkt <15> angestellten Überlegungen sind auch an Krankenhäusern, an denen die Voraussetzungen für die Anerkennung als akademisches Lehrkrankenhaus hinsichtlich der didaktischen Qualifikation der Ärztinnen und Ärzte nicht vollständig erfüllt sind, entsprechende fakultätsentwickelnde Maßnahmen anzustoßen und ihre Effektivität zu evaluieren. Dies schließt nicht nur eine Evaluation der Fakultätsentwicklungsprogramme

(hier: medizindidaktische Trainings) ein, sondern auch eine Beurteilung der Qualität der Lehre, die von den hier qualifizierten Ärztinnen und Ärzten geleistet wird. Diese Evaluation wird sich in erster Linie auf studentisches Feedback stützen; eine zentrale Organisation der Datenerhebung und -analyse erscheint ratsam.

Vor allem bezüglich der Punkte <10> und <15> ist die anstehende Entwicklungsarbeit erheblich und ohne eine entsprechende Finanzierung nicht umsetzbar. Für die fakultätsübergreifenden Evaluationen müssen Strukturen etabliert werden, die sicherstellen, dass einerseits Personal vorhanden ist, das über die erforderliche Kompetenz zur Datenerhebung und -analyse verfügt und die andererseits den Ansprüchen der Datenschutzgrundverordnung genügen können.

Beigetragen von (alphab.): Marianne Giesler, Tobias Raupach, Alexandra Scherg, Katrin Schüttpelz-Brauns

Literaturverzeichnis

1. Cronbach LJ. Evaluation zur Verbesserung von Curricula. In: Wulf C, ed. Evaluation Beschreibung und Bewertung von Unterricht, Curricula und Schulversuchen. München: R. Piper & Co. Verlag; 1972. p.41-59
2. Gibson KA, Boyle P, Black DA, Cunningham M, Grimm MC, McNeil HP. Enhancing evaluation in an undergraduate medical education program. Acad Med. 2008;83(8):787-793. doi: 10.1097/ACM.0b013e31817eb8ab
