# Supplementary material for: Position of the advisory and executive board of the German Association for Medical Education (GMA) regarding the “masterplan for medical studies 2020”
Source: GMS J Med Educ. 2019 Aug 15;36(4):Doc46. doi: 10.3205/zma001254 (PMC6737258; doi:10.3205/zma001254)
Supplement: Educational Research Methodology Committee [german] [file JME-36-4-46-s-015.pdf]

Stellungnahme des Ausschusses Methodik der Ausbildungsforschung

Im Masterplan Medizinstudium 2020 werden Maßnahmen, die sich aus best-practice-Beispielen und aus der Forschung ergeben, gleichwertig neben politisch motivierten Maßnahmen aufgeführt. So erscheinen Maßnahmen, die sich z.B. auf die Nachwuchsgewinnung in der Allgemeinmedizin beziehen, eher politisch motiviert als evidenzbasiert (Maßnahmen Nrs. 16, 17.1, 20; [1]). Die Medizinstudierenden in Deutschland sind sich der Bedeutung des Faches Allgemeinmedizin bewusst und sie sind durchaus willens, viel Zeit ihres Studiums darauf zu verwenden [2]. Das lässt daher eher die Erhöhung des Anteils an Wahlveranstaltungen der Allgemeinmedizin im Studium sinnvoll erscheinen [3]. Eine Verpflichtung aller Studierenden könnte sich ggf. sogar nachteilig auswirken, wenn z.B. der Eindruck entstünde, mit der Betonung der Allgemeinmedizin sei zunächst einmal eine Einschränkung der per se geringen (Wahl-) Freiheiten im Studium verbunden.

Andererseits muss das bloße Umsetzen als erfolgreich beschriebener Maßnahmen nicht zum Erfolg führen. Bereits in der ersten Leitlinie zu „Best Evidence Medical Education“ wurde betont, dass die Übertragbarkeit von Forschungsergebnissen und Konzepten stets zu überprüfen ist [4]. Dabei ist zum einen zu berücksichtigen, ob die Rahmenbedingungen („Setting“) vergleichbar sind. Zum anderen muss sichergestellt sein, dass es nicht zu (ungewollten) Modifikationen des zu übertragenden Ansatzes kommt, die die Wirksamkeit der Maßnahme gefährden. Daher können Ergebnisse, die im Rahmen der Ausbildungsforschung gewonnen werden, nicht generell übernommen werden, zumindest nicht ohne den Erfolg des Transfers durch Evaluation und ggf. wissenschaftliche Begleitung sicherzustellen.

In Maßnahme Nr. 29 („Ergänzend sind die Hochschulen aufgefordert, neue kompetenzorientierte Lehr- und Prüfungsformate zu erproben und weiterzuentwickeln“) wird dies als „Anwendungsforschung“ aufgegriffen [1]. Jedoch ist es unklar, inwiefern die angesprochene Zielgruppe in der Lage ist, diese Anforderungen kompetent zu erfüllen. Gibt es genügend personelle und materielle Ressourcen bzw. methodische Expertise, um die Effektivität neuer Lehr- und Prüfungsformate adäquat zu überprüfen? Falls dies nicht der Fall ist, bleibt diese Forderung nach der „Anwendungsforschung“ an der Oberfläche und ein reines Lippenbekenntnis, ohne den Anspruch zu erfüllen, wissenschaftliche Erkenntnis zu generieren. So konnten Reed et al. anhand von 210 Studien in der Medizinischen Ausbildungsforschung einen Zusammenhang zwischen der finanziellen Förderung der Studie und deren Qualität (Expertenrating, Zitationsrate, sowie Höhe des Impact Factors) aufzeigen [5].

Die vom Wissenschaftsrat bereits 2014 geforderte Stärkung und Ausweitung der Ausbildungsforschung [6] sollte daher durch die Einrichtung von entsprechenden Lehrstühlen, Instituten und Kompetenzzentren an den Medizinischen Fakultäten realisiert werden. Es ist wünschenswert, dass das BMBF Drittmittel für die wissenschaftliche Begleitung und Evaluation bereitstellt, da aktuell keine ausreichenden Ressourcen für die wissenschaftliche Überprüfung des etwaigen Effekts einer implementierten Maßnahme zur Verfügung stehen.

Gleichzeitig sollte darauf geachtet werden, den Masterplan nicht als alleinige Agenda für medizinische Ausbildungsforschung in Deutschland zu verwenden, um den Anschluss an internationale Forschung im Bereich von Medizindidaktik und Lehre nicht zu verpassen. An dieser Stelle bietet sich andererseits aber auch die Chance, Forschung zu betreiben, die

international von Interesse sein könnte und dadurch Deutschland als Standort von Ausbildungsforschung international sichtbar zu machen. Eine nachhaltige Institutionalisierung von Ausbildungsforschung sollte daher ein langfristiges Ziel sein.

Aus dem Vorherigen ergeben sich aus unserer Perspektive kurz gefasst folgende Empfehlungen:

- Neuerungen bzw. damit einhergehende Maßnahmen (z.B. Einführung von zusätzlichen bzw. alternativen Unterrichtsformaten) müssen durch nach Möglichkeit evidenzbasierte Minimalanforderungen definiert sein, aber ausreichend Spielraum bieten, um an z.B. lokale Gegebenheiten in Abstimmung mit dem MFT unter Einbindung der GMA angepasst zu werden.
- Eine Institutionalisierung der wissenschaftlichen Begleitung der Maßnahmen des Masterplans Medizinstudium 2020 inkl. der nachhaltigen Bereitstellung personeller und finanzieller Ressourcen ist unabdingbar, um den Prozess langfristig zu begleiten und wenn möglich noch vor Einführung der Novelle der ÄApprO zu modifizieren.

Beigetragen von (alphab.): Marianne Giesler, Wolf Hautz, Monika Himmelbauer, Jörg Marienhagen, Jan Matthes, Katrin Schüttpelz-Brauns

Literaturverzeichnis

1. Bundesministerium für Bildung und Forschung. Masterplan Medizinstudium 2020. Berlin: Bundesministerium für Bildung und Forschung; 2017. Zugänglich unter/available from: https://www.bmbf.de/files/2017-03-31_Masterplan%20Beschlusstext.pdf
2. Dafsari HS, Herzig S, Matthes J. A multi-centre student survey on weighing disciplines in medical curricula–a pilot study. GMS J Med Educ. 2017;34(2):Doc24. doi: 10.3205/zma001101
3. Jacob R, Kopp J, Schultz S. Berufsmonitoring Medizinstudenten 2014–Ergebnisse einer bundesweiten Befragung. Berlin: Kassenärztliche Bundesvereinigung (KBV); 2015.
4. Harden R, Grant J, Buckley G, Hart I. BEME Guide No. 1: Best evidence medical education. Med Teach. 1999;21(6):553-562.
5. Reed DA, Cook DA, Beckman TJ, Levine RB, Kern DE, Wright SM. Association between funding and quality of published medical education research. JAMA. 2007;298(9):1002-1009.
6. Wissenschaftsrat. Empfehlungen zur Weiterentwicklung des Medizinstudiums in Deutschland auf Grundlage einer Bestandsaufnahme der humanmedizinischen Modellstudiengänge. Dresden: Wissenschaftsrat; 2014.
